# 5-Year conditional relative survival of adolescents and young adults with solid malignancies in the Netherlands: a population-based cohort study

**DOI:** 10.1016/j.lanepe.2025.101429

**Published:** 2025-08-13

**Authors:** Noelle J.M.C. Vrancken Peeters, Daniël J. van der Meer, Bo Wardenier, Marion L'hôte, Eveliene Manten-Horst, Henrike E. Karim-Kos, Winette T.A. van der Graaf, Olga Husson

**Affiliations:** a Department of Medical Oncology, Netherlands Cancer Institute - Antoni van Leeuwenhoek, Amsterdam, the Netherlands; b Department of Surgical Oncology, Erasmus MC Cancer Institute, Erasmus University Medical Centre, Rotterdam, the Netherlands; c European Cancer Organisation, Brussels, Belgium; d Dutch AYA ‘Young & Cancer’ Care Network, Amsterdam, the Netherlands; e Department of Research and Development, Netherlands Comprehensive Cancer Organization (IKNL), Utrecht, the Netherlands; f Department of Medical Oncology, Erasmus MC Cancer Institute, Erasmus University Medical Centre, Rotterdam, the Netherlands; g Department of Public Health, Erasmus MC Cancer Institute, Erasmus University Medical Centre, Rotterdam, the Netherlands

**Keywords:** Adolescent and young adult oncology, Conditional survival, Right to be forgotten

## Abstract

**Background:**

The growing number of adolescent and young adult (AYA) cancer survivors highlights the importance of their reintegration into society. However, years after recovery, cancer survivors still need to disclose their cancer history when applying for insurance. Right to be forgotten (RTBF) laws aim to protect cancer survivors from financial discrimination by shortening the required disclosure period. The required period for a disease to be forgotten varies between countries up to ten years in the Netherlands. Currently, questions arise about whether existing RTBF laws disadvantage AYAs by grouping them with older patients. This observational study examined the long-term conditional relative survival (CRS) of AYA solid malignancy survivors to inform potential adjustments to RTBF laws.

**Methods:**

Data of all invasive solid malignancies diagnosed within the AYA population between 1998 and 2021 were obtained from the Netherlands Cancer Registry (NCR). The five-year CRS was calculated annually up to ten years post-diagnosis, using a hybrid analysis approach for the entire AYA solid malignancy cohort, and stratified by sex, age, and cancer type.

**Findings:**

The entire AYA solid malignancy survivor cohort (n = 71,973) reached the threshold of minimal excess mortality (CRS > 95%) four years post-diagnosis. For AYA survivors of germ cell and trophoblastic tumors (ovarian and testis), malignant melanoma, thyroid carcinoma, skin carcinoma, and neuroendocrine tumors (NET), the excess mortality was minimal (CRS > 95%) since diagnosis. Most other cancer types reached the 95% threshold within five or ten years post-diagnosis.

**Interpretation:**

These results can serve as a basis for the reappraisal of current RTBF regulations for AYA solid malignancy survivors in the Netherlands, which can have important implications for other European countries with RTBF policies.

**Funding:**

This research received no specific grant from any funding agency in the public, commercial, or not-for-profit sectors.

**Research in context**

**Evidence before this study**

The number of AYA cancer survivors is increasing, underscoring the importance of their active reintegration into society. However, due to the multiple forms of financial discrimination they face, returning to life in society can be very demanding. Cancer survivors need to disclose their cancer history when applying for insurance, even many years after their recovery. Right to be forgotten (RTBF) laws aim to protect cancer survivors from financial toxicity by shortening this required disclosure period. Currently, questions arise about whether existing RTBF laws disadvantage AYAs by grouping them with older patients. Up-to-date research on the long-term conditional relative survival (CRS) of AYA cancer survivors is needed to address this issue, yet such studies remain limited. A PubMed literature search for studies published within the last 10 years about conditional survival among Adolescents and Young Adults (AYAs) with cancer was performed on September 2023, using the search terms “AYA [tiab]” OR “Adolescent and Young Adult [tiab]” AND “Conditional Survival [tiab]”. Only two publications were identified, whereby the study by Ou et al. (2014) was limited to a small sample size, including only sarcomas and patients aged ≤25 years at diagnosis. To date, only Anderson et al. (2018) have thoroughly investigated the conditional survival of AYA cancer survivors, indicating the severe underrepresentation of this subject in AYA oncology literature.

**Added value of this study**

This large population-based cohort study (n = 71,973) aims to examine the long-term CRS of Dutch AYA cancer patients (aged 18–39 years) diagnosed with a solid tumor to inform potential adjustments to European RTBF laws. This is the second study to date that comprehensively investigates the conditional survival among AYA cancer survivors in general, whereby it is the first to do this within a European setting, adding highly needed information to the current AYA oncology literature. Findings of this study are also used to reflect upon existing RTBF laws to enable and strengthen discussions about current policies to ensure a more equitable insurance system for AYA survivors, which could set the basis for Europe wide initiatives. The total AYA cancer population reached the threshold of minimal excess mortality (CRS > 95%) after a survival period of four years. Additionally, the impact of cancer on survival decreased significantly with additional years survived. For AYA cancer patients with germ cell and trophoblastic tumors (ovarian and testis), malignant melanoma, thyroid carcinoma, skin carcinoma, and neuroendocrine tumors (NET), the excess mortality was minimal from the moment of diagnosis. Most other tumor types reached the 95% threshold within five or ten years post-diagnosis. This might be a first indication that the existing RTBF laws should be re-evaluated, and the required period to be forgotten could be reduced for AYA survivors of solid cancers.

**Implications of all the available evidence**

The results of this study can serve as a basis for the reappraisal of current RTBF regulations for AYA survivors of solid cancers. It is important to constantly re-evaluate the long-term survival of AYA cancer patients, given the development and improvement of novel treatments, to help them reintegrate successfully into society as soon as possible.

## Introduction

Due to the introduction of novel treatment options, the prognosis for cancer patients has steadily improved in the last decades.^1^^,^^2^ There are currently more than 20 million cancer survivors in Europe, and this is expected to increase annually by 3%.^1^^,^^3^ This underscores the crucial importance of successfully reintegrating cancer survivors into society.^4^ Due to the various physical and psychosocial challenges, as well as the multiple forms of financial discrimination cancer survivors experience, returning to life in society can be demanding. Many years after their recovery, cancer survivors are still required to disclose their history of prior cancer when applying for insurance, including life insurance and mortgages.^4^, ^5^, ^6^, ^7^, ^8^ Disclosing a history of cancer can lead to the dismissal of these financial services or admission under the condition of higher insurance premiums. Receiving insurance is an important protective factor against financial toxicity and is associated with better health outcomes among cancer survivors.^9^^,^^10^ New policies and legislative initiatives are needed to protect cancer survivors from discrimination and support their active reintegration into society.^4^^,^^5^ In 2016, France was the first EU member state to establish the right to be forgotten (RTBF), which protects cancer survivors from financial discrimination by eliminating the requirement to disclose a cancer history when applying for insurance under certain conditions. To date, only eight EU member states have implemented national RTBF laws for cancer survivors, including the Netherlands following initiative by the Dutch Federation of Cancer Patients Organizations (NFK).^11^ The required period for a medical history to be forgotten varies significantly between countries, ranging from five to ten years. In the Netherlands, Portugal, Italy, and Cyprus, a medical history would be forgotten after ten years, which in the Netherlands was partly informed by research by van Maaren et al.^12^^,^^13^ In Belgium this is the case after eight years, in Romania and Slovenia after seven years, and in France and Spain after five years, as long as there is no evidence of relapse and recurrence.^4^, ^5^, ^6^, ^7^^,^^11^^,^^14^

Especially adolescent and young adult (AYA) cancer patients, broadly defined as patients aged 15–39 years at the time of their initial cancer diagnosis, have prolonged financial difficulties after their recovery and experience difficulties with applying for insurance.^15^, ^16^, ^17^, ^18^ AYAs are a distinct group in oncology that is diagnosed with an unique array of cancer types, including those most commonly seen before age 15 during childhood (e.g., acute lymphoblastic leukemia, Ewing sarcoma), those most commonly seen among older adults aged above 39 years (e.g., colorectal, lung and breast carcinomas), and cancer types that have their peak incidence among those belonging to the AYA age-range (e.g., testicular carcinomas).^19^ The AYA population is further distinct, not only because of the unique biological characteristics of their cancers, but also due to the specific psychosocial and socioeconomic issues they face compared to younger and older cancer patients.^16^^,^^20^, ^21^, ^22^ Adolescence and young adulthood are challenging life phases characterized by significant physical and emotional transitions, such as engaging in serious and intimate relationships, starting a full-time job, and planning to have children.^21^^,^^22^ Despite these challenges, the survival of AYA cancer patients has significantly improved over time, with five-year relative survival outcomes now being well-above 80% in high-income countries for all cancers combined.^19^^,^^23^ Moreover, despite noticeable differences between cancer types, AYA cancer patients overall have higher five-year relative survival probabilities compared to older cancer patients, whereby a 17% point-estimate difference (82.5% vs. 65.9%) was reported by Keegan et al.^23^ This likely relates to the aforementioned differences in cancer type distribution, which is supported by the observed variation in age-related survival differences between cancer types.^23^

Currently, the question is being raised whether AYAs are disadvantaged by existing RTBF laws, grouping them with older cancer survivors. To address this issue, research on the long-term conditional relative survival (CRS) of AYA cancer patients is needed. Standard “unconditional” relative survival estimates that are calculated from the time of diagnosis are influenced by AYA cancer patients who die during the first year post-diagnosis. These estimations are less clinically relevant for patients who already survived several years.^24^, ^25^, ^26^, ^27^ CRS estimates, defined as the likelihood of survival given that an individual has already survived a certain period post-diagnosis, only include patients still alive at a specific time point. Therefore, they provide a more pragmatic estimation of long-term survival.^25^, ^26^, ^27^, ^28^ Nevertheless, literature on these CRS estimates for AYA cancer patients is scarce and missing in Europe.^24^ Therefore, this large population-based Dutch cohort study (n = 71,973) aims to comprehensively investigate the CRS of AYA solid malignancy survivors (e.g., breast, lung, colorectal cancers), specifically looking at differences based on sex, age at diagnosis and cancer type to best represent the current clinical landscape, which depends to a large extent on these factors. Findings of this study could serve as an initial foundation to enable and strengthen discussions about current RTBF policies in the Netherlands to ensure a more equitable insurance system for AYA survivors, which could also set the basis for Europe wide initiatives. Additionally, the results can enhance expectation management and enable better decision-making regarding follow-up care and surveillance strategies by providing healthcare professionals and patients with clinically relevant long-term survival outcomes.

## Methods

### Data sources

Data on cancer incidence and survival of all AYAs diagnosed with invasive solid malignancies in the Netherlands between 1989 and 2021 were obtained from the population-based Netherlands Cancer Registry (NCR; ≥95% nationwide coverage since 1989).^29^ The NCR, hosted by the Netherlands Comprehensive Cancer Organization (IKNL), collects detailed population-based data on new cancer cases in the Netherlands through the National Pathological Archive (PALGA), which is supplemented by information from the Dutch hospital database and various hematology laboratories. Patient and tumor details are systematically collected from medical records by trained registrars of the NCR. Tumors within the NCR during the incidence period were coded based on the International Classification of Diseases for Oncology (ICD-O), using the second edition (1993–2000) and third edition (since 2001).^30^ Staging of solid malignancies within the NCR is based on the Union for International Cancer Control (UICC) TNM-classification of malignant tumors in its fourth edition (1989–1998), fifth edition (1999–2002), sixth edition (2003–2009), seventh edition (2010–2016), and since 2017 all tumors are coded based on the eighth edition. Gynecological tumors are staged based on the international Federation of Gynecology and Obstetrics (FIGO) staging system, which is incorporated in all editions of the TNM-classification.^31^ Information on vital status (i.e., dead, alive, emigrated) within the NCR is obtained through annual linkage with the Nationwide Personal Records database (BRP, last linkage on 31-01-2023).

### Study population

All individuals aged 18 to 39 and first diagnosed with an invasive solid malignancy in the period 1998–2021 in the Netherlands were included. AYAs diagnosed between 1989 and 1997 (n = 24,198) did not contribute to the survival calculations and were therefore excluded from the study. This will be discussed in more detail shortly. In the Netherlands, AYAs are defined as patients aged between 18 and 39 years at the time of their primary cancer diagnosis because cancer care for adults (≥18 years) is scattered throughout diverse hospital departments in the Netherlands (n = 69 hospitals), while childhood cancer care (<18 years) was centralized in seven university hospitals, and since 2018 is concentrated in one center.^32^ The NCR does not collect data about basal-cell skin and lip carcinomas. Cases with a history of any other solid cancer and those diagnosed with multiple tumors (n = 4321) were excluded. See Appendix Table 1 for the baseline population characteristics.

### Diagnostic classification

Solid malignancies in this study were grouped according to the histology-based classification scheme developed by Barr and colleagues for cancers at AYA age. Detailed information about the tumor groups is provided elsewhere.^33^

### Statistical analyses

Descriptive statistics were used to describe the study population's patient and tumor characteristics.

#### Unconditional survival estimates

Standard “unconditional” relative survival estimates up to 15 years post-diagnosis were calculated with the strs Stata command developed by Dickman and colleagues. The relative survival is a common estimator for cancer-specific survival in the absence of reliable cause of death information that is calculated by dividing the observed survival of patients by the expected survival of an age-, sex-, and period-matched cohort from the general population.^34^ The default Ederer II method was used to compute the expected survival from Dutch general population lifetables retrieved from Statistics Netherlands (http://statline.cbs.nl) that contained annual survival probability data for both sexes aged 0–95 years between 1950 and 2024.^34^ The validated hybrid approach developed by Brenner and Rachet was used to obtain long-term outcomes that best reflect the survival experience of current-day patients in situations where the follow-up period extends beyond the diagnostic period for which incident cases are available (registration delay) by allowing different moments at which individuals become at risk based on their date of diagnosis. As such, it combines elements of both the classical cohort and period approach.^35^ For the current study, incidence data were available up to 2021. Survival follow-up extended to January 2023, but was truncated at 31 December 2022, so that one-year follow-up information was potentially available for all years included. To obtain recent long-term outcomes with sufficient precision (avoiding sparseness of data), a period window from 2012 to 2022 was selected. The point at which AYAs became at risk (start period window) was defined as the date of diagnosis for AYAs diagnosed between 1 January 2012 and 31 December 2021. For AYAs diagnosed before 2012, the start of the time at risk was 1 January 2013.

#### Conditional survival estimates

To investigate the changing mortality risk with time since diagnosis, the five-year CRS, defined as the probability of surviving an additional five years after a specific time post-diagnosis, was calculated annually up to 10 years after diagnosis, with patients diagnosed between 1998 and 2021 contributing their survival time to the required 15 years follow-up period within the selected period window (Appendix Figure 1). Excess mortality thresholds were defined based on previously established criteria and classified as substantial (CRS < 90%), little (CRS between 90% and 95%), and minimal (CRS > 95%).^24^^,^^26^ Given that the strs package does not provide CRS estimates, post-estimation procedures were necessary to obtain these statistics, including standard errors and resulting 95% confidence intervals (CIs). CRS estimates were calculated, using the unconditional relative survival estimates by applying standard conditional probability theory (i.e., the probability that some outcome (A) occurs given that another event (B) has occurred).

For instance, the five-year CRS given that someone has already survived for six years after diagnosis was calculated by dividing the unconditional relative survival outcome of 11 years by the six-year unconditional relative survival outcome. Standard errors were estimated by integrating previously proposed standard error definitions for conditional survival by Pokhrel et al. and Davis et al., resulting in the equation provided below.

$$\text{SE}\;\left(\hat{S}\left(t_{j}|t_{i}\right)\right)=\hat{S}\left(t_{j}|t_{i}\right)\sqrt{\frac{\text{SE}\;{\left[\hat{S}\left(t_{j}\right)\right]}^{2}}{\hat{S}{\left(t_{j}\right)}^{2}}\;-\;\frac{\text{SE}{\left[\hat{S}\left(t_{i}\right)\right]}^{2}}{\hat{S}{\left(t_{i}\right)}^{2}}}$$

here, ŝ(t_j_) and SE[ŝ(t_j_)], and ŝ(t_i_) and SE[ŝ(t_i_)] represent the unconditional relative survival and standard error estimates for the time intervals t_i_ and t_j,_ as provided by the strs package.^36^^,^^37^ Assuming a normal distribution, 95% CIs were then calculated by applying the standard formula: *95% CI = CRS ± 1.96 ∗ SE*.

All statistical analyses were performed with Stata/SE 17.0 (StataCorp LP, College Station, Texas) for the entire AYA study population combined and further stratified by sex, age at diagnosis (18–20, 21–24, 25–29, 30–34, 35–39 years), and by cancer type. Data about Race/ethnicity is unavailable within the NCR and was therefore excluded as stratifying factor. CRS estimates were only provided for cancer types with a minimum of n = 100 cases at risk for the calculation of the relative survival (Appendix Table 2).

### Ethics approval

This study was performed in accordance with the Declaration of Helsinki. Study approval was granted by the Privacy Review Board of the NCR (grant number: K23.261). Written informed patient consent was waived, as only deidentified patient information was included in this study.

### Role of the funding

This research received no specific grant from any funding agency in the public, commercial, or not-for-profit sectors.

## Results

### Population and tumor characteristics

In total, n = 71,973 AYA solid malignancy survivors were included. The majority of these patients were females (n = 44,689 [62.1%]). The median age at diagnosis was 34 years, with an interquartile range (IQR) of 29–37 years, and the median survival time was 8.9 years, with an IQR of 3.6–15.9 years. Most AYA cancer patients were diagnosed with stage I (n = 35,532 [49.4%]) and stage II (n = 14,581 [20.3%]) malignancies. Testicular germ cell and trophoblastic tumors (n = 10,792 [15.0%]), malignant melanomas (n = 14,137 [19.6%]), and carcinomas of the breast (n = 16,501 [22.9%]) were the most prevalent cancers. These and further baseline characteristics by sex are detailed in Table 1.

**Table 1:** Population and tumor characteristics of adolescents and young adults (AYAs, aged 18–39 years) diagnosed with an invasive solid malignancy in the Netherlands between 1998 and 2021.

	Both n = (%)	Males n = (%)	Females n = (%)
**Total**^a^	71,973 (100.0)	27,284 (100.0)	44,689 (100.0)
**Median age at diagnosis (IQR)**	34.0 (29.0–37.0)	32.0 (27.0–36.0)	34.0 (30.0–37.0)
**Age at diagnosis (years)**			
18–20	2413 (3.4)	1392 (5.1)	1021 (2.3)
21–24	5052 (7.0)	2807 (10.3)	2245 (5.0)
25–29	11,602 (16.1)	5436 (19.9)	6166 (13.8)
30–34	20,586 (28.6)	7562 (27.7)	13,024 (29.1)
35–39	32,320 (44.9)	10,087 (37.0)	22,233 (49.8)
**Median survival time (IQR)**	8.9 (3.6–15.9)	8.6 (3.3–15.6)	9.1 (3.8–16.1)
**Period of diagnosis (years)**			
1998–2005	23,245 (32.3)	8565 (31.4)	14,680 (32.9)
2006–2013	23,742 (33.0)	9195 (33.7)	14,547 (32.6)
2014–2021	24,986 (34.7)	9524 (34.9)	15,462 (34.6)
**Diagnostic group**^b^			
**CNS and other intracranial and intraspinal neoplasms**	3459 (4.8)	2035 (7.5)	1424 (3.2)
**Sarcomas**	3343 (4.6)	1712 (6.3)	1631 (3.7)
Soft tissue sarcomas	1785 (2.5)	870 (3.2)	915 (2.1)
Bone sarcomas	1078 (1.5)	603 (2.2)	475 (1.1)
Gastrointestinal stromal tumors (GIST)	156 (0.2)	87 (0.3)	69 (0.2)
Other sarcomas	324 (0.5)	152 (0.6)	172 (0.4)
**Blood and lymphatic vessel tumors**	439 (0.6)	327 (1.2)	112 (0.3)
**Nerve sheath tumors**	188 (0.3)	98 (0.4)	90 (0.2)
**Gonadal and related tumors**	12,650 (17.6)	11,109 (40.7)	1541 (3.5)
Testis, germ cell and trophoblastic	10,792 (15.0)	10,792 (39.6)	NA
Testis, non-germ cell	41 (0.1)	41 (0.2)	NA
Ovary, germ cell and trophoblastic	270 (0.4)	NA	270 (0.6)
Ovary, non-germ cell	1102 (1.5)	NA	1102 (2.5)
(Other) germ cell, non-germ cell, and trophoblastic tumors	445 (0.6)	276 (1.0)	169 (0.4)
**Melanoma, malignant**	14,137 (19.6)	4947 (18.1)	9190 (20.6)
**Carcinomas**	37,370 (51.9)	6851 (25.1)	30,519 (68.3)
Thyroid carcinoma	3183 (4.4)	742 (2.7)	2441 (5.5)
Carcinoma of head and neck	1450 (2.0)	828 (3.0)	622 (1.4)
Carcinoma of gastrointestinal tract	5729 (8.0)	2881 (10.6)	2848 (6.4)
Carcinoma of esophagus	236 (0.3)	173 (0.6)	63 (0.1)
Carcinoma of stomach	707 (1.0)	376 (1.4)	331 (0.7)
Carcinoma of small intestine	77 (0.1)	40 (0.2)	37 (0.1)
Carcinoma of colon	1821 (2.5)	879 (3.2)	942 (2.1)
Carcinoma of rectum	1079 (1.5)	583 (2.1)	496 (1.1)
Carcinoma of anus	120 (0.2)	62 (0.2)	58 (0.1)
Carcinoma of liver and intrahepatic bile ducts (IBD)	225 (0.3)	126 (0.5)	99 (0.2)
Carcinoma of gallbladder and other extrahepatic biliary	166 (0.2)	96 (0.4)	70 (0.2)
Carcinoma of pancreas	275 (0.4)	133 (0.5)	142 (0.3)
Other carcinoma of gastrointestinal tract	18 (0.0)	11 (0.0)	7 (0.0)
Carcinoma of lung, bronchus, and trachea	1293 (1.8)	634 (2.3)	659 (1.5)
Carcinoma of skin (if collected)^c^	949 (1.3)	425 (1.6)	524 (1.2)
Carcinoma of breast	16,501 (22.9)	37 (0.1)	16,464 (36.8)
Carcinoma of genital sites excluding ovary and testis	5899 (8.2)	69 (0.3)	5830 (13.1)
Carcinoma of uterine cervix	5246 (7.3)	NA	5246 (11.7)
Carcinoma of corpus uteri	288 (0.4)	NA	288 (0.6)
Carcinoma of vulva and vagina	279 (0.4)	NA	279 (0.6)
Carcinoma of penis	49 (0.1)	49 (0.2)	NA
Carcinoma of prostate	17 (0.0)	17 (0.1)	NA
Other genital	20 (0.0)	3 (0.0)	17 (0.0)
Carcinoma of urinary tract	1294 (1.8)	767 (2.8)	527 (1.2)
Carcinoma of kidney	917 (1.3)	552 (2.0)	365 (0.8)
Carcinoma of bladder	347 (0.5)	196 (0.7)	151 (0.3)
Other urinary	30 (0.0)	19 (0.1)	11 (0.0)
Other invasive carcinomas	736 (1.0)	335 (1.2)	401 (0.9)
Neuroendocrine tumors (NET)	1275 (1.8)	502 (1.8)	773 (1.7)
Neuroendocrine carcinomas (NEC)	66 (0.1)	33 (0.1)	33 (0.1)
Miscellaneous specified neoplasms	213 (0.3)	92 (0.3)	121 (0.3)
Unspecified malignant neoplasms except CNS	174 (0.2)	113 (0.4)	61 (0.1)
**Tumor stage (TNM & FIGO)**^d^			
Stage I	35,532 (49.4)	13,987 (51.3)	21,545 (48.0)
Stage II	14,581 (20.3)	3565 (13.1)	11,016 (25.0)
Stage III	8419 (11.7)	3331 (12.2)	5088 (11.4)
Stage IV	5011 (7.0)	2126 (7.8)	2885 (6.5)
Other/missing	8430 (11.7)	4275 (15.7)	4155 (9.3)

Abbreviations: IQR = Interquartile range, CNS = Central Nervous System, TNM = Tumor Node Metastasis, Figo = Fédération Internationale de Gynécologie et d'Obstétrique, NA = Not Applicable.

a Percentages might not add-up to 100% due to rounding.

b Cancer types were categorized into diagnostic groups according to the revised morphology based AYA-specific classification scheme developed by Barr et al. (2021).

c The Netherlands Cancer Registry does not collect data about basal-cell skin and lip carcinomas.

d Staging of solid malignancies was done according to the Union for International Cancer Control (UICC) TNM-classification of malignant tumors, varying from the fourth edition between 1989 and 1998 to the eighth edition since 2017. Gynecological tumors were staged based on the FIGO staging system, which is incorporated in all editions of the TNM-classification.

### Overall conditional relative survival

**Fig. 1:**
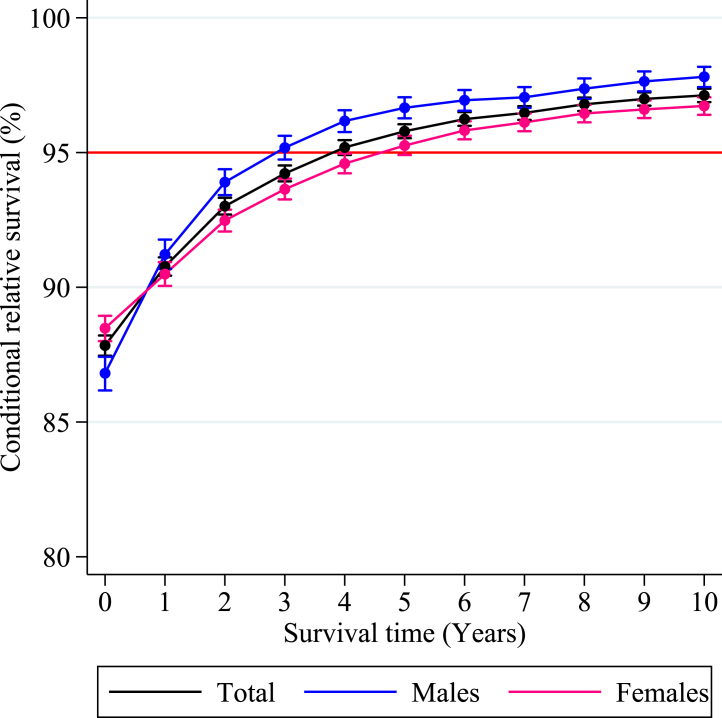
**Overall five-year conditional relative survival.** Five-year conditional relative survival (CRS) up to 10 years post-diagnosis of adolescents and young adults (AYAs, aged 18–39 years) diagnosed with an invasive solid malignancy in the Netherlands between 1998 and 2021 for the entire cohort (black), males (blue), and females (pink). CRS estimates were calculated using the hybrid approach for all types of solid malignancies combined. The error bars indicate the 95% confidence interval. The red line indicates the 95% minimal excess mortality threshold.

The five-year CRS for the entire AYA solid malignancy survivor cohort was 87.8% (95% CI: 87.5–88.2) at diagnosis and increased beyond the threshold of minimal excess mortality, i.e., a CRS of 95% or greater compared to the general population after having survived for four years. This threshold was achieved after three years among male and five years among female AYAs separately (Fig. 1 and Table 2).

**Table 2:** Five-year conditional relative survival with 95% confidence intervals up to 10 years post-diagnosis of adolescents and young adults (AYAs, aged 18–39 years) diagnosed with an invasive solid malignancy in the Netherlands between 1998 and 2021.

	CRS (95% CI)											CRS PE > 95% from year
**Survival years**	**0**^a^	**1**	**2**	**3**	**4**	**5**	**6**	**7**	**8**	**9**	**10**	
**Total**	87.8 (87.5–88.2)	90.8 (90.4–91.1)	93.0 (92.7–93.3)	94.2 (93.9–94.5)	95.2 (94.9–95.5)	95.8 (95.5–96.1)	96.2 (96.0–96.5)	96.5 (96.2–96.7)	96.8 (96.5–97.0)	97.0 (96.7–97.2)	97.1 (96.9–97.4)	4
**Sex**												
Males	86.8 (86.2–87.4)	91.2 (90.7–91.8)	93.9 (93.4–94.4)	95.2 (94.7–95.6)	96.2 (95.8–96.6)	96.7 (96.3–97.0)	96.9 (96.6–97.3)	97.0 (96.7–97.4)	97.4 (97.0–97.7)	97.6 (97.3–98.0)	97.8 (97.4–98.2)	3
Females	88.5 (88.0–88.9)	90.5 (90.1–90.9)	92.5 (92.1–92.9)	93.6 (93.3–94.0)	94.6 (94.2–95.0)	95.3 (94.9–95.6)	95.8 (95.5–96.2)	96.1 (95.8–96.5)	96.4 (96.1–96.8)	96.6 (96.3–96.9)	96.7 (96.4–97.1)	5
**Age at diagnosis (years)**												
18–20	89.4 (87.4–91.1)	92.4 (90.8–94.1)	94.5 (93.1–96.0)	95.8 (94.5–97.1)	96.5 (95.2–97.7)	97.3 (96.3–98.4)	97.5 (96.4–98.6)	98.1 (97.2–99.1)	98.0 (96.9–99.0)	98.5 (97.6–99.4)	98.7 (97.9–99.6)	3
21-24	90.8 (89.5–91.9)	93.1 (92.0–94.2)	95.2 (94.3–96.2)	96.4 (95.5–97.2)	97.3 (96.6–98.0)	97.4 (96.6–98.1)	97.3 (96.5–98.1)	97.4 (96.6–98.2)	97.8 (97.0–98.5)	97.7 (96.9–98.5)	98.4 (97.7–99.1)	2
25–29	89.6 (88.7–90.4)	92.2 (91.4–92.9)	94.2 (93.5–94.8)	95.4 (94.8–96.1)	96.1 (95.5–96.7)	96.6 (96.0–97.2)	97.2 (96.7–97.8)	97.4 (96.9–98.0)	97.5 (96.9–98.0)	97.8 (97.3–98.4)	98.0 (97.5–98.5)	3
30–34	88.7 (88.0–89.3)	91.3 (90.7–91.9)	93.4 (92.8–94.0)	94.6 (94.0–95.1)	95.6 (95.1–96.1)	96.1 (95.6–96.6)	96.4 (95.9–96.8)	96.5 (96.1–97.0)	97.0 (96.5–97.4)	97.3 (96.8–97.7)	97.4 (96.9–97.8)	4
35–39	85.9 (85.3–86.5)	89.3 (88.7–89.8)	91.8 (91.2–92.3)	93.0 (92.5–93.5)	94.1 (93.6–94.5)	94.9 (94.4–95.3)	95.5 (95.1–96.0)	95.8 (95.4–96.2)	96.2 (95.8–96.6)	96.3 (95.9–96.7)	96.4 (96.0–96.8)	6
**Diagnostic group**^b^												
**CNS and other intracranial and intraspinal neoplasms**	65.7 (63.1–68.2)	68.4 (65.7–71.0)	72.8 (70.1–75.5)	72.7 (69.9–75.6)	74.3 (71.4–77.2)	74.2 (71.2–77.2)	73.5 (70.4–76.7)	73.3 (70.0–76.6)	76.1 (72.8–79.5)	77.0 (73.6–80.4)	79.4 (76.0–82.9)	NA^c^
**Sarcomas**												
Soft tissue sarcomas	78.9 (75.5–81.9)	84.8 (81.8–87.7)	91.1 (88.6–93.5)	94.1 (92.1–96.1)	94.8 (92.9–96.7)	95.9 (94.2–97.6)	96.5 (94.9–98.1)	96.8 (95.2–98.3)	96.7 (95.1–98.2)	98.2 (96.9–99.4)	98.1 (96.9–99.4)	5
Bone sarcomas	83.1 (79.3–86.3)	85.8 (82.5–89.1)	90.0 (87.1–92.8)	93.9 (91.6–96.2)	95.8 (93.8–97.7)	96.7 (94.9–98.5)	97.5 (96.0–99.1)	96.9 (95.1–98.7)	97.3 (95.5–99.1)	97.4 (95.6–99.2)	97.7 (95.9–99.5)	4
Other sarcomas	65.1 (55.8–72.9)	83.8 (76.2–91.4)	89.8 (83.3–96.3)	93.3 (87.8–98.7)	94.5 (89.6–99.5)	94.8 (90.2–99.5)	94.9 (90.2–99.6)	94.9 (90.2–99.6)	96.1 (91.9–100.3)	96.1 (91.8–100.3)	98.1 (94.6–101.5)	8
**Blood and lymphatic vessel tumors**	78.1 (70.4–84.1)	91.5 (86.5–96.5)	97.0 (93.8–100.2)	98.7 (96.4–101)	98.0 (95.3–100.7)	98.2 (95.6–100.8)	98.4 (95.9–100.8)	98.4 (96.0–100.8)	97.1 (94.0–100.2)	96.4 (93.0–99.8)	97.2 (94.0–100.3)	2
**Gonadal and related tumors**												
Testis, germ cell and trophoblastic	98.8 (98.4–99.1)	99.2 (98.9–99.5)	99.6 (99.3–99.8)	99.9 (99.7–100.1)	100.0 (99.8–100.2)	99.9 (99.7–100.1)	99.8 (99.5–100.0)	99.9 (99.7–100.1)	99.9 (99.7–100.2)	99.9 (99.6–100.1)	99.8 (99.5–100.1)	0
Ovary, germ cell and trophoblastic	97.0 (91.9–99.0)	98.6 (96.4–100.8)	99.3 (97.7–100.9)	100.2 (100.2–100.2)	100.2 (−)^f^	100.2 (−)^f^	100.2 (−)^f^	100.3 (100.3–100.3)	99.2 (97.1–101.3)	99.2 (97.1–101.4)	98.1 (95.0–101.2)	0
Ovary, non-germ cell	66.1 (60.8–70.8)	70.9 (65.8–76.0)	76.0 (70.9–81.1)	82.0 (77.2–86.9)	85.7 (81.0–90.4)	90.9 (86.8–94.9)	93.7 (90.1–97.2)	94.6 (91.3–98.0)	93.9 (90.4–97.4)	96.0 (93.0–98.9)	97.6 (95.4–99.9)	9
(Other) germ cell, non-germ cell and trophoblastic tumors	89.8 (84.3–93.4)	96.0 (92.8–99.1)	97.6 (95.0–100.2)	97.6 (95.1–100.2)	98.3 (96.1–100.6)	99.0 (97.2–100.8)	99.1 (97.2–100.9)	98.4 (96.1–100.7)	98.4 (96.1–100.7)	99.1 (97.2–101.0)	98.4 (96.1–100.8)	1
**Melanoma, malignant**	96.3 (95.7–96.8)	96.5 (96.0–97.0)	97.0 (96.5–97.4)	97.1 (96.6–97.5)	97.4 (97.0–97.9)	97.8 (97.4–98.2)	98.0 (97.6–98.4)	98.3 (97.9–98.7)	98.5 (98.1–98.9)	98.8 (98.4–99.1)	98.9 (98.6–99.3)	0
**Carcinomas**												
Thyroid carcinoma	99.4 (98.8–99.8)	99.6 (99.2–100.0)	99.8 (99.4–100.1)	99.7 (99.3–100.1)	99.7 (99.3–100.1)	99.7 (99.3–100.2)	99.8 (99.4–100.3)	99.7 (99.2–100.2)	99.9 (99.4–100.3)	99.8 (99.3–100.3)	99.9 (99.3–100.4)	0
Carcinoma of head and neck	86.8 (83.6–89.4)	90.7 (88.1–93.3)	93.7 (91.5–95.9)	95.5 (93.6–97.4)	96.8 (95.1–98.4)	96.4 (94.7–98.2)	96.2 (94.5–98.0)	95.9 (94.0–97.7)	95.9 (94.0–97.8)	96.4 (94.6–98.2)	95.7 (93.7–97.7)	3
Carcinoma of gastrointestinal tract												
Carcinoma of stomach	33.6 (27.9–39.4)	55.5 (47.4–63.5)	70.7 (62.0–79.3)	79.5 (71.3–87.6)	82.0 (74.1–89.9)	87.7 (80.6–94.7)	89.7 (83.0–96.4)	92.7 (86.6–98.8)	95.1 (89.8–100.4)	94.8 (89.2–100.4)	93.3 (87.0–99.7)	8^d^
Carcinoma of colon	67.3 (63.8–70.5)	75.9 (72.5–79.2)	84.0 (80.9–87.1)	89.3 (86.6–91.9)	92.9 (90.6–95.2)	94.7 (92.7–96.8)	96.9 (95.3–98.6)	97.2 (95.5–98.8)	97.5 (95.8–99.1)	97.7 (96.1–99.3)	98.4 (96.9–99.8)	6
Carcinoma of rectum	65.9 (61.2–70.2)	67.8 (63.2–72.4)	72.3 (67.7–76.9)	78.2 (73.8–82.7)	83.5 (79.4–87.6)	87.6 (83.9–91.4)	92.6 (89.5–95.8)	93.9 (90.9–96.8)	92.3 (89.0–95.6)	93.6 (90.4–96.7)	94.9 (91.9–97.8)	NA^c^
Carcinoma of liver and intrahepatic bile ducts (IBD)	39.9 (30.5–49.2)	67.4 (55.2–79.7)	79.5 (67.4–91.6)	81.2 (69.2–93.1)	92.0 (83.0–101.1)	94.7 (87.0–102.4)	97.5 (91.7–103.2)	100.5 (−)^b^	88.7 (73.4–104.1)	88.8 (73.4–104.1)	88.8 (73.5–104.2)	6^d^
Carcinoma of lung, bronchus, and trachea	26.4 (22.3–30.7)	40.7 (34.5–46.9)	52.8 (45.3–60.2)	61.8 (53.7–69.9)	67.5 (59.1–75.9)	77.9 (69.8–86.1)	84.8 (77.2–92.4)	85.6 (77.8–93.5)	87.4 (79.7–95.1)	90.9 (84.0–97.9)	87.4 (79.2–95.6)	NA^c^
Carcinoma of skin (if collected)^e^	97.5 (95.4–98.7)	97.9 (96.5–99.4)	98.3 (96.9–99.7)	97.9 (96.4–99.5)	98.5 (97.0–99.9)	98.5 (97.-100.0)	98.2 (96.6–99.8)	98.3 (96.6–99.9)	98.2 (96.5–99.9)	97.3 (95.2–99.3)	96.9 (94.6–99.1)	0
Carcinoma of breast	90.1 (89.4–90.8)	89.4 (88.6–90.1)	90.4 (89.7–91.2)	91.3 (90.6–92.0)	92.4 (91.7–93.1)	93.0 (92.3–93.7)	93.7 (93.0–94.4)	94.0 (93.3–94.6)	94.4 (93.7–95.0)	94.4 (93.7–95.0)	94.3 (93.6–95.0)	NA^c^
Carcinoma of genital sites excluding ovary and testis												
Carcinoma of uterine cervix	90.6 (89.3–91.7)	92.1 (90.9–93.2)	94.6 (93.6–95.6)	96.1 (95.2–97.0)	97.1 (96.3–97.9)	97.5 (96.7–98.3)	97.5 (96.7–98.3)	98.1 (97.3–98.8)	98.4 (97.6–99.1)	98.3 (97.6–99.1)	98.5 (97.8–99.2)	3
Carcinoma of corpus uteri	91.6 (85.5–95.3)	94.7 (90.2–99.1)	96.9 (93.1–100.7)	96.9 (93.2–100.7)	95.8 (91.4–100.2)	96.9 (92.9–100.8)	99.2 (96.8–101.7)	96.5 (92.1–101.0)	96.6 (92.2–101.0)	97.9 (94.1–101.7)	98.0 (94.2–101.8)	2
Carcinoma of vulva and vagina	92.0 (85.5–95.7)	92.7 (88.0–97.5)	96.8 (93.3–100.2)	97.6 (94.6–100.7)	98.5 (96.0–101.1)	98.3 (95.5–101.2)	98.1 (94.9–101.3)	98.2 (95.0–101.4)	97.0 (92.9–101)	97.0 (93.0–101.1)	98.3 (94.8–101.7)	2
Carcinoma of urinary tract												
Carcinoma of kidney	89.2 (85.8–91.9)	94.6 (92.3–96.9)	96.0 (93.9–98.1)	96.7 (94.7–98.7)	97.4 (95.5–99.3)	96.8 (94.7–98.9)	97.1 (95.1–99.1)	96.7 (94.5–98.9)	96.6 (94.3–98.9)	96.2 (93.7–98.8)	96.9 (94.4–99.3)	2
Carcinoma of bladder	56.2 (47.1–64.3)	71.5 (62.3–80.7)	79.5 (70.7–88.4)	84.5 (76.3–92.8)	86.7 (78.8–94.6)	92.0 (85.5–98.5)	93.7 (87.9–99.5)	94.0 (88.3–99.6)	95.4 (90.3–100.5)	98.2 (94.7–101.7)	98.3 (94.7–101.8)	8
Other invasive carcinomas	39.3 (32.4–46.2)	70.6 (61.6–79.6)	74.5 (65.4–83.6)	77.3 (68.3–86.4)	81.5 (72.9–90.1)	85.0 (77.0–93.1)	90.2 (83.4–97.0)	90.6 (84.1–97.2)	93.2 (87.4–98.9)	89.7 (82.9–96.5)	88.6 (81.6–95.7)	NA^c^
Neuroendocrine tumors (NET)	96.7 (95.1–97.8)	98.1 (97.0–99.2)	98.9 (98.0–99.8)	99.3 (98.5–100.1)	99.5 (98.6–100.3)	99.7 (98.9–100.5)	99.7 (98.9–100.5)	99.0 (97.8–100.3)	99.3 (98.1–100.5)	99.9 (98.9–100.9)	98.9 (97.3–100.4)	0
Miscellaneous specified neoplasms	65.1 (53.7–74.4)	73.0 (61.9–84.1)	79.5 (68.5–90.5)	87.1 (77.3–97.0)	95.3 (88.5–102.1)	95.1 (87.9–102.2)	97.6 (92.3–103.0)	97.7 (92.4–103.0)	97.7 (92.4–103.1)	94.8 (86.9–102.6)	94.7 (86.8–102.7)	4

CRS estimates were only generated for cancer types with a number at risk of more than n = 100 at the time of diagnosis.

Abbreviations: CRS = Conditional Relative Survival, CI = Confidence Intervals, PE = Point-estimate, CNS = Central Nervous System, NA = Not Applicable.

a Estimates for the CRS for 0 years survived correspond with the five-year relative survival at the time of diagnosis.

b Cancer types were categorized into diagnostic groups according to the revised morphology based AYA-specific classification scheme developed by Barr et al. (2021). Cancer types were color coded into three groups based on the five-year CRS estimates: CRS > 95% from diagnosis or within five years post-diagnosis (green), CRS > 95% between five and ten years post-diagnosis (orange), and CRS < 95% in ten years post-diagnosis (red).

c NA was indicated whenever the CRS point-estimate did not surpass the 95% minimal excess mortality threshold in ten years post-diagnosis.

d Passes the threshold in just one or a few non-consecutive years.

e The Netherlands Cancer Registry does not collect data about basal-cell skin and lip carcinomas.

f 95% CI could not be calculated due to standard error calculation resulting in negative root numbers.

### Conditional relative survival by age group

**Fig. 2:**
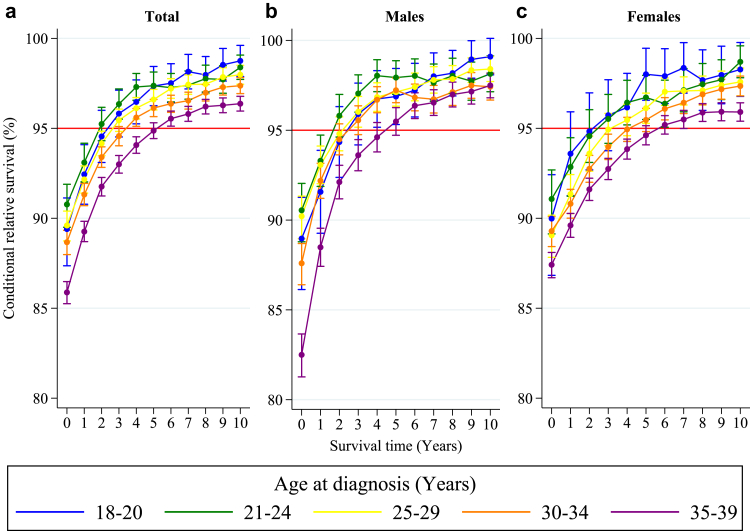
**Five-year conditional relative survival by age group.** Five-year conditional relative survival (CRS) per age group up to 10 years post-diagnosis of adolescents and young adults (AYAs, aged 18–39 years) diagnosed with an invasive solid malignancy in the Netherlands between 1998 and 2021 for the entire cohort (a), males (b), and females (c). CRS estimates were calculated using the hybrid approach for all types of solid malignancies combined. The error bars indicate the 95% confidence interval. The red line indicates the 95% minimal excess mortality threshold.

At the time of diagnosis, the five-year CRS varied by age, ranging from 85.9% (95% CI: 85.3–86.5) for ages 35–39 years to 90.8% (95% CI: 89.5–91.9) for ages 21–24 years. Older AYAs tended to reach the 95% threshold later than those diagnosed at a younger age. AYAs aged 18–20 years at diagnosis reached the 95% threshold after three years. Those aged 21–24 exceeded the threshold after two years, and those aged 25–29 after three years. Patients aged 30–34 and 35–39 years reached the threshold after four and six years, respectively (Fig. 2 and Table 2). CRS estimates stratified by sex are available in Appendix Tables 3 and 4. Among male AYA solid malignancy survivors, all age groups reached the 95% threshold after three years of survival, except for those aged 21–24 and 35–39 years at diagnosis, who achieved this threshold after two and five years, respectively (Fig. 2 and Appendix Table 3). Among female AYA solid malignancy survivors, those aged 18–20 and 21–24 years at diagnosis reached the 95% threshold after a survival period of three years, those aged 25–29 years after four years, those aged 30–34 years after five years, and patients aged 35–39 years at diagnosis reached this threshold after six years (Fig. 2 and Appendix Table 4).

### Conditional relative survival by diagnostic group

#### CRS > 95% from diagnosis or within five years post-diagnosis

**Fig. 3:**
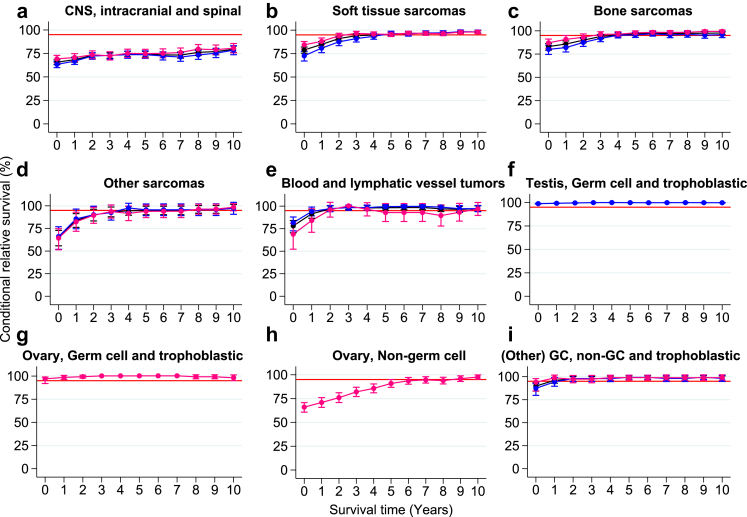

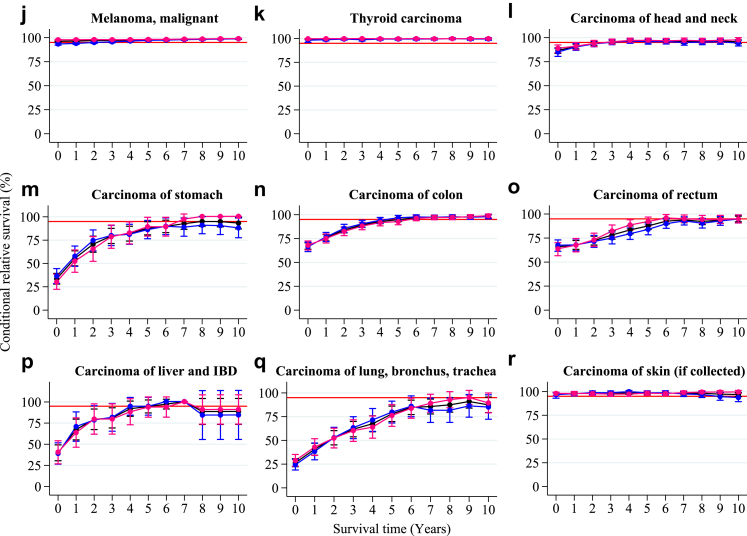

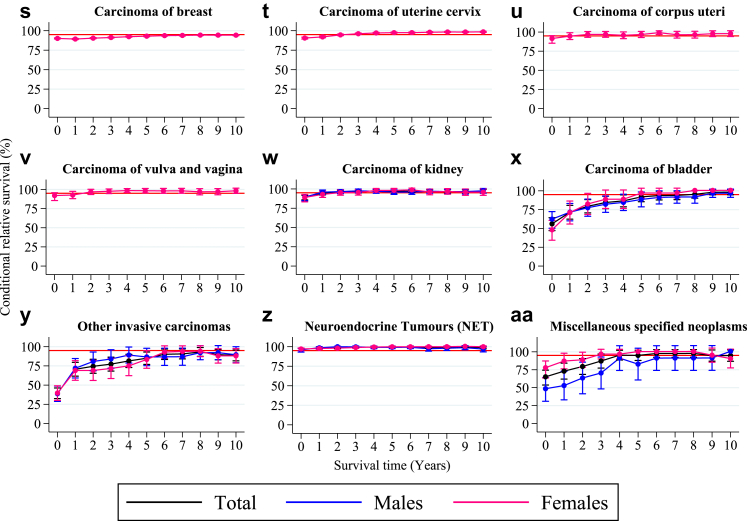
**Five-year conditional relative survival by diagnostic group.** Five-year conditional relative survival (CRS) per diagnostic group up to 10 years post-diagnosis of adolescents and young adults (AYAs, aged 18–39 years) diagnosed with an invasive solid malignancy in the Netherlands between 1998 and 2021 for the entire cohort (black), males (blue), and females (pink). CRS estimates were calculated using the hybrid approach. The error bars indicate the 95% confidence interval. The red line indicates the 95% minimal excess mortality threshold. CRS estimates were only generated for cancer types with a number at risk of more than n = 100 at the time of diagnosis. Abbreviations: CNS = central nervous system; GC = germ cell; IBD = intrahepatic bile ducts.

AYA survivors of germ cell and trophoblastic tumors (ovarian and testis), malignant melanoma, thyroid carcinoma, skin carcinoma, and neuroendocrine tumors (NET) exhibited a five-year CRS greater than 95% from diagnosis. AYA survivors of soft tissue sarcomas, bone sarcomas, blood and lymphatic vessel tumors, other germ cell, non-germ cell and trophoblastic tumors, carcinoma of the head and neck, carcinoma of the uterine cervix, carcinoma of the corpus uteri, carcinoma of the vulva and vagina, carcinoma of the kidney, and miscellaneous specified neoplasm reached the 95% threshold in five years post-diagnosis (Fig. 3 and Table 2).

#### CRS > 95% between five and 10 years post-diagnosis

AYA survivors of other sarcomas, ovary non germ-cell tumors, and carcinomas of the stomach, colon, liver and intrahepatic bile ducts (IBD) and bladder reached the 95% threshold between five and ten years post-diagnosis (Fig. 3 and Table 2).

#### CRS < 95% in 10 years post-diagnosis

AYA solid malignancy survivors of CNS and other intracranial and intraspinal neoplasms, and carcinomas of the rectum, lung, bronchus, and trachea, breast and other invasive carcinomas did not reach the 95% CRS threshold within 10 years (Fig. 3 and Table 2). CRS estimates stratified by diagnostic group and sex can be found in Appendix Tables 3 and 4.

## Discussion

This study investigated the five-year CRS of AYA solid malignancy survivors to determine whether there was evidence to support relaxing the current required period to be forgotten for this population in the Netherlands, which can have important implications for other European countries with RTBF policies. Findings demonstrated that survival outcomes gradually improved with additional years survived in most cases, whereby the minimal excess mortality threshold (CRS > 95%) was reached by the entire AYA solid malignancy survivor cohort after a survival period of four years. For AYA survivors of most solid malignancy types, this 95% threshold was reached within five or ten years post-diagnosis. However, this was not the case for survivors of CNS and other intracranial and intraspinal neoplasms, other invasive carcinomas, and carcinomas of the rectum, lung and breast. In these groups, the 95% threshold was not reached within the investigated ten-year survival period, highlighting that there are important differences in survival and post-diagnostic improvements among AYAs.

Anderson et al. (2018) is one of the few studies examining the CRS among AYA cancer patients with data from The Surveillance, Epidemiology, and End Results (SEER) database.^24^ The study included AYAs diagnosed between 1973 and 2009, with follow-up extending through 2014, and calculated five-year CRS estimates up to 25 years post-diagnosis. For all cancers combined, the five-year CRS at one-year post-diagnosis was estimated at 80.3% (CI: 80.0–80.7) for the cohort diagnosed between 1973 and 1987 and increased to 95.9% (CI: 95.7–96.1) after a survival period of ten years. For the cohort diagnosed between 1988 and 2009, the five-year CRS increased from 86.6% (CI: 86.4–86.8) at one year post-diagnosis to 97.0% (CI: 96.8–97.1) after having survived ten years. The five-year CRS exceeded the threshold of minimal excess mortality (CRS > 95%) at eight years post-diagnosis for the cohort diagnosed between 1973 and 1987, and six years post-diagnosis for the cohort diagnosed between 1988 and 2009. Additionally, Anderson et al. found that patients with thyroid tumors had a five-year CRS of more than 95% from diagnosis, while those with female breast tumors, leukemias, and CNS tumors did not reach the 95% threshold within ten years from diagnosis. The five-year CRS estimates found in the current study are more optimistic, which can be attributed to the use of a hybrid approach and the availability of recent data (until 2023).^35^ The hybrid approach is known to yield higher estimates than the cohort approach when survival probabilities are increasing, which is the case in Europe.^19^^,^^35^ Furthermore, Anderson et al. also included non-solid malignancies diagnosed at AYA age, whereby they showed that the 95% threshold for AYA lymphoma survivors (i.e., Hodgkin and non-Hodgkin) was reached within ten years post-diagnosis, whereas this was 13 years for AYA leukemia survivors.^24^ This far exceeds the four-year period that was observed for the entire AYA solid malignancy survivor cohort in our study, which now likely overestimates outcomes for all AYA survivors. More importantly, considering the more optimistic nature of our outcomes, current RTBF laws in the Netherlands likely also disadvantage AYA non-solid malignancy survivors. Considering that this tumor group was not included in the Dutch clean-slate policy until late 2024, to optimize future policy discussions, we recommend future studies that comprehensively investigates the AYA non-solid malignancy population.^38^

The use of CRS estimates provides valuable insights into identifying distinct risk groups based on long-term survival trends. For most solid malignancy types in the current AYA cohort, the threshold of minimal excess mortality (CRS > 95%) was observed from diagnosis or within five years post-diagnosis compared to the general population, well before the ten-year required period to be forgotten by the RTBF laws in most countries.^11^ This suggests that the required period to be forgotten could potentially be reduced to five years for AYAs with those types of tumors. Currently, the period to be forgotten is already seven years in Romania, while Spain and France have reduced it even further to five years. As of January 2025, the period to be forgotten will also be five years in Belgium.^6^^,^^7^^,^^11^^,^^39^ Furthermore, for AYA survivors with cancer types that initially have a poor prognosis, but exhibit minimal excess mortality after five years and within ten years from diagnosis, the required disclosure period could likely be shortened based on a personalized risk assessment. This may reduce financial toxicity in AYA cancer patients, thereby enabling them to reintegrate and actively contribute to society sooner. Additionally, discussing these findings during consultations can significantly enhance decision-making and expectation management regarding follow-up care. It may help AYA solid malignancy survivors understand that cancer survival is a conditional event and that their probability of survival changes with every additional year they survive.

The situation remains more challenging for AYAs with solid malignancy types that maintain a poor cancer prognosis up to ten years post-diagnosis (CRS < 95%). These AYA survivors represent a diverse minority with unique care needs, which may necessitate special and tailored policies to protect them from financial toxicity.^40^^,^^41^ From previous literature, it is known that AYAs with a poor prognosis often want to choose life over illness, trying to minimize the impact of cancer.^42^ However, current financial policies may hinder their ability to lead normal lives. Therefore, it is important to develop supportive strategies to help these patients resume normal life in all aspects, which includes applying for insurance and obtaining mortgages. Additionally, conducting cancer type-specific analyses within this group could allow for more tailored approaches to address the unique needs of each subgroup, incorporating the variations existing within a cancer type.

As the findings of this study can be utilized by different stakeholders, the intention must always be to provide appropriate care for every patient. For those with a good prognosis, this means facilitating their reintegration into society as smoothly as possible. For those with a less favorable prognosis, it involves providing the right level of support to meet their specific challenges. Moreover, with the advancements in cancer treatment, constant re-evaluation of survival estimates is essential, as it is expected that more patients will have a favorable prognosis over time and to ensure that these young patients, for whom integration into society is of high importance, are not disadvantaged.^19^

A major strength of the current study is the fact that this is the first study examining five-year CRS among AYA cancer patients in a European country, with nearly 100% coverage. CRS estimates provide clinically relevant long-term information for patients, healthcare providers, and policymakers by accounting for the number of years individuals have already survived, making it a more pragmatic measure than standard unconditional relative survival estimates.^25^, ^26^, ^27^, ^28^ Due to the increasing number of AYA cancer survivors in Europe, their integration into society is becoming increasingly important, and research in this area is crucial. Additionally, AYA cancer survivors can live more than 50 years after their initial cancer diagnosis and have a long life ahead compared to older cancer patients, making long-term survival outcomes and RTBF laws even more crucial for them than for others.^43^ Another strength is the large sample size (n = 71,973) and the availability of high-quality oncological data from the NCR, which allows for precise survival estimates, even for rare primary solid malignancies, and limits selection bias. Moreover, the hybrid approach, which combines a cohort approach with a period approach, was utilized to calculate the CRS. The hybrid approach provides more up-to-date and accurate cancer survival estimates compared to other approaches, such as the traditional cohort approach used in previous research.^24^^,^^35^

An important limitation of the current study is the absence of data on full remission, recurrence, and distant metastases, which are important for defining the cancer-free period of RTBF laws. The exclusion of AYAs diagnosed with multiple solid malignancies likely resulted in outcomes that are more selective and optimistic, as subsequent malignancies tend to have worse prognostic outcomes.^44^^,^^45^ Still, outcomes are expected to be minimally affected, as comparable relative survival outcomes were observed when AYAs with multiple malignancies were included (data not shown). Information about (long-term and late) survivorship-related health issues was also not available, which prevented more stratified analyses that could have added to personalized survival risk profiles to become more clearly defined. Another limitation is the exclusion of hematological malignancies, which to some extent impacts the generalizability of outcomes to the entire AYA survivor population. Still, this study is among the largest to date in terms of included (solid) cancer types, making our findings generalizable to the majority of AYA survivors. Nevertheless, future research should aim to include these elements. Furthermore, the current study did not stratify the five-year CRS estimates on patient demographics, tumor characteristics, or treatment regimen per cancer type. Additional tumor-specific survival research should be conducted to fully comprehend survival per cancer type and the implications of policy adjustments.

### Conclusion

The entire AYA solid malignancy survivor cohort reached the threshold of minimal excess mortality (CRS > 95%) after a survival period of four years, well before the required period to be forgotten by the RTBF laws in most European countries, including the Netherlands. Moreover, the impact of cancer on survival decreased significantly with additional years survived. For AYA survivors of germ cell and trophoblastic tumors (ovarian and testis), malignant melanomas, thyroid carcinomas, skin carcinomas, and NET, the excess mortality was minimal from the moment of diagnosis, indicating that the survival of AYAs diagnosed with any of these tumors is comparable to that of the general population. Most other solid malignancy types reached the 95% threshold within five or 10 years post-diagnosis. This might be a first indication that the RTBF laws should be re-evaluated for AYA survivors of solid cancers. It is important to frequently re-evaluate the five-year CRS of AYA cancer patients, given the development and improvement of novel treatments, ensuring appropriate support and policies to help them reintegrate successfully into society as soon as possible.

## Contributors

Conceptualization: all authors; data curation: HK, WVDG, OH; formal analysis: DVDM, BW, HK; funding acquisition: WVDG, OH; investigation: NVP, DVDM, BW, HK; methodology: NVP, DVDM, BW, HK; project administration: WVDG, OH; resources: HK, WVDG, OH; software: DVDM, HK; supervision: HK, WVDG, OH; validation: DVDM, HK; visualization: NVP, DVDM, HK; writing – original draft: NVP, HK; writing – review & editing: all authors.

All authors had full access to the data in the study and take final responsibility for the decision to submit the manuscript for publication.

## Data sharing statement

Data used in this study can be requested from the NCR (study number K23.261, www.iknl.nl).

## Declaration of interests

The authors have no competing interest to declare

## Appendix A Supplementary data

Appendix Figure and Tables

STROBE Checklist
