# Supplementary material for: 5-Year conditional relative survival of adolescents and young adults with solid malignancies in the Netherlands: a population-based cohort study
Source: Lancet Reg Health Eur. 2025 Aug 13;57:101429. doi: 10.1016/j.lanepe.2025.101429 (PMC12541644; doi:10.1016/j.lanepe.2025.101429)
Supplement: Appendix Figure and Tables [file mmc1.docx]

**Supplementary materials:**

**5-year conditional relative survival of adolescents and young adults with solid malignancies in the Netherlands: a population-based cohort study**

Noelle J.M.C. Vrancken Peeters, Daniël J. van der Meer, Bo Wardenier, Marion L'hôte, Eveliene Manten-Horst, Henrike E. Karim-Kos, Winette T. A. van der Graaf and Olga Husson.

**Table of contents**

[Supplementary Table 1. Population and tumor characteristics of adolescents and young adults (AYAs, aged 18-39 years) diagnosed with one or multiple invasive solid malignancies in the Netherlands between 1998-2021. 2](#_Toc204159659)

[Supplementary Table 2. Number at risk and relative survival estimates with 95% confidence intervals and standard errors up to 15 years post-diagnosis of adolescents and young adults (AYAs, aged 18-39 years) diagnosed with an invasive solid malignancy in the Netherlands between 1998-2021 by sex, age at diagnosis and cancer type. Only groups with a minimum of n=100 cases at risk were included in subsequent conditional relative survival analyses. 3](#_Toc204159660)

[Supplementary Figure 1. Structure of the follow-up data using the hybrid approach. The numbers within the cells represent the minimum years of follow-up (column) since diagnosis (row). For the calculation of five-year relative survival at diagnosis, as well as each additional year survived up to 10 years post-diagnosis (i.e., conditional five-year relative survival), the data in the grey-shaded areas were used. 10](#_Toc204159661)

[Supplementary Table 3. Five-year conditional relative survival with 95% confidence intervals up to 10 years post-diagnosis of male adolescents and young adults (AYAs, aged 18-39 years) diagnosed with an invasive solid malignancy in the Netherlands between 1998-2021. CRS estimates were only generated for cancer types with a number at risk of more than n=100 at the time of diagnosis. 11](#_Toc204159662)

[Supplementary Table 4. Five-year conditional relative survival with 95% confidence intervals up to 10 years post-diagnosis of female adolescents and young adults (AYAs, aged 18-39 years) diagnosed with an invasive solid malignancy in the Netherlands between 1998-2021. CRS estimates were only generated for cancer types with a number at risk of more than n=100 at the time of diagnosis. 12](#_Toc204159663)

# Supplementary Table 1. Population and tumor characteristics of adolescents and young adults (AYAs, aged 18-39 years) diagnosed with one or multiple invasive solid malignancies in the Netherlands between 1998-2021.

|  | **Both**  **n=(%)** | | | **Males**  **n=(%)** | | | **Females**  **n=(%)** | | |
| --- | --- | --- | --- | --- | --- | --- | --- | --- | --- |
| **Number of diagnoses** | **n=1** | **n>1** | **p-value*** | **n=1** | **n>1** | **p-value*** | **n=1** | **n>1** | **p-value*** |
| **Total**** | 71,973 (100.0) | 4,321 (100.0) |  | 27,284 (100.0) | 1,608 (100.0) |  | 44,689 (100.0) | 2,713 (100.0) |  |
| **Median age at diagnosis (IQR)***** | 34.0 (29.0-37.0) | 34.0 (29.0-37.0) | 0.368 | 32.0 (27.0-36.0) | 32.0 (27.0-36.0) | 0.666 | 34.0 (30.0-37.0) | 34.0 (30.0-37.0) | 0.340 |
| **Age at diagnosis (Years)** |  |  | 0.000 |  |  | 0.104 |  |  | 0.001 |
| 18-20 | 2,413 (3.4) | 96 (2.2) |  | 1,392 (5.1) | 60 (3.7) |  | 1,021 (2.3) | 36 (1.3) |  |
| 21-24 | 5,052 (7.0) | 290 (6.7) |  | 2,807 (10.3) | 162 (10.0) |  | 2,245 (5.0) | 128 (4.7) |  |
| 25-29 | 11,602 (16.1) | 733 (17.0) |  | 5,436 (19.9) | 333 (21.0) |  | 6,166 (13.8) | 400 (15.0) |  |
| 30-34 | 20,586 (28.6) | 1,316 (30.5) |  | 7,562 (27.7) | 470 (29.2) |  | 13,024 (29.1) | 846 (31.2) |  |
| 35-39 | 32,320 (44.9) | 1,886 (43.7) |  | 10,087 (37.0) | 583 (36.3) |  | 22,233 (49.8) | 1,303 (48.0) |  |
| **Median Survival time (IQR)***** | 8.9 (3.6-15.9) | 9.0 (3.7-15.6) | 0.907 | 8.6 (3.3-15.6) | 9.8 (4.1-15.8) | 0.000 | 9.1 (3.8-16.1) | 8.5 (3.5-15.4) | 0.004 |
| **Period of diagnosis (Years)** |  |  | 0.000 |  |  | 0.003 |  |  | 0.000 |
| 1998-2005 | 23,245 (32.3) | 1,423 (32.9) |  | 8,565 (31.4) | 506 (31.5) |  | 14,680 (32.9) | 917 (33.8) |  |
| 2006-2013 | 23,742 (33.0) | 1,564 (36.2) |  | 9,195 (33.7) | 600 (37.3) |  | 14,547 (32.6) | 964 (35.5) |  |
| 2014-2021 | 24,986 (34.7) | 1,334 (30.9) |  | 9,524 (34.9) | 502 (31.2) |  | 15,462 (34.6) | 832 (30.7) |  |
| **Diagnostic group****** |  |  | 0.000 |  |  | 0.000 |  |  | 0.000 |
| **CNS and other intracranial and intraspinal neoplasms** | 3,459 (4.8) | 94 (2.2) |  | 2,035 (7.5) | 50 (3.1) |  | 1,424 (3.2) | 44 (1.6) |  |
| **Sarcomas** | 3343 (4.6) | 145 (3.4) |  | 1712 (6.3) | 72 (4.5) |  | 1631 (3.7) | 73 (2.7) |  |
| Soft tissue sarcomas | 1,785 (2.5) | 55 (1.3) |  | 870 (3.2) | 28 (1.7) |  | 915 (2.1) | 27 (1.0) |  |
| Bone sarcomas | 1,078 (1.5) | 67 (1.6) |  | 603 (2.2) | 33 (2.1) |  | 475 (1.1) | 34 (1.3) |  |
| Gastrointestinal stromal tumors (GIST) | 156 (0.2) | 4 (0.1) |  | 87 (0.3) | 1 (0.1) |  | 69 (0.2) | 3 (0.1) |  |
| Other sarcomas | 324 (0.5) | 19 (0.4) |  | 152 (0.6) | 10 (0.6) |  | 172 (0.4) | 9 (0.3) |  |
| **Blood and lymphatic vessel tumors** | 439 (0.6) | 17 (0.4) |  | 327 (1.2) | 11 (0.7) |  | 112 (0.3) | 6 (0.2) |  |
| **Nerve sheath tumors** | 188 (0.3) | 12 (0.3) |  | 98 (0.4) | 2 (0.1) |  | 90 (0.2) | 10 (0.4) |  |
| **Gonadal and related tumors** | 12,650 (17.6) | 800 (18.5) |  | 11,109 (40.7) | 732 (45.5) |  | 1,541 (3.5) | 68 (2.5) |  |
| Testis, Germ cell and trophoblastic | 10,792 (15.0) | 717 (16.6) |  | 10,792 (39.6) | 717 (44.6) |  | NA | 0 (0.0) |  |
| Testis, Non-germ cell | 41 (0.1) | 1 (0.0) |  | 41 (0.2) | 1 (0.1) |  | NA | 0 (0.0) |  |
| Ovary, Germ cell and trophoblastic | 270 (0.4) | 8 (0.2) |  | NA | 0 (0.0) |  | 270 (0.6) | 8 (0.3) |  |
| Ovary, Non-germ cell | 1,102 (1.5) | 58 (1.3) |  | NA | 0 (0.0) |  | 1,102 (2.5) | 58 (2.1) |  |
| (Other) Germ cell, non-germ cell and trophoblastic tumors | 445 (0.6) | 16 (0.4) |  | 276 (1.0) | 14 (0.9) |  | 169 (0.4) | 2 (0.1) |  |
| **Melanoma, malignant** | 14,137 (19.6) | 1,156 (26.8) |  | 4,947 (18.1) | 325 (20.2) |  | 9,190 (20.6) | 831 (30.6) |  |
| **Carcinomas** | 37,370 (51.9) | 2,057 (47.6) |  | 6,851 (25.1) | 399 (24.8) |  | 30,519 (68.3) | 1,658 (61.1) |  |
| Thyroid carcinoma | 3,183 (4.4) | 97 (2.2) |  | 742 (2.7) | 31 (1.9) |  | 2,441 (5.5) | 66 (2.4) |  |
| Carcinoma of head and neck | 1,450 (2.0 | 81 (1.9) |  | 828 (3.0) | 36 (2.2) |  | 622 (1.4) | 45 (1.7) |  |
| Carcinoma of gastrointestinal tract | 5729 (8.0) | 325 (7.6) |  | 2881 (10.6) | 182 (11.4) |  | 2848 (6.4) | 143 (5.3) |  |
| Carcinoma of esophagus | 236 (0.3) | 5 (0.1) |  | 173 (0.6) | 2 (0.1) |  | 63 (0.1) | 3 (0.1) |  |
| Carcinoma of stomach | 707 (1.0) | 26 (0.6) |  | 376 (1.4) | 13 (0.8) |  | 331 (0.7) | 13 (0.5) |  |
| Carcinoma of small intestine | 77 (0.1) | 11 (0.3) |  | 40 (0.2) | 5 (0.3) |  | 37 (0.1) | 6 (0.2) |  |
| Carcinoma of colon | 1,821 (2.5) | 133 (3.1) |  | 879 (3.2) | 74 (4.6) |  | 942 (2.1) | 59 (2.2) |  |
| Carcinoma of rectum | 1,079 (1.5) | 65 (1.5) |  | 583 (2.1) | 40 (2.5) |  | 496 (1.1) | 25 (0.9) |  |
| Carcinoma of anus | 120 (0.2) | 6 (0.1) |  | 62 (0.2) | 3 (0.2) |  | 58 (0.1) | 3 (0.1) |  |
| Carcinoma of liver and intrahepatic bile ducts (IBD) | 225 (0.3) | 13 (0.3) |  | 126 (0.5) | 9 (0.6) |  | 99 (0.2) | 4 (0.2) |  |
| Carcinoma of gallbladder and other extrahepatic biliary | 166 (0.2) | 9 (0.2) |  | 96 (0.4) | 4 (0.3) |  | 70 (0.2) | 5 (0.2) |  |
| Carcinoma of pancreas | 275 (0.4) | 11 (0.3) |  | 133 (0.5) | 7 (0.4) |  | 142 (0.3) | 4 (0.2) |  |
| Other carcinoma of gastrointestinal tract | 18 (0.0) | 3 (0.1) |  | 11 (0.0) | 3 (0.2) |  | 7 (0.0) | 0 (0.0) |  |
| Carcinoma of lung, bronchus, and trachea | 1,293 (1.8) | 55 (1.3) |  | 634 (2.3) | 13 (0.8) |  | 659 (1.5) | 42 (1.6) |  |
| Carcinoma of skin (if collected)***** | 949 (1.3) | 58 (1.3) |  | 425 (1.6) | 23 (1.4) |  | 524 (1.2) | 35 (1.3) |  |
| Carcinoma of breast | 16,501 (22.9) | 1,092 (25.3) |  | 37 (0.1) | 1 (0.1) |  | 16,464 (36.8) | 1,091 (40.2) |  |
| Carcinoma of genital sites excluding ovary and testis | 5899 (8.2) | 134 (3.9) |  | 69 (0.3) | 10 (0.6) |  | 5830 (13.1) | 158 (5.9) |  |
| Carcinoma of uterine cervix | 5,246 (7.3) | 103 (2.4) |  | NA | 0 (0.0) |  | 5,246 (11.7) | 103 (3.8) |  |
| Carcinoma of corpus uteri | 288 (0.4) | 31 (0.7) |  | NA | 0 (0.0) |  | 288 (0.6) | 31 (1.1) |  |
| Carcinoma of vulva and vagina | 279 (0.4) | 22 (0.5) |  | NA | 0 (0.0) |  | 279 (0.6) | 22 (0.8) |  |
| Carcinoma of penis | 49 (0.1) | 2 (0.1) |  | 49 (0.2) | 2 (0.1) |  | NA | 0 (0.0) |  |
| Carcinoma of prostate | 17 (0.0) | 6 (0.1) |  | 17 (0.1) | 6 (0.4) |  | NA | 0 (0.0) |  |
| Other genital | 20 (0.0) | 4 (0.1) |  | 3 (0.0) | 2 (0.1) |  | 17 (0.0) | 2 (0.1) |  |
| Carcinoma of urinary tract | 1294 (1.8) | 134 (3.1) |  | 767 (2.8) | 87 (5.4) |  | 527 (1.2) | 47 (1.7) |  |
| Carcinoma of kidney | 917 (1.3) | 109 (2.5) |  | 552 (2.0) | 71 (4.4) |  | 365 (0.8) | 38 (1.4) |  |
| Carcinoma of bladder | 347 (0.5) | 19 (0.4) |  | 196 (0.7) | 12 (0.8) |  | 151 (0.3) | 7 (0.3) |  |
| Other urinary | 30 (0.0) | 6 (0.1) |  | 19 (0.1) | 4 (0.3) |  | 11 (0.0) | 2 (0.1) |  |
| Other invasive carcinomas | 736 (1.0) | 33 (0.8) |  | 335 (1.2) | 11 (0.7) |  | 401 (0.9) | 22 (0.8) |  |
| Neuroendocrine Tumors (NET) | 1,275 (1.8) | 51 (1.2) |  | 502 (1.8) | 24 (1.5) |  | 773 (1.7) | 27 (1.0) |  |
| Neuroendocrine carcinomas (NEC) | 66 (0.1) | 6 (0.1) |  | 33 (0.1) | 3 (0.2) |  | 33 (0.1) | 3 (0.1) |  |
| Miscellaneous specified neoplasms | 213 (0.3) | 13 (0.3) |  | 92 (0.3) | 8 (0.5) |  | 121 (0.3) | 5 (0.2) |  |
| Unspecified malignant neoplasms except CNS | 174 (0.2) | 27 (0.6) |  | 113 (0.4) | 9 (0.6) |  | 61 (0.1) | 18 (0.7) |  |
| **Tumor stage (TNM & FIGO)******** |  |  | 0.000 |  |  | 0.000 |  |  | 0.000 |
| stage I | 35,532 (49.4) | 2,526 (58.5) |  | 13,987 (51.3) | 1,035 (64.4) |  | 21,545 (48.0) | 1,491 (55.0) |  |
| stage II | 14,581 (20.3) | 739 (17.1) |  | 3,565 (13.1) | 173 (10.8) |  | 11,016 (25.0) | 566 (20.9) |  |
| stage III | 8,419 (11.7) | 452 (10.5) |  | 3,331 (12.2) | 160 (10.0) |  | 5,088 (11.4) | 292 (10.8) |  |
| stage IV | 5,011 (7.0) | 252 (5.8) |  | 2,126 (7.8) | 73 (4.5) |  | 2,885 (6.5) | 179 (6.6) |  |
| Other/Missing | 8,430 (11.7) | 352 (8.2) |  | 4,275 (15.7) | 167 (10.4) |  | 4,155 (9.3) | 185 (6.8) |  |

Abbreviation: IQR=Interquartile range, CNS=Central Nervous System, TNM=Tumor Node Metastasis, Figo=Fédération Internationale de Gynécologie et d'Obstétrique, NA=Not Applicable.

* P-values of differences between AYAs diagnosed with one versus multiple invasive solid malignancies. Pearson's X2 tests were used for categorical variables and Kruskal-Wallis tests for continuous variables.

** Percentages might not add-up to 100% due to rounding.

*** The Kruskal-Wallis tests for continuous variables was used on the underlying continuous data.

**** Cancer types were categorized into diagnostic groups according to the revised morphology based AYA-specific classification scheme developed by Barr et al. (2021).

***** The Netherlands Cancer Registry does not collect data about basal-cell skin and lip carcinomas.

****** Staging of solid malignancies was done according to the Union for International Cancer Control (UICC) TNM-classification of malignant tumours, varying from the fourth edition between 1989-1998 to the eighth edition since 2017. Gynecological tumors were staged based on the FIGO staging system, which is incorporated in all editions of the TNM-classification.

# Supplementary Table 2. Number at risk and relative survival estimates with 95% confidence intervals and standard errors up to 15 years post-diagnosis of adolescents and young adults (AYAs, aged 18-39 years) diagnosed with an invasive solid malignancy in the Netherlands between 1998-2021 by sex, age at diagnosis and cancer type. Only groups with a minimum of n=100 cases at risk were included in subsequent conditional relative survival analyses.

|  |  | **Both** | | | **Males** | | | **Females** | | |
| --- | --- | --- | --- | --- | --- | --- | --- | --- | --- | --- |
|  | **Survival time (Years)** | **Number at risk (n=)** | **RS (95% CI)*** | **SE** | **Number at risk (n=)** | **RS (95% CI)*** | **SE** | **Number at risk (n=)** | **RS (95% CI)*** | **SE** |
| **Total** |  |  |  |  |  |  |  |  |  |  |
|  | 1 | 30,798 | 95.7 (95.4-95.9) | 0.1 | 11,848 | 94.4 (93.9-94.8) | 0.2 | 18,950 | 96.5 (96.2-96.8) | 0.1 |
|  | 2 | 32,025 | 92.5 (92.2-92.8) | 0.2 | 12,161 | 91.0 (90.5-91.5) | 0.3 | 19,864 | 93.5 (93.1-93.8) | 0.2 |
|  | 3 | 30,307 | 90.6 (90.3-90.9) | 0.2 | 11,514 | 89.2 (88.7-89.8) | 0.3 | 18,793 | 91.5 (91.1-91.9) | 0.2 |
|  | 4 | 29,305 | 89.1 (88.7-89.4) | 0.2 | 11,143 | 87.8 (87.2-88.4) | 0.3 | 18,162 | 89.9 (89.4-90.3) | 0.2 |
|  | 5 | 28,378 | 87.8 (87.5-88.2) | 0.2 | 10,796 | 86.8 (86.2-87.4) | 0.3 | 17,582 | 88.5 (88.0-88.9) | 0.2 |
|  | 6 | 27,610 | 86.8 (86.5-87.2) | 0.2 | 10,459 | 86.1 (85.4-86.7) | 0.3 | 17,151 | 87.3 (86.8-87.8) | 0.2 |
|  | 7 | 26,910 | 86.1 (85.7-86.5) | 0.2 | 10,220 | 85.5 (84.8-86.1) | 0.3 | 16,690 | 86.5 (85.9-86.9) | 0.3 |
|  | 8 | 26,185 | 85.4 (85.0-85.8) | 0.2 | 9,913 | 84.9 (84.3-85.6) | 0.3 | 16,272 | 85.7 (85.1-86.2) | 0.3 |
|  | 9 | 25,723 | 84.8 (84.4-85.2) | 0.2 | 9,771 | 84.4 (83.7-85.1) | 0.3 | 15,952 | 85.0 (84.5-85.5) | 0.3 |
|  | 10 | 25,061 | 84.1 (83.7-84.6) | 0.2 | 9,472 | 83.9 (83.2-84.6) | 0.4 | 15,589 | 84.3 (83.7-84.8) | 0.3 |
|  | 11 | 24,585 | 83.6 (83.1-84.0) | 0.2 | 9,196 | 83.4 (82.7-84.1) | 0.4 | 15,389 | 83.7 (83.1-84.2) | 0.3 |
|  | 12 | 24,088 | 83.0 (82.6-83.5) | 0.2 | 8,907 | 82.9 (82.2-83.6) | 0.4 | 15,181 | 83.1 (82.5-83.7) | 0.3 |
|  | 13 | 23,469 | 82.6 (82.2-83.1) | 0.2 | 8,634 | 82.7 (82.0-83.4) | 0.4 | 14,835 | 82.6 (82.0-83.2) | 0.3 |
|  | 14 | 23,001 | 82.2 (81.8-82.7) | 0.2 | 8,408 | 82.4 (81.7-83.1) | 0.4 | 14,593 | 82.1 (81.5-82.7) | 0.3 |
|  | 15 | 22,396 | 81.7 (81.3-82.2) | 0.2 | 8,138 | 82.1 (81.3-82.8) | 0.4 | 14,258 | 81.5 (80.9-82.1) | 0.3 |
| **Age at diagnosis (Years)** |  |  |  |  |  |  |  |  |  |  |
| 18-20 | 1 | 1,052 | 96.2 (94.9-97.2) | 0.6 | 590 | 96.6 (94.8-97.8) | 0.7 | 462 | 95.7 (93.4-97.2) | 0.9 |
|  | 2 | 1,100 | 93.4 (91.7-94.7) | 0.8 | 623 | 93.1 (90.7-94.9) | 1.0 | 477 | 93.7 (91.1-95.6) | 1.1 |
|  | 3 | 1,062 | 91.8 (90.0-93.4) | 0.9 | 603 | 91.3 (88.7-93.3) | 1.2 | 459 | 92.6 (89.8-94.7) | 1.2 |
|  | 4 | 1,042 | 90.6 (88.7-92.3) | 0.9 | 591 | 89.6 (86.8-91.8) | 1.3 | 451 | 92.0 (89.0-94.1) | 1.3 |
|  | 5 | 1,037 | 89.4 (87.4-91.1) | 1.0 | 574 | 89.0 (86.1-91.3) | 1.3 | 463 | 90.0 (86.8-92.4) | 1.4 |
|  | 6 | 1,018 | 88.9 (86.9-90.7) | 1.0 | 584 | 88.5 (85.6-90.8) | 1.3 | 434 | 89.5 (86.3-92.0) | 1.4 |
|  | 7 | 984 | 88.3 (86.2-90.1) | 1.0 | 566 | 87.8 (84.9-90.3) | 1.4 | 418 | 88.9 (85.6-91.5) | 1.5 |
|  | 8 | 958 | 88.0 (85.9-89.9) | 1.0 | 547 | 87.5 (84.5-90.0) | 1.4 | 411 | 88.7 (85.3-91.3) | 1.5 |
|  | 9 | 941 | 87.4 (85.2-89.3) | 1.0 | 555 | 86.7 (83.6-89.2) | 1.4 | 386 | 88.4 (85.1-91.1) | 1.5 |
|  | 10 | 909 | 87.0 (84.8-89.0) | 1.1 | 536 | 86.2 (83.1-88.8) | 1.4 | 373 | 88.2 (84.8-90.9) | 1.5 |
|  | 11 | 865 | 86.7 (84.5-88.7) | 1.1 | 499 | 86.0 (82.9-88.7) | 1.5 | 366 | 87.7 (84.2-90.4) | 1.6 |
|  | 12 | 836 | 86.7 (84.4-88.6) | 1.1 | 484 | 86.1 (83.0-88.7) | 1.5 | 352 | 87.4 (83.9-90.2) | 1.6 |
|  | 13 | 811 | 86.2 (83.9-88.2) | 1.1 | 473 | 85.9 (82.8-88.6) | 1.5 | 338 | 86.6 (83.0-89.5) | 1.7 |
|  | 14 | 798 | 86.1 (83.8-88.2) | 1.1 | 465 | 85.8 (82.6-88.4) | 1.5 | 333 | 86.6 (83.0-89.5) | 1.7 |
|  | 15 | 782 | 85.9 (83.6-88.0) | 1.1 | 459 | 85.4 (82.2-88.1) | 1.5 | 323 | 86.7 (83.1-89.6) | 1.7 |
| 21-24 | 1 | 2,334 | 96.9 (96.1-97.6) | 0.4 | 1,317 | 96.5 (95.3-97.3) | 0.5 | 1,017 | 97.6 (96.4-98.3) | 0.5 |
|  | 2 | 2,473 | 94.2 (93.2-95.1) | 0.5 | 1,380 | 93.8 (92.3-95.0) | 0.7 | 1,093 | 94.8 (93.3-96.0) | 0.7 |
|  | 3 | 2,354 | 92.7 (91.6-93.7) | 0.5 | 1,301 | 92.2 (90.6-93.5) | 0.8 | 1,053 | 93.4 (91.7-94.8) | 0.8 |
|  | 4 | 2,273 | 91.6 (90.4-92.7) | 0.6 | 1,247 | 91.0 (89.3-92.5) | 0.8 | 1,026 | 92.4 (90.6-93.9) | 0.8 |
|  | 5 | 2,203 | 90.8 (89.5-91.9) | 0.6 | 1,212 | 90.5 (88.8-92.0) | 0.8 | 991 | 91.1 (89.1-92.7) | 0.9 |
|  | 6 | 2,157 | 90.2 (88.9-91.4) | 0.6 | 1,178 | 90.0 (88.2-91.5) | 0.9 | 979 | 90.6 (88.6-92.2) | 0.9 |
|  | 7 | 2,076 | 89.7 (88.4-90.9) | 0.6 | 1,123 | 89.9 (88.0-91.4) | 0.9 | 953 | 89.7 (87.6-91.4) | 1.0 |
|  | 8 | 2,015 | 89.3 (88.0-90.6) | 0.7 | 1,097 | 89.4 (87.6-91.0) | 0.9 | 918 | 89.3 (87.2-91.0) | 1.0 |
|  | 9 | 1,972 | 89.1 (87.8-90.4) | 0.7 | 1,074 | 89.2 (87.3-90.8) | 0.9 | 898 | 89.1 (87.0-90.9) | 1.0 |
|  | 10 | 1,884 | 88.4 (87.0-89.7) | 0.7 | 1,013 | 88.7 (86.7-90.3) | 0.9 | 871 | 88.1 (85.9-90.0) | 1.0 |
|  | 11 | 1,784 | 87.8 (86.3-89.1) | 0.7 | 955 | 88.2 (86.2-89.9) | 0.9 | 829 | 87.3 (85.0-89.3) | 1.1 |
|  | 12 | 1,727 | 87.4 (85.9-88.8) | 0.7 | 932 | 87.7 (85.7-89.5) | 1.0 | 795 | 87.1 (84.8-89.1) | 1.1 |
|  | 13 | 1,637 | 87.3 (85.8-88.7) | 0.7 | 885 | 87.7 (85.7-89.5) | 1.0 | 752 | 87.0 (84.7-89.0) | 1.1 |
|  | 14 | 1,579 | 87.1 (85.6-88.5) | 0.7 | 859 | 87.2 (85.1-89.0) | 1.0 | 720 | 87.0 (84.7-89.0) | 1.1 |
|  | 15 | 1,546 | 87.0 (85.4-88.4) | 0.7 | 843 | 87.0 (84.9-88.9) | 1.0 | 703 | 86.9 (84.6-88.9) | 1.1 |
| 25-29 | 1 | 5,407 | 96.2 (95.7-96.7) | 0.3 | 2,542 | 96.1 (95.3-96.8) | 0.4 | 2,865 | 96.3 (95.5-96.9) | 0.4 |
|  | 2 | 5,605 | 93.5 (92.8-94.1) | 0.3 | 2,623 | 93.7 (92.7-94.6) | 0.5 | 2,982 | 93.3 (92.3-94.2) | 0.5 |
|  | 3 | 5,356 | 91.7 (91.0-92.4) | 0.4 | 2,539 | 92.1 (91.0-93.1) | 0.5 | 2,817 | 91.4 (90.3-92.3) | 0.5 |
|  | 4 | 5,185 | 90.5 (89.7-91.3) | 0.4 | 2,493 | 90.9 (89.7-92.0) | 0.6 | 2,692 | 90.2 (89.0-91.3) | 0.6 |
|  | 5 | 5,011 | 89.6 (88.7-90.4) | 0.4 | 2,384 | 90.2 (89.0-91.3) | 0.6 | 2,627 | 89.1 (87.8-90.2) | 0.6 |
|  | 6 | 4,787 | 88.7 (87.8-89.5) | 0.4 | 2,266 | 89.5 (88.2-90.6) | 0.6 | 2,521 | 88.0 (86.7-89.1) | 0.6 |
|  | 7 | 4,622 | 88.0 (87.1-88.9) | 0.5 | 2,197 | 88.9 (87.5-90.1) | 0.6 | 2,425 | 87.3 (86.0-88.5) | 0.6 |
|  | 8 | 4,380 | 87.6 (86.6-88.4) | 0.5 | 2,094 | 88.5 (87.1-89.7) | 0.7 | 2,286 | 86.7 (85.4-88.0) | 0.7 |
|  | 9 | 4,205 | 87.0 (86.0-87.9) | 0.5 | 2,021 | 88.0 (86.6-89.3) | 0.7 | 2,184 | 86.1 (84.7-87.4) | 0.7 |
|  | 10 | 3,983 | 86.6 (85.6-87.5) | 0.5 | 1,912 | 87.6 (86.2-88.9) | 0.7 | 2,071 | 85.6 (84.2-86.9) | 0.7 |
|  | 11 | 3,821 | 86.2 (85.2-87.1) | 0.5 | 1,822 | 87.2 (85.7-88.5) | 0.7 | 1,999 | 85.4 (83.9-86.7) | 0.7 |
|  | 12 | 3,695 | 85.8 (84.8-86.7) | 0.5 | 1,761 | 86.9 (85.4-88.2) | 0.7 | 1,934 | 84.8 (83.3-86.1) | 0.7 |
|  | 13 | 3,613 | 85.3 (84.3-86.3) | 0.5 | 1,735 | 86.6 (85.1-87.9) | 0.7 | 1,878 | 84.2 (82.7-85.6) | 0.7 |
|  | 14 | 3,536 | 85.1 (84.1-86.1) | 0.5 | 1,679 | 86.6 (85.1-87.9) | 0.7 | 1,857 | 83.9 (82.4-85.3) | 0.7 |
|  | 15 | 3,449 | 84.8 (83.7-85.8) | 0.5 | 1,602 | 86.2 (84.7-87.6) | 0.7 | 1,847 | 83.5 (82.0-84.9) | 0.8 |
| 30-34 | 1 | 9,101 | 96.1 (95.7-96.5) | 0.2 | 3,352 | 94.7 (93.8-95.4) | 0.4 | 5,749 | 97.0 (96.5-97.4) | 0.2 |
|  | 2 | 9,429 | 93.2 (92.7-93.7) | 0.3 | 3,453 | 91.7 (90.7-92.6) | 0.5 | 5,976 | 94.1 (93.4-94.7) | 0.3 |
|  | 3 | 8,806 | 91.4 (90.8-91.9) | 0.3 | 3,269 | 90.1 (89.1-91.1) | 0.5 | 5,537 | 92.1 (91.3-92.8) | 0.4 |
|  | 4 | 8,369 | 89.8 (89.1-90.4) | 0.3 | 3,112 | 88.6 (87.4-89.6) | 0.6 | 5,257 | 90.5 (89.7-91.2) | 0.4 |
|  | 5 | 7,986 | 88.7 (88.0-89.3) | 0.3 | 3,007 | 87.6 (86.4-88.7) | 0.6 | 4,979 | 89.3 (88.4-90.1) | 0.4 |
|  | 6 | 7,725 | 87.8 (87.1-88.5) | 0.4 | 2,900 | 87.3 (86.0-88.4) | 0.6 | 4,825 | 88.1 (87.2-88.9) | 0.4 |
|  | 7 | 7,491 | 87.1 (86.3-87.8) | 0.4 | 2,825 | 86.7 (85.4-87.8) | 0.6 | 4,666 | 87.3 (86.3-88.2) | 0.5 |
|  | 8 | 7,274 | 86.4 (85.7-87.1) | 0.4 | 2,739 | 86.1 (84.9-87.3) | 0.6 | 4,535 | 86.5 (85.6-87.4) | 0.5 |
|  | 9 | 7,194 | 85.8 (85.1-86.6) | 0.4 | 2,712 | 85.7 (84.4-86.8) | 0.6 | 4,482 | 85.9 (84.9-86.8) | 0.5 |
|  | 10 | 6,984 | 85.2 (84.4-86.0) | 0.4 | 2,612 | 85.2 (83.8-86.4) | 0.6 | 4,372 | 85.2 (84.2-86.2) | 0.5 |
|  | 11 | 6,894 | 84.6 (83.8-85.4) | 0.4 | 2,550 | 84.5 (83.1-85.7) | 0.7 | 4,344 | 84.6 (83.6-85.6) | 0.5 |
|  | 12 | 6,776 | 84.1 (83.2-84.9) | 0.4 | 2,484 | 83.8 (82.5-85.1) | 0.7 | 4,292 | 84.2 (83.1-85.1) | 0.5 |
|  | 13 | 6,662 | 83.8 (83.0-84.6) | 0.4 | 2,419 | 83.7 (82.3-85.0) | 0.7 | 4,243 | 83.8 (82.8-84.9) | 0.5 |
|  | 14 | 6,576 | 83.5 (82.7-84.3) | 0.4 | 2,351 | 83.5 (82.1-84.8) | 0.7 | 4,225 | 83.5 (82.4-84.5) | 0.5 |
|  | 15 | 6,523 | 83.0 (82.1-83.8) | 0.4 | 2,307 | 83.0 (81.5-84.3) | 0.7 | 4,216 | 83.0 (81.9-84.0) | 0.5 |
| 35-39 | 1 | 12,904 | 94.9 (94.5-95.2) | 0.2 | 4,047 | 92.0 (91.1-92.8) | 0.4 | 8,857 | 96.2 (95.7-96.6) | 0.2 |
|  | 2 | 13,418 | 91.3 (90.8-91.7) | 0.3 | 4,082 | 87.5 (86.4-88.5) | 0.5 | 9,336 | 93.0 (92.4-93.5) | 0.3 |
|  | 3 | 12,729 | 89.1 (88.6-89.7) | 0.3 | 3,802 | 85.4 (84.3-86.5) | 0.6 | 8,927 | 90.8 (90.2-91.4) | 0.3 |
|  | 4 | 12,436 | 87.4 (86.8-87.9) | 0.3 | 3,700 | 83.9 (82.7-85.0) | 0.6 | 8,736 | 89.0 (88.3-89.6) | 0.3 |
|  | 5 | 12,141 | 85.9 (85.3-86.5) | 0.3 | 3,619 | 82.5 (81.3-83.7) | 0.6 | 8,522 | 87.4 (86.7-88.1) | 0.4 |
|  | 6 | 11,923 | 84.7 (84.0-85.3) | 0.3 | 3,531 | 81.4 (80.1-82.6) | 0.6 | 8,392 | 86.2 (85.4-86.9) | 0.4 |
|  | 7 | 11,737 | 83.7 (83.1-84.4) | 0.3 | 3,509 | 80.6 (79.3-81.8) | 0.6 | 8,228 | 85.2 (84.4-85.9) | 0.4 |
|  | 8 | 11,558 | 82.9 (82.2-83.5) | 0.3 | 3,436 | 80.0 (78.6-81.2) | 0.6 | 8,122 | 84.2 (83.4-85.0) | 0.4 |
|  | 9 | 11,411 | 82.2 (81.5-82.9) | 0.3 | 3,409 | 79.4 (78.0-80.6) | 0.7 | 8,002 | 83.5 (82.7-84.3) | 0.4 |
|  | 10 | 11,301 | 81.5 (80.8-82.2) | 0.4 | 3,399 | 78.8 (77.4-80.1) | 0.7 | 7,902 | 82.7 (81.9-83.5) | 0.4 |
|  | 11 | 11,221 | 80.9 (80.2-81.6) | 0.4 | 3,370 | 78.4 (77.1-79.7) | 0.7 | 7,851 | 82.0 (81.2-82.8) | 0.4 |
|  | 12 | 11,054 | 80.2 (79.5-80.9) | 0.4 | 3,246 | 77.8 (76.4-79.1) | 0.7 | 7,808 | 81.3 (80.5-82.2) | 0.4 |
|  | 13 | 10,746 | 79.7 (79.0-80.5) | 0.4 | 3,122 | 77.5 (76.2-78.9) | 0.7 | 7,624 | 80.7 (79.9-81.6) | 0.4 |
|  | 14 | 10,512 | 79.1 (78.4-79.9) | 0.4 | 3,054 | 77.1 (75.7-78.4) | 0.7 | 7,458 | 80.1 (79.2-80.9) | 0.4 |
|  | 15 | 10,096 | 78.5 (77.8-79.3) | 0.4 | 2,927 | 76.8 (75.4-78.2) | 0.7 | 7,169 | 79.3 (78.4-80.2) | 0.5 |
| **Diagnostic group**** |  |  |  |  |  |  |  |  |  |  |
| **CNS and other intracranial and intraspinal neoplasms** |  |  |  |  |  |  |  |  |  |  |
|  | 1 | 1,342 | 91.1 (89.5-92.5) | 0.8 | 791 | 90.4 (88.1-92.2) | 1.1 | 551 | 92.2 (89.6-94.2) | 1.1 |
|  | 2 | 1,348 | 80.8 (78.6-82.8) | 1.1 | 798 | 79.0 (76.0-81.7) | 1.5 | 550 | 83.5 (80.1-86.4) | 1.6 |
|  | 3 | 1,190 | 75.0 (72.6-77.2) | 1.2 | 697 | 72.6 (69.4-75.6) | 1.6 | 493 | 78.5 (74.8-81.7) | 1.8 |
|  | 4 | 1,081 | 69.9 (67.4-72.3) | 1.3 | 631 | 67.7 (64.4-70.9) | 1.7 | 450 | 73.1 (69.1-76.7) | 1.9 |
|  | 5 | 998 | 65.7 (63.1-68.2) | 1.3 | 586 | 63.3 (59.9-66.6) | 1.7 | 412 | 69.2 (65.1-73.0) | 2.0 |
|  | 6 | 930 | 62.3 (59.6-64.9) | 1.3 | 534 | 60.4 (56.8-63.7) | 1.7 | 396 | 65.2 (60.9-69.1) | 2.1 |
|  | 7 | 859 | 58.9 (56.1-61.5) | 1.4 | 490 | 57.1 (53.5-60.5) | 1.8 | 369 | 61.5 (57.2-65.5) | 2.1 |
|  | 8 | 811 | 54.5 (51.8-57.2) | 1.4 | 468 | 53.0 (49.4-56.4) | 1.8 | 343 | 56.9 (52.5-61.0) | 2.2 |
|  | 9 | 735 | 51.9 (49.2-54.6) | 1.4 | 424 | 49.9 (46.3-53.4) | 1.8 | 311 | 54.9 (50.6-59.1) | 2.2 |
|  | 10 | 682 | 48.7 (46.0-51.5) | 1.4 | 381 | 46.7 (43.1-50.2) | 1.8 | 301 | 51.8 (47.4-56.0) | 2.2 |
|  | 11 | 637 | 45.8 (43.0-48.5) | 1.4 | 351 | 43.6 (40.0-47.1) | 1.8 | 286 | 49.0 (44.6-53.2) | 2.2 |
|  | 12 | 577 | 43.1 (40.4-45.9) | 1.4 | 314 | 40.6 (37.1-44.2) | 1.8 | 263 | 46.7 (42.3-51.0) | 2.2 |
|  | 13 | 522 | 41.5 (38.8-44.3) | 1.4 | 270 | 38.9 (35.3-42.4) | 1.8 | 252 | 45.3 (40.9-49.6) | 2.2 |
|  | 14 | 492 | 40.0 (37.2-42.8) | 1.4 | 260 | 37.6 (34.0-41.2) | 1.8 | 232 | 43.4 (39.1-47.7) | 2.2 |
|  | 15 | 470 | 38.7 (36.0-41.5) | 1.4 | 248 | 36.6 (33.1-40.2) | 1.8 | 222 | 41.7 (37.4-46.0) | 2.2 |
| **Sarcomas** |  |  |  |  |  |  |  |  |  |  |
| Soft tissue sarcomas | 1 | 601 | 91.9 (89.5-93.9) | 1.1 | 275 | 89.2 (85.0-92.4) | 1.9 | 326 | 94.2 (91.1-96.3) | 1.3 |
|  | 2 | 610 | 85.2 (82.1-87.8) | 1.4 | 272 | 81.7 (76.6-85.8) | 2.3 | 338 | 88.1 (84.1-91.2) | 1.8 |
|  | 3 | 580 | 82.3 (79.1-85.2) | 1.6 | 266 | 78.4 (73.1-82.8) | 2.5 | 314 | 85.7 (81.4-89.1) | 1.9 |
|  | 4 | 582 | 80.4 (77.0-83.3) | 1.6 | 264 | 75.5 (70.0-80.1) | 2.6 | 318 | 84.6 (80.2-88.1) | 2.0 |
|  | 5 | 598 | 78.9 (75.5-81.9) | 1.7 | 268 | 72.7 (67.1-77.5) | 2.6 | 330 | 84.3 (79.9-87.9) | 2.0 |
|  | 6 | 600 | 78.0 (74.5-81.0) | 1.7 | 257 | 72.1 (66.5-76.9) | 2.7 | 343 | 83.0 (78.5-86.7) | 2.1 |
|  | 7 | 616 | 77.6 (74.1-80.7) | 1.7 | 271 | 71.6 (66.0-76.4) | 2.7 | 345 | 82.8 (78.3-86.5) | 2.1 |
|  | 8 | 621 | 77.5 (74.0-80.6) | 1.7 | 265 | 71.3 (65.7-76.2) | 2.7 | 356 | 82.9 (78.3-86.5) | 2.1 |
|  | 9 | 629 | 76.2 (72.6-79.4) | 1.7 | 274 | 70.5 (64.9-75.5) | 2.7 | 355 | 81.1 (76.4-84.9) | 2.2 |
|  | 10 | 606 | 75.7 (72.1-78.9) | 1.7 | 274 | 70.0 (64.4-75.0) | 2.7 | 332 | 80.6 (75.9-84.5) | 2.2 |
|  | 11 | 624 | 75.2 (71.6-78.5) | 1.7 | 283 | 69.3 (63.7-74.3) | 2.7 | 341 | 80.4 (75.7-84.3) | 2.2 |
|  | 12 | 637 | 75.1 (71.5-78.3) | 1.7 | 290 | 69.4 (63.7-74.4) | 2.7 | 347 | 80.0 (75.2-83.9) | 2.2 |
|  | 13 | 661 | 74.9 (71.3-78.2) | 1.7 | 310 | 69.3 (63.6-74.3) | 2.7 | 351 | 79.8 (75.1-83.8) | 2.2 |
|  | 14 | 668 | 74.8 (71.2-78.0) | 1.8 | 314 | 69.4 (63.7-74.4) | 2.7 | 354 | 79.4 (74.6-83.4) | 2.2 |
|  | 15 | 672 | 74.3 (70.6-77.6) | 1.8 | 315 | 68.8 (63.1-73.8) | 2.7 | 357 | 79.0 (74.2-83.1) | 2.2 |
| Bone sarcomas | 1 | 425 | 95.6 (93.1-97.2) | 1.0 | 250 | 96.0 (92.7-97.9) | 1.2 | 175 | 94.9 (90.4-97.3) | 1.7 |
|  | 2 | 461 | 90.8 (87.6-93.2) | 1.4 | 265 | 89.7 (85.2-92.9) | 1.9 | 196 | 92.2 (87.2-95.3) | 2.0 |
|  | 3 | 464 | 86.3 (82.7-89.2) | 1.6 | 253 | 84.0 (78.9-88.0) | 2.3 | 211 | 89.3 (83.8-93.0) | 2.3 |
|  | 4 | 455 | 84.0 (80.3-87.1) | 1.7 | 250 | 80.7 (75.4-85.1) | 2.5 | 205 | 88.4 (82.8-92.2) | 2.4 |
|  | 5 | 454 | 83.1 (79.3-86.3) | 1.8 | 243 | 80.1 (74.6-84.5) | 2.5 | 211 | 87.0 (81.4-91.1) | 2.4 |
|  | 6 | 472 | 82.0 (78.1-85.3) | 1.8 | 255 | 78.8 (73.3-83.3) | 2.5 | 217 | 86.2 (80.5-90.4) | 2.5 |
|  | 7 | 476 | 81.7 (77.8-85.0) | 1.8 | 252 | 78.5 (73.0-83.0) | 2.6 | 224 | 85.9 (80.1-90.1) | 2.5 |
|  | 8 | 470 | 81.0 (77.0-84.3) | 1.8 | 249 | 77.2 (71.6-81.8) | 2.6 | 221 | 85.9 (80.1-90.2) | 2.5 |
|  | 9 | 455 | 80.5 (76.5-83.8) | 1.9 | 238 | 76.9 (71.3-81.6) | 2.6 | 217 | 85.1 (79.3-89.5) | 2.6 |
|  | 10 | 437 | 80.3 (76.4-83.7) | 1.9 | 225 | 76.6 (70.9-81.3) | 2.6 | 212 | 85.2 (79.3-89.5) | 2.6 |
|  | 11 | 419 | 80.0 (76.0-83.4) | 1.9 | 207 | 76.2 (70.6-81.0) | 2.7 | 212 | 84.8 (78.9-89.2) | 2.6 |
|  | 12 | 397 | 79.2 (75.1-82.7) | 1.9 | 191 | 75.0 (69.2-79.9) | 2.7 | 206 | 84.4 (78.5-88.9) | 2.6 |
|  | 13 | 370 | 78.8 (74.7-82.3) | 1.9 | 179 | 74.2 (68.3-79.2) | 2.8 | 191 | 84.5 (78.6-89.0) | 2.6 |
|  | 14 | 338 | 78.4 (74.2-82.0) | 2.0 | 170 | 73.3 (67.3-78.4) | 2.8 | 168 | 84.6 (78.7-89.1) | 2.6 |
|  | 15 | 318 | 78.5 (74.3-82.1) | 2.0 | 153 | 73.4 (67.4-78.5) | 2.8 | 165 | 84.7 (78.8-89.2) | 2.6 |
| Gastrointestinal stromal tumors (GIST) | 1 | 75 | 100.1 (100.1-100.1) | 0.0 | 43 | 100.1 (100.1-100.1) | 0.0 | 32 | 100.0 (100.0-100.0) | 0.0 |
|  | 2 | 76 | 97.2 (89.0-99.4) | 2.0 | 44 | 100.1 (100.1-100.1) | 0.0 | 32 | 93.2 (75.4-98.3) | 4.7 |
|  | 3 | 67 | 97.3 (89.1-99.4) | 2.0 | 39 | 100.2 (100.2-100.2) | 0.0 | 28 | 93.3 (75.4-98.4) | 4.7 |
|  | 4 | 61 | 93.8 (83.8-97.8) | 3.1 | 32 | 93.7 (76.6-98.6) | 4.5 | 29 | 93.3 (75.5-98.4) | 4.7 |
|  | 5 | 57 | 93.8 (83.9-97.8) | 3.1 | 31 | 93.8 (76.6-98.7) | 4.5 | 26 | 93.4 (75.5-98.5) | 4.7 |
|  | 6 | 58 | 93.9 (84.0-97.9) | 3.1 | 31 | 93.9 (76.7-98.8) | 4.5 | 27 | 93.5 (75.6-98.6) | 4.7 |
|  | 7 | 57 | 94.0 (84.0-98.0) | 3.2 | 30 | 94.0 (76.8-98.9) | 4.5 | 27 | 93.5 (75.6-98.6) | 4.7 |
|  | 8 | 58 | 90.4 (79.3-95.9) | 4.0 | 29 | 86.9 (68.0-95.3) | 6.4 | 29 | 93.6 (75.7-98.7) | 4.7 |
|  | 9 | 56 | 85.3 (73.0-92.4) | 4.8 | 29 | 80.5 (61.0-91.1) | 7.4 | 27 | 89.9 (71.0-97.0) | 5.8 |
|  | 10 | 53 | 85.4 (73.1-92.5) | 4.8 | 27 | 80.6 (61.1-91.2) | 7.4 | 26 | 90.0 (71.1-97.1) | 5.8 |
|  | 11 | 50 | 83.6 (71.1-91.3) | 5.0 | 25 | 77.0 (57.0-88.8) | 7.9 | 25 | 90.1 (71.2-97.2) | 5.8 |
|  | 12 | 48 | 78.3 (65.0-87.2) | 5.6 | 23 | 70.2 (50.0-83.7) | 8.6 | 25 | 86.4 (66.7-95.2) | 6.7 |
|  | 13 | 49 | 76.6 (63.2-85.9) | 5.7 | 23 | 70.3 (50.1-83.9) | 8.6 | 26 | 82.8 (62.4-93.0) | 7.4 |
|  | 14 | 47 | 75.0 (61.4-84.6) | 5.9 | 22 | 70.5 (50.2-84.1) | 8.6 | 25 | 79.0 (58.2-90.6) | 8.0 |
|  | 15 | 42 | 75.1 (61.5-84.8) | 5.9 | 22 | 70.6 (50.3-84.2) | 8.7 | 20 | 79.2 (58.3-90.8) | 8.0 |
| Other sarcomas | 1 | 119 | 77.7 (69.2-84.1) | 3.8 | 55 | 77.2 (64.0-86.1) | 5.6 | 64 | 78.2 (66.0-86.4) | 5.1 |
|  | 2 | 103 | 72.6 (63.6-79.6) | 4.1 | 46 | 73.3 (59.7-83.0) | 5.9 | 57 | 72.0 (59.3-81.3) | 5.6 |
|  | 3 | 91 | 69.1 (59.9-76.6) | 4.2 | 41 | 71.4 (57.6-81.5) | 6.1 | 50 | 67.2 (54.3-77.3) | 5.9 |
|  | 4 | 89 | 67.5 (58.2-75.1) | 4.3 | 42 | 67.7 (53.7-78.4) | 6.3 | 47 | 67.3 (54.3-77.3) | 5.9 |
|  | 5 | 89 | 65.1 (55.8-72.9) | 4.4 | 41 | 66.0 (51.9-76.9) | 6.4 | 48 | 64.4 (51.4-74.7) | 6.0 |
|  | 6 | 88 | 65.1 (55.8-72.9) | 4.4 | 42 | 66.0 (51.9-76.9) | 6.4 | 46 | 64.4 (51.4-74.8) | 6.0 |
|  | 7 | 94 | 65.2 (55.9-73.0) | 4.4 | 45 | 66.1 (52.0-77.0) | 6.4 | 49 | 64.5 (51.5-74.8) | 6.0 |
|  | 8 | 97 | 64.4 (55.1-72.3) | 4.4 | 47 | 66.1 (52.0-77.0) | 6.4 | 50 | 63.0 (50.0-73.5) | 6.0 |
|  | 9 | 97 | 63.8 (54.4-71.7) | 4.4 | 45 | 66.2 (52.0-77.1) | 6.4 | 52 | 61.7 (48.7-72.3) | 6.1 |
|  | 10 | 102 | 61.7 (52.4-69.7) | 4.4 | 45 | 63.1 (49.0-74.3) | 6.5 | 57 | 60.5 (47.6-71.2) | 6.1 |
|  | 11 | 98 | 61.8 (52.5-69.8) | 4.4 | 43 | 63.2 (49.1-74.4) | 6.5 | 55 | 60.5 (47.6-71.3) | 6.1 |
|  | 12 | 93 | 61.8 (52.5-69.9) | 4.4 | 40 | 63.2 (49.1-74.5) | 6.5 | 53 | 60.6 (47.7-71.3) | 6.1 |
|  | 13 | 94 | 61.9 (52.6-70.0) | 4.4 | 40 | 63.3 (49.2-74.6) | 6.5 | 54 | 60.7 (47.7-71.4) | 6.1 |
|  | 14 | 87 | 61.2 (51.9-69.3) | 4.5 | 37 | 63.4 (49.3-74.7) | 6.5 | 50 | 59.5 (46.6-70.3) | 6.1 |
|  | 15 | 85 | 60.5 (51.2-68.7) | 4.5 | 33 | 61.3 (47.1-73.0) | 6.7 | 52 | 59.6 (46.7-70.4) | 6.1 |
| **Blood and lymphatic vessel tumors** |  |  |  |  |  |  |  |  |  |  |
|  | 1 | 147 | 84.7 (77.7-89.7) | 3.0 | 102 | 85.9 (77.4-91.4) | 3.5 | 45 | 82.1 (67.3-90.6) | 5.7 |
|  | 2 | 138 | 80.0 (72.5-85.7) | 3.3 | 102 | 83.2 (74.3-89.2) | 3.7 | 36 | 71.9 (55.7-83.0) | 6.9 |
|  | 3 | 133 | 78.7 (71.1-84.5) | 3.4 | 104 | 82.3 (73.4-88.5) | 3.8 | 29 | 68.9 (52.2-80.7) | 7.3 |
|  | 4 | 132 | 78.7 (71.1-84.6) | 3.4 | 106 | 82.4 (73.4-88.6) | 3.8 | 26 | 68.9 (52.2-80.8) | 7.3 |
|  | 5 | 134 | 78.1 (70.4-84.1) | 3.5 | 107 | 81.6 (72.6-88.0) | 3.9 | 27 | 68.9 (52.2-80.8) | 7.3 |
|  | 6 | 133 | 77.5 (69.8-83.5) | 3.5 | 108 | 80.8 (71.7-87.3) | 3.9 | 25 | 69.0 (52.3-80.9) | 7.3 |
|  | 7 | 138 | 77.6 (69.8-83.6) | 3.5 | 112 | 80.9 (71.8-87.4) | 3.9 | 26 | 69.0 (52.3-80.9) | 7.3 |
|  | 8 | 143 | 77.7 (69.9-83.7) | 3.5 | 116 | 81.0 (71.8-87.5) | 3.9 | 27 | 69.0 (52.3-80.9) | 7.3 |
|  | 9 | 146 | 77.2 (69.4-83.3) | 3.5 | 117 | 81.1 (71.9-87.6) | 3.9 | 29 | 66.4 (49.6-78.8) | 7.5 |
|  | 10 | 151 | 76.7 (68.9-82.9) | 3.5 | 121 | 81.2 (72.0-87.7) | 3.9 | 30 | 64.0 (47.2-76.8) | 7.6 |
|  | 11 | 155 | 76.3 (68.4-82.5) | 3.6 | 124 | 80.5 (71.4-87.1) | 4.0 | 31 | 64.1 (47.2-76.9) | 7.6 |
|  | 12 | 148 | 76.4 (68.5-82.6) | 3.6 | 120 | 80.7 (71.5-87.3) | 4.0 | 28 | 64.1 (47.2-76.9) | 7.6 |
|  | 13 | 151 | 75.4 (67.5-81.7) | 3.6 | 122 | 80.1 (70.9-86.8) | 4.0 | 29 | 61.8 (45.0-75.0) | 7.7 |
|  | 14 | 148 | 74.4 (66.5-80.8) | 3.6 | 122 | 78.8 (69.5-85.7) | 4.1 | 26 | 61.9 (45.1-75.1) | 7.7 |
|  | 15 | 143 | 74.5 (66.6-81.0) | 3.7 | 118 | 78.9 (69.6-85.8) | 4.1 | 25 | 62.0 (45.1-75.2) | 7.7 |
| **Nerve sheath tumors** |  |  |  |  |  |  |  |  |  |  |
|  | 1 | 76 | 78.8 (67.8-86.4) | 4.7 | 40 | 77.2 (60.7-87.4) | 6.7 | 36 | 80.6 (63.6-90.2) | 6.6 |
|  | 2 | 63 | 64.9 (52.8-74.6) | 5.6 | 34 | 62.1 (45.2-75.1) | 7.7 | 29 | 68.1 (49.7-81.0) | 8.0 |
|  | 3 | 52 | 58.2 (46.2-68.5) | 5.7 | 29 | 55.4 (39.0-69.1) | 7.8 | 23 | 61.7 (43.1-75.8) | 8.4 |
|  | 4 | 49 | 58.3 (46.2-68.6) | 5.7 | 27 | 55.4 (39.0-69.1) | 7.8 | 22 | 61.7 (43.1-75.8) | 8.4 |
|  | 5 | 48 | 58.3 (46.2-68.6) | 5.7 | 28 | 55.5 (39.0-69.1) | 7.8 | 20 | 61.7 (43.1-75.8) | 8.4 |
|  | 6 | 43 | 56.8 (44.7-67.2) | 5.8 | 25 | 53.1 (36.8-67.0) | 7.8 | 18 | 61.8 (43.2-75.9) | 8.5 |
|  | 7 | 44 | 55.3 (43.1-65.9) | 5.9 | 25 | 50.6 (34.5-64.7) | 7.9 | 19 | 61.8 (43.2-75.9) | 8.5 |
|  | 8 | 44 | 55.3 (43.2-65.9) | 5.9 | 26 | 50.7 (34.5-64.8) | 7.9 | 18 | 61.8 (43.2-76.0) | 8.5 |
|  | 9 | 39 | 55.4 (43.2-66.0) | 5.9 | 21 | 50.7 (34.6-64.8) | 7.9 | 18 | 61.9 (43.2-76.0) | 8.5 |
|  | 10 | 39 | 55.4 (43.2-66.0) | 5.9 | 20 | 50.8 (34.6-64.9) | 7.9 | 19 | 61.9 (43.3-76.0) | 8.5 |
|  | 11 | 42 | 55.5 (43.3-66.1) | 5.9 | 21 | 50.8 (34.6-65.0) | 7.9 | 21 | 61.9 (43.3-76.1) | 8.5 |
|  | 12 | 46 | 55.5 (43.3-66.1) | 5.9 | 22 | 50.9 (34.7-65.1) | 7.9 | 24 | 62.0 (43.3-76.2) | 8.5 |
|  | 13 | 47 | 54.3 (42.1-65.0) | 5.9 | 24 | 51.0 (34.7-65.2) | 7.9 | 23 | 59.2 (40.7-73.7) | 8.6 |
|  | 14 | 45 | 54.4 (42.2-65.1) | 5.9 | 23 | 51.0 (34.8-65.3) | 7.9 | 22 | 59.3 (40.7-73.8) | 8.6 |
|  | 15 | 44 | 54.4 (42.3-65.2) | 5.9 | 21 | 51.1 (34.8-65.4) | 7.9 | 23 | 59.3 (40.8-73.9) | 8.6 |
| **Gonadal and related tumors** |  |  |  |  |  |  |  |  |  |  |
| Testis, Germ cell and trophoblastic | 1 | 5,217 | 99.6 (99.3-99.7) | 0.1 | 5,217 | 99.6 (99.3-99.7) | 0.1 | NA | NA | NA |
|  | 2 | 5,607 | 99.1 (98.8-99.4) | 0.1 | 5,607 | 99.1 (98.8-99.4) | 0.1 | NA | NA | NA |
|  | 3 | 5,412 | 98.8 (98.5-99.1) | 0.2 | 5,412 | 98.8 (98.5-99.1) | 0.2 | NA | NA | NA |
|  | 4 | 5,285 | 98.8 (98.4-99.1) | 0.2 | 5,285 | 98.8 (98.4-99.1) | 0.2 | NA | NA | NA |
|  | 5 | 5,153 | 98.8 (98.4-99.1) | 0.2 | 5,153 | 98.8 (98.4-99.1) | 0.2 | NA | NA | NA |
|  | 6 | 4,983 | 98.8 (98.4-99.1) | 0.2 | 4,983 | 98.8 (98.4-99.1) | 0.2 | NA | NA | NA |
|  | 7 | 4,822 | 98.7 (98.3-99.1) | 0.2 | 4,822 | 98.7 (98.3-99.1) | 0.2 | NA | NA | NA |
|  | 8 | 4,655 | 98.8 (98.4-99.1) | 0.2 | 4,655 | 98.8 (98.4-99.1) | 0.2 | NA | NA | NA |
|  | 9 | 4,578 | 98.8 (98.3-99.1) | 0.2 | 4,578 | 98.8 (98.3-99.1) | 0.2 | NA | NA | NA |
|  | 10 | 4,391 | 98.7 (98.2-99.0) | 0.2 | 4,391 | 98.7 (98.2-99.0) | 0.2 | NA | NA | NA |
|  | 11 | 4,246 | 98.6 (98.1-99.0) | 0.2 | 4,246 | 98.6 (98.1-99.0) | 0.2 | NA | NA | NA |
|  | 12 | 4,159 | 98.6 (98.1-99.0) | 0.2 | 4,159 | 98.6 (98.1-99.0) | 0.2 | NA | NA | NA |
|  | 13 | 4,037 | 98.7 (98.2-99.1) | 0.2 | 4,037 | 98.7 (98.2-99.1) | 0.2 | NA | NA | NA |
|  | 14 | 3,925 | 98.6 (98.1-99.0) | 0.2 | 3,925 | 98.6 (98.1-99.0) | 0.2 | NA | NA | NA |
|  | 15 | 3,783 | 98.5 (97.9-98.9) | 0.3 | 3,783 | 98.5 (97.9-98.9) | 0.3 | NA | NA | NA |
| Testis, Non-germ cell | 1 | 25 | 100.1 (100.1-100.1) | 0.0 | 25 | 100.1 (100.1-100.1) | 0.0 | NA | NA | NA |
|  | 2 | 25 | 100.1 (100.1-100.1) | 0.0 | 25 | 100.1 (100.1-100.1) | 0.0 | NA | NA | NA |
|  | 3 | 23 | 100.2 (100.2-100.2) | 0.0 | 23 | 100.2 (100.2-100.2) | 0.0 | NA | NA | NA |
|  | 4 | 19 | 94.5 (65.7-99.4) | 5.6 | 19 | 94.5 (65.7-99.4) | 5.6 | NA | NA | NA |
|  | 5 | 17 | 94.5 (65.8-99.5) | 5.6 | 17 | 94.5 (65.8-99.5) | 5.6 | NA | NA | NA |
|  | 6 | 18 | 94.6 (65.8-99.6) | 5.6 | 18 | 94.6 (65.8-99.6) | 5.6 | NA | NA | NA |
|  | 7 | 17 | 94.7 (65.9-99.6) | 5.6 | 17 | 94.7 (65.9-99.6) | 5.6 | NA | NA | NA |
|  | 8 | 15 | 94.8 (65.9-99.7) | 5.6 | 15 | 94.8 (65.9-99.7) | 5.6 | NA | NA | NA |
|  | 9 | 14 | 94.9 (66.0-99.8) | 5.6 | 14 | 94.9 (66.0-99.8) | 5.6 | NA | NA | NA |
|  | 10 | 14 | 95.0 (66.1-99.9) | 5.6 | 14 | 95.0 (66.1-99.9) | 5.6 | NA | NA | NA |
|  | 11 | 12 | 95.1 (66.1-100.0) | 5.7 | 12 | 95.1 (66.1-100) | 5.7 | NA | NA | NA |
|  | 12 | 9 | 95.2 (66.2-100.2) | 5.7 | 9 | 95.2 (66.2-100.2) | 5.7 | NA | NA | NA |
|  | 13 | 10 | 95.3 (66.3-100.3) | 5.7 | 10 | 95.3 (66.3-100.3) | 5.7 | NA | NA | NA |
|  | 14 | 11 | 95.4 (66.4-100.4) | 5.7 | 11 | 95.4 (66.4-100.4) | 5.7 | NA | NA | NA |
|  | 15 | 13 | 95.6 (66.5-100.6) | 5.7 | 13 | 95.6 (66.5-100.6) | 5.7 | NA | NA | NA |
| Ovary, Germ cell and trophoblastic | 1 | 126 | 98.4 (93.8-99.6) | 1.1 | NA | NA | NA | 126 | 98.4 (93.8-99.6) | 1.1 |
|  | 2 | 134 | 97.7 (92.9-99.3) | 1.3 | NA | NA | NA | 134 | 97.7 (92.9-99.3) | 1.3 |
|  | 3 | 139 | 96.9 (91.9-98.9) | 1.6 | NA | NA | NA | 139 | 96.9 (91.9-98.9) | 1.6 |
|  | 4 | 129 | 97.0 (91.9-98.9) | 1.6 | NA | NA | NA | 129 | 97.0 (91.9-98.9) | 1.6 |
|  | 5 | 120 | 97.0 (91.9-99.0) | 1.6 | NA | NA | NA | 120 | 97.0 (91.9-99.0) | 1.6 |
|  | 6 | 111 | 97.0 (92.0-99.0) | 1.6 | NA | NA | NA | 111 | 97.0 (92.0-99.0) | 1.6 |
|  | 7 | 104 | 97.1 (92.0-99.0) | 1.6 | NA | NA | NA | 104 | 97.1 (92.0-99.0) | 1.6 |
|  | 8 | 104 | 97.1 (92.0-99.1) | 1.6 | NA | NA | NA | 104 | 97.1 (92.0-99.1) | 1.6 |
|  | 9 | 103 | 97.1 (92.1-99.1) | 1.6 | NA | NA | NA | 103 | 97.1 (92.1-99.1) | 1.6 |
|  | 10 | 106 | 97.2 (92.1-99.2) | 1.6 | NA | NA | NA | 106 | 97.2 (92.1-99.2) | 1.6 |
|  | 11 | 110 | 97.3 (92.2-99.2) | 1.6 | NA | NA | NA | 110 | 97.3 (92.2-99.2) | 1.6 |
|  | 12 | 106 | 97.3 (92.3-99.3) | 1.6 | NA | NA | NA | 106 | 97.3 (92.3-99.3) | 1.6 |
|  | 13 | 103 | 96.3 (90.6-98.8) | 1.9 | NA | NA | NA | 103 | 96.3 (90.6-98.8) | 1.9 |
|  | 14 | 95 | 96.4 (90.7-98.9) | 1.9 | NA | NA | NA | 95 | 96.4 (90.7-98.9) | 1.9 |
|  | 15 | 90 | 95.4 (89.1-98.3) | 2.2 | NA | NA | NA | 90 | 95.4 (89.1-98.3) | 2.2 |
| Ovary, Non-germ cell | 1 | 369 | 90.5 (87.0-93.1) | 1.5 | NA | NA | NA | 369 | 90.5 (87.0-93.1) | 1.5 |
|  | 2 | 360 | 82.8 (78.5-86.3) | 2.0 | NA | NA | NA | 360 | 82.8 (78.5-86.3) | 2.0 |
|  | 3 | 323 | 76.3 (71.6-80.4) | 2.2 | NA | NA | NA | 323 | 76.3 (71.6-80.4) | 2.2 |
|  | 4 | 278 | 71.5 (66.5-75.9) | 2.4 | NA | NA | NA | 278 | 71.5 (66.5-75.9) | 2.4 |
|  | 5 | 250 | 66.1 (60.8-70.8) | 2.6 | NA | NA | NA | 250 | 66.1 (60.8-70.8) | 2.6 |
|  | 6 | 225 | 64.2 (58.8-69.0) | 2.6 | NA | NA | NA | 225 | 64.2 (58.8-69.0) | 2.6 |
|  | 7 | 225 | 62.9 (57.5-67.8) | 2.6 | NA | NA | NA | 225 | 62.9 (57.5-67.8) | 2.6 |
|  | 8 | 206 | 62.6 (57.2-67.6) | 2.6 | NA | NA | NA | 206 | 62.6 (57.2-67.6) | 2.6 |
|  | 9 | 208 | 61.3 (55.9-66.3) | 2.7 | NA | NA | NA | 208 | 61.3 (55.9-66.3) | 2.7 |
|  | 10 | 203 | 60.0 (54.5-65.1) | 2.7 | NA | NA | NA | 203 | 60.0 (54.5-65.1) | 2.7 |
|  | 11 | 205 | 60.1 (54.6-65.2) | 2.7 | NA | NA | NA | 205 | 60.1 (54.6-65.2) | 2.7 |
|  | 12 | 209 | 59.5 (54.0-64.6) | 2.7 | NA | NA | NA | 209 | 59.5 (54.0-64.6) | 2.7 |
|  | 13 | 259 | 58.8 (53.2-63.9) | 2.7 | NA | NA | NA | 259 | 58.8 (53.2-63.9) | 2.7 |
|  | 14 | 322 | 58.9 (53.3-64.0) | 2.7 | NA | NA | NA | 322 | 58.9 (53.3-64.0) | 2.7 |
|  | 15 | 403 | 58.6 (53.1-63.7) | 2.7 | NA | NA | NA | 403 | 58.6 (53.1-63.7) | 2.7 |
| (Other) Germ cell, non-germ cell and trophoblastic tumors | 1 | 187 | 93.6 (89.0-96.3) | 1.8 | 122 | 92.7 (86.4-96.2) | 2.4 | 65 | 95.3 (86.1-98.5) | 2.7 |
|  | 2 | 193 | 91.4 (86.4-94.7) | 2.1 | 119 | 89.3 (82.2-93.7) | 2.8 | 74 | 95.3 (86.2-98.5) | 2.7 |
|  | 3 | 174 | 91.5 (86.4-94.8) | 2.1 | 104 | 89.4 (82.3-93.7) | 2.8 | 70 | 95.4 (86.2-98.6) | 2.7 |
|  | 4 | 166 | 90.3 (85.0-93.9) | 2.2 | 95 | 88.4 (81.1-93.1) | 3.0 | 71 | 93.9 (84.4-97.8) | 3.0 |
|  | 5 | 161 | 89.8 (84.3-93.4) | 2.3 | 90 | 87.3 (79.7-92.3) | 3.1 | 71 | 94.0 (84.5-97.8) | 3.0 |
|  | 6 | 161 | 89.8 (84.3-93.5) | 2.3 | 86 | 87.4 (79.7-92.4) | 3.1 | 75 | 94.0 (84.5-97.9) | 3.0 |
|  | 7 | 164 | 89.3 (83.7-93.1) | 2.3 | 85 | 87.5 (79.8-92.4) | 3.1 | 79 | 92.8 (83.1-97.1) | 3.2 |
|  | 8 | 166 | 89.3 (83.7-93.1) | 2.3 | 86 | 87.5 (79.8-92.5) | 3.1 | 80 | 92.8 (83.2-97.2) | 3.2 |
|  | 9 | 169 | 88.8 (83.1-92.7) | 2.4 | 91 | 86.5 (78.6-91.8) | 3.3 | 78 | 92.9 (83.3-97.2) | 3.2 |
|  | 10 | 163 | 88.9 (83.2-92.8) | 2.4 | 90 | 86.6 (78.7-91.9) | 3.3 | 73 | 93.0 (83.3-97.3) | 3.2 |
|  | 11 | 161 | 89.0 (83.3-92.9) | 2.4 | 88 | 86.7 (78.7-91.9) | 3.3 | 73 | 93.0 (83.4-97.4) | 3.2 |
|  | 12 | 156 | 87.8 (81.9-92.0) | 2.5 | 85 | 85.7 (77.5-91.2) | 3.4 | 71 | 91.7 (81.7-96.5) | 3.5 |
|  | 13 | 148 | 87.9 (82.0-92.1) | 2.5 | 86 | 85.8 (77.6-91.3) | 3.4 | 62 | 91.8 (81.8-96.6) | 3.5 |
|  | 14 | 150 | 88.0 (82.1-92.2) | 2.5 | 85 | 85.9 (77.7-91.4) | 3.4 | 65 | 91.9 (81.9-96.7) | 3.5 |
|  | 15 | 159 | 87.5 (81.4-91.8) | 2.6 | 93 | 85.0 (76.6-90.7) | 3.5 | 66 | 92.0 (82.0-96.9) | 3.5 |
| **Melanoma, malignant** |  |  |  |  |  |  |  |  |  |  |
|  | 1 | 5,570 | 99.3 (99.0-99.5) | 0.1 | 1,945 | 98.7 (98.1-99.1) | 0.3 | 3,625 | 99.6 (99.3-99.7) | 0.1 |
|  | 2 | 6,111 | 98.2 (97.8-98.6) | 0.2 | 2,124 | 96.9 (96.0-97.6) | 0.4 | 3,987 | 99.0 (98.6-99.3) | 0.2 |
|  | 3 | 6,085 | 97.7 (97.2-98.1) | 0.2 | 2,093 | 95.9 (94.9-96.7) | 0.5 | 3,992 | 98.7 (98.2-99.0) | 0.2 |
|  | 4 | 6,198 | 97.0 (96.5-97.4) | 0.2 | 2,101 | 94.5 (93.4-95.5) | 0.5 | 4,097 | 98.3 (97.8-98.7) | 0.2 |
|  | 5 | 6,201 | 96.3 (95.7-96.8) | 0.3 | 2,068 | 93.4 (92.2-94.4) | 0.6 | 4,133 | 97.8 (97.3-98.3) | 0.3 |
|  | 6 | 6,168 | 95.8 (95.2-96.3) | 0.3 | 2,046 | 92.8 (91.5-93.9) | 0.6 | 4,122 | 97.4 (96.8-97.9) | 0.3 |
|  | 7 | 6,157 | 95.2 (94.6-95.8) | 0.3 | 2,071 | 92.0 (90.6-93.1) | 0.6 | 4,086 | 97.0 (96.4-97.5) | 0.3 |
|  | 8 | 6,113 | 94.8 (94.2-95.4) | 0.3 | 2,016 | 91.6 (90.3-92.8) | 0.6 | 4,097 | 96.5 (95.9-97.1) | 0.3 |
|  | 9 | 6,061 | 94.5 (93.8-95.1) | 0.3 | 1,995 | 91.2 (89.8-92.4) | 0.7 | 4,066 | 96.2 (95.5-96.8) | 0.3 |
|  | 10 | 6,051 | 94.2 (93.5-94.8) | 0.3 | 1,993 | 90.8 (89.3-92.0) | 0.7 | 4,058 | 96.0 (95.3-96.6) | 0.3 |
|  | 11 | 6,040 | 93.8 (93.2-94.5) | 0.3 | 1,977 | 90.4 (89.0-91.7) | 0.7 | 4,063 | 95.6 (94.9-96.3) | 0.4 |
|  | 12 | 5,989 | 93.6 (92.9-94.3) | 0.3 | 1,921 | 90.1 (88.6-91.4) | 0.7 | 4,068 | 95.5 (94.8-96.2) | 0.4 |
|  | 13 | 5,829 | 93.4 (92.7-94.1) | 0.4 | 1,862 | 89.9 (88.5-91.3) | 0.7 | 3,967 | 95.2 (94.4-95.9) | 0.4 |
|  | 14 | 5,726 | 93.3 (92.6-94.0) | 0.4 | 1,823 | 89.8 (88.3-91.1) | 0.7 | 3,903 | 95.2 (94.4-95.9) | 0.4 |
|  | 15 | 5,518 | 93.2 (92.4-93.9) | 0.4 | 1,776 | 89.6 (88.1-91.0) | 0.7 | 3,742 | 95.1 (94.2-95.8) | 0.4 |
| **Carcinomas** |  |  |  |  |  |  |  |  |  |  |
| Thyroid carcinoma |  |  |  |  |  |  |  |  |  |  |
|  | 1 | 1,560 | 99.8 (99.4-99.9) | 0.1 | 361 | 99.5 (97.9-99.9) | 0.4 | 1,199 | 99.9 (99.4-100.0) | 0.1 |
|  | 2 | 1,668 | 99.7 (99.2-99.9) | 0.2 | 386 | 99.0 (97.1-99.7) | 0.6 | 1,282 | 99.9 (99.4-100.0) | 0.1 |
|  | 3 | 1,604 | 99.6 (99.1-99.9) | 0.2 | 365 | 99.0 (97.2-99.8) | 0.6 | 1,239 | 99.8 (99.2-100.0) | 0.2 |
|  | 4 | 1,552 | 99.5 (98.9-99.8) | 0.2 | 342 | 98.5 (96.4-99.5) | 0.7 | 1,210 | 99.8 (99.2-100.0) | 0.2 |
|  | 5 | 1,519 | 99.4 (98.8-99.8) | 0.2 | 333 | 98.2 (96.0-99.3) | 0.8 | 1,186 | 99.8 (99.2-100.0) | 0.2 |
|  | 6 | 1,486 | 99.4 (98.8-99.8) | 0.2 | 331 | 98.3 (96.0-99.4) | 0.8 | 1,155 | 99.7 (99.1-100.0) | 0.2 |
|  | 7 | 1,433 | 99.5 (98.8-99.8) | 0.2 | 328 | 98.4 (96.1-99.5) | 0.8 | 1,105 | 99.8 (99.1-100.1) | 0.2 |
|  | 8 | 1,389 | 99.3 (98.6-99.7) | 0.3 | 322 | 97.8 (95.3-99.1) | 0.9 | 1,067 | 99.8 (99.1-100.1) | 0.2 |
|  | 9 | 1,350 | 99.2 (98.5-99.7) | 0.3 | 318 | 97.9 (95.4-99.2) | 0.9 | 1,032 | 99.6 (98.8-100.0) | 0.3 |
|  | 10 | 1,296 | 99.1 (98.3-99.7) | 0.3 | 302 | 97.7 (95.0-99.1) | 1.0 | 994 | 99.6 (98.8-100.0) | 0.3 |
|  | 11 | 1,269 | 99.2 (98.4-99.8) | 0.3 | 290 | 97.8 (95.2-99.2) | 1.0 | 979 | 99.7 (98.8-100.1) | 0.3 |
|  | 12 | 1,214 | 99.2 (98.3-99.7) | 0.3 | 270 | 97.9 (95.3-99.4) | 1.0 | 944 | 99.5 (98.6-100.1) | 0.3 |
|  | 13 | 1,202 | 99.2 (98.3-99.8) | 0.4 | 270 | 97.7 (94.9-99.2) | 1.1 | 932 | 99.7 (98.7-100.2) | 0.3 |
|  | 14 | 1,178 | 99.1 (98.1-99.7) | 0.4 | 259 | 97.4 (94.5-99.1) | 1.1 | 919 | 99.5 (98.5-100.1) | 0.4 |
|  | 15 | 1,155 | 99.0 (98.0-99.7) | 0.4 | 258 | 97.2 (94.1-99.0) | 1.2 | 897 | 99.5 (98.5-100.2) | 0.4 |
| Carcinoma of head and neck |  |  |  |  |  |  |  |  |  |  |
|  | 1 | 544 | 94.4 (92.1-96.0) | 1.0 | 300 | 92.5 (88.8-94.9) | 1.5 | 244 | 96.8 (93.6-98.4) | 1.1 |
|  | 2 | 570 | 90.9 (88.2-93.1) | 1.2 | 307 | 89.1 (85.0-92.2) | 1.8 | 263 | 93.1 (89.1-95.7) | 1.6 |
|  | 3 | 537 | 88.9 (85.9-91.3) | 1.4 | 295 | 86.9 (82.5-90.2) | 2.0 | 242 | 91.4 (87.1-94.4) | 1.8 |
|  | 4 | 531 | 87.3 (84.2-89.8) | 1.4 | 296 | 85.3 (80.7-88.9) | 2.1 | 235 | 89.8 (85.1-93.0) | 2.0 |
|  | 5 | 533 | 86.8 (83.6-89.4) | 1.5 | 295 | 85.0 (80.4-88.7) | 2.1 | 238 | 89.0 (84.2-92.4) | 2.0 |
|  | 6 | 543 | 85.6 (82.3-88.3) | 1.5 | 300 | 83.5 (78.8-87.3) | 2.2 | 243 | 88.2 (83.4-91.7) | 2.1 |
|  | 7 | 530 | 85.2 (81.9-87.9) | 1.5 | 298 | 83.3 (78.5-87.1) | 2.2 | 232 | 87.5 (82.5-91.1) | 2.2 |
|  | 8 | 534 | 84.9 (81.6-87.7) | 1.6 | 298 | 82.8 (78.0-86.6) | 2.2 | 236 | 87.5 (82.6-91.2) | 2.2 |
|  | 9 | 548 | 84.5 (81.1-87.3) | 1.6 | 303 | 82.3 (77.4-86.2) | 2.2 | 245 | 87.2 (82.2-90.9) | 2.2 |
|  | 10 | 543 | 83.7 (80.3-86.6) | 1.6 | 304 | 81.4 (76.5-85.5) | 2.3 | 239 | 86.5 (81.4-90.3) | 2.2 |
|  | 11 | 528 | 82.4 (78.9-85.4) | 1.7 | 295 | 80.1 (75.1-84.2) | 2.3 | 233 | 85.3 (80.1-89.3) | 2.3 |
|  | 12 | 532 | 81.7 (78.1-84.7) | 1.7 | 305 | 79.0 (74.0-83.3) | 2.4 | 227 | 85.0 (79.7-89.0) | 2.4 |
|  | 13 | 521 | 81.4 (77.8-84.5) | 1.7 | 300 | 78.9 (73.8-83.2) | 2.4 | 221 | 84.7 (79.4-88.8) | 2.4 |
|  | 14 | 515 | 81.4 (77.8-84.5) | 1.7 | 295 | 78.7 (73.6-83.0) | 2.4 | 220 | 84.8 (79.5-88.9) | 2.4 |
|  | 15 | 508 | 80.1 (76.4-83.3) | 1.8 | 288 | 76.8 (71.6-81.3) | 2.5 | 220 | 84.5 (79.1-88.6) | 2.4 |
| Carcinoma of gastrointestinal tract |  |  |  |  |  |  |  |  |  |  |
| Carcinoma of esophagus | 1 | 91 | 63.9 (53.2-72.7) | 5.0 | 64 | 61.1 (48.2-71.7) | 6.0 | 27 | 70.5 (49.6-84.0) | 8.7 |
|  | 2 | 67 | 49.6 (39.1-59.3) | 5.2 | 47 | 47.7 (35.4-59.1) | 6.1 | 20 | 54.0 (33.3-70.8) | 9.8 |
|  | 3 | 49 | 35.2 (25.6-44.9) | 5.0 | 38 | 32.1 (21.5-43.2) | 5.6 | 11 | 44.4 (24.4-62.7) | 10.2 |
|  | 4 | 36 | 30.9 (21.8-40.5) | 4.8 | 27 | 28.1 (18.1-38.9) | 5.4 | 9 | 39.2 (19.9-58.1) | 10.2 |
|  | 5 | 30 | 29.8 (20.8-39.3) | 4.8 | 22 | 26.8 (17.1-37.5) | 5.3 | 8 | 39.3 (19.9-58.2) | 10.2 |
|  | 6 | 31 | 27.8 (19.0-37.1) | 4.7 | 22 | 24.1 (14.9-34.6) | 5.1 | 9 | 39.3 (19.9-58.2) | 10.2 |
|  | 7 | 29 | 25.7 (17.3-34.9) | 4.6 | 20 | 22.8 (13.9-33.1) | 5.0 | 9 | 34.8 (16.5-53.9) | 10.0 |
|  | 8 | 28 | 25.7 (17.3-35.0) | 4.6 | 19 | 22.8 (13.9-33.2) | 5.0 | 9 | 34.8 (16.5-53.9) | 10.0 |
|  | 9 | 28 | 25.8 (17.4-35.0) | 4.6 | 20 | 22.9 (13.9-33.2) | 5.0 | 8 | 34.9 (16.6-54.0) | 10.0 |
|  | 10 | 28 | 24.8 (16.5-33.9) | 4.5 | 20 | 21.7 (13.0-31.8) | 4.9 | 8 | 34.9 (16.6-54.0) | 10.0 |
|  | 11 | 28 | 24.8 (16.5-34.0) | 4.5 | 21 | 21.7 (13.0-31.9) | 4.9 | 7 | 34.9 (16.6-54.1) | 10.0 |
|  | 12 | 27 | 23.9 (15.8-33.0) | 4.4 | 19 | 20.6 (12.1-30.6) | 4.8 | 8 | 35.0 (16.6-54.2) | 10.1 |
|  | 13 | 26 | 24.0 (15.8-33.1) | 4.5 | 17 | 20.6 (12.2-30.7) | 4.8 | 9 | 35.1 (16.7-54.3) | 10.1 |
|  | 14 | 24 | 24.0 (15.9-33.1) | 4.5 | 15 | 20.7 (12.2-30.7) | 4.8 | 9 | 35.1 (16.7-54.4) | 10.1 |
|  | 15 | 22 | 24.1 (15.9-33.2) | 4.5 | 13 | 20.7 (12.2-30.8) | 4.8 | 9 | 35.2 (16.7-54.5) | 10.1 |
| Carcinoma of stomach | 1 | 268 | 58.4 (52.4-64.0) | 3.0 | 142 | 59.2 (50.7-66.8) | 4.1 | 126 | 57.6 (48.6-65.5) | 4.3 |
|  | 2 | 165 | 43.2 (37.2-49.0) | 3.0 | 88 | 43.9 (35.6-51.9) | 4.2 | 77 | 42.3 (33.6-50.8) | 4.4 |
|  | 3 | 118 | 37.5 (31.6-43.3) | 3.0 | 65 | 40.2 (32.0-48.2) | 4.2 | 53 | 34.3 (26.0-42.7) | 4.3 |
|  | 4 | 103 | 35.9 (30.1-41.7) | 3.0 | 57 | 38.7 (30.6-46.7) | 4.1 | 46 | 32.6 (24.5-41.0) | 4.3 |
|  | 5 | 104 | 33.6 (27.9-39.4) | 2.9 | 57 | 36.5 (28.5-44.5) | 4.1 | 47 | 30.2 (22.3-38.5) | 4.2 |
|  | 6 | 94 | 32.4 (26.8-38.2) | 2.9 | 52 | 34.2 (26.4-42.2) | 4.1 | 42 | 30.2 (22.3-38.5) | 4.2 |
|  | 7 | 89 | 30.5 (25.0-36.2) | 2.9 | 50 | 32.8 (25.1-40.7) | 4.0 | 39 | 27.7 (20.1-35.9) | 4.1 |
|  | 8 | 88 | 29.8 (24.3-35.4) | 2.8 | 51 | 32.1 (24.5-40.0) | 4.0 | 37 | 27.0 (19.4-35.1) | 4.0 |
|  | 9 | 82 | 29.4 (24.0-35.1) | 2.8 | 47 | 31.5 (23.9-39.3) | 4.0 | 35 | 27.0 (19.4-35.1) | 4.0 |
|  | 10 | 82 | 29.5 (24.0-35.1) | 2.8 | 46 | 31.5 (23.9-39.4) | 4.0 | 36 | 27.0 (19.4-35.1) | 4.0 |
|  | 11 | 79 | 29.1 (23.7-34.7) | 2.8 | 43 | 30.8 (23.2-38.6) | 4.0 | 36 | 27.0 (19.5-35.2) | 4.0 |
|  | 12 | 73 | 28.3 (22.9-33.9) | 2.8 | 41 | 29.2 (21.8-37.0) | 3.9 | 32 | 27.1 (19.5-35.2) | 4.1 |
|  | 13 | 71 | 28.3 (22.9-33.9) | 2.8 | 41 | 29.3 (21.8-37.1) | 3.9 | 30 | 27.1 (19.5-35.2) | 4.1 |
|  | 14 | 67 | 27.9 (22.5-33.5) | 2.8 | 39 | 28.5 (21.1-36.3) | 3.9 | 28 | 27.1 (19.5-35.3) | 4.1 |
|  | 15 | 66 | 27.5 (22.1-33.1) | 2.8 | 40 | 27.8 (20.4-35.6) | 3.9 | 26 | 27.2 (19.6-35.3) | 4.1 |
| Carcinoma of small intestine | 1 | 33 | 84.6 (66.8-93.3) | 6.4 | 16 | 93.2 (60.3-99.1) | 6.6 | 17 | 77.6 (50.9-91.0) | 9.8 |
|  | 2 | 28 | 68.8 (49.7-81.9) | 8.2 | 14 | 71.9 (41.4-88.5) | 11.9 | 14 | 66.2 (39.7-83.2) | 11.2 |
|  | 3 | 23 | 55.8 (37.0-71.0) | 8.9 | 11 | 65.3 (35.6-83.9) | 12.6 | 12 | 47.1 (22.8-68.2) | 12.2 |
|  | 4 | 18 | 49.5 (31.3-65.4) | 8.9 | 10 | 58.8 (30.3-79.0) | 12.9 | 8 | 41.1 (18.2-62.8) | 12.1 |
|  | 5 | 16 | 49.6 (31.3-65.4) | 8.9 | 9 | 58.8 (30.4-79.1) | 12.9 | 7 | 41.1 (18.3-62.8) | 12.1 |
|  | 6 | 16 | 49.6 (31.4-65.5) | 8.9 | 9 | 58.9 (30.4-79.1) | 12.9 | 7 | 41.1 (18.3-62.9) | 12.1 |
|  | 7 | 15 | 49.6 (31.4-65.5) | 8.9 | 8 | 58.9 (30.4-79.2) | 12.9 | 7 | 41.1 (18.3-62.9) | 12.1 |
|  | 8 | 12 | 49.7 (31.4-65.6) | 8.9 | 8 | 59.0 (30.4-79.3) | 13.0 | 4 | 41.2 (18.3-63.0) | 12.1 |
|  | 9 | 14 | 49.7 (31.5-65.7) | 9.0 | 8 | 59.1 (30.5-79.4) | 13.0 | 6 | 41.2 (18.3-63.0) | 12.1 |
|  | 10 | 14 | 49.8 (31.5-65.7) | 9.0 | 7 | 59.1 (30.5-79.5) | 13.0 | 7 | 41.2 (18.3-63.1) | 12.1 |
|  | 11 | 12 | 49.9 (31.5-65.8) | 9.0 | 6 | 59.2 (30.6-79.6) | 13.0 | 6 | 41.3 (18.3-63.1) | 12.2 |
|  | 12 | 10 | 49.9 (31.6-65.9) | 9.0 | 5 | 59.4 (30.6-79.8) | 13.0 | 5 | 41.3 (18.4-63.2) | 12.2 |
|  | 13 | 11 | 50.0 (31.6-66.1) | 9.0 | 5 | 59.5 (30.7-80.0) | 13.1 | 6 | 41.4 (18.4-63.3) | 12.2 |
|  | 14 | 11 | 50.1 (31.7-66.2) | 9.0 | 5 | 59.7 (30.8-80.2) | 13.1 | 6 | 41.4 (18.4-63.4) | 12.2 |
|  | 15 | 11 | 50.2 (31.8-66.3) | 9.1 | 5 | 59.8 (30.9-80.4) | 13.1 | 6 | 41.5 (18.4-63.5) | 12.2 |
| Carcinoma of colon | 1 | 764 | 86.6 (84.0-88.9) | 1.2 | 366 | 85.7 (81.6-88.9) | 1.8 | 398 | 87.5 (83.8-90.4) | 1.7 |
|  | 2 | 719 | 77.3 (74.1-80.1) | 1.5 | 346 | 75.8 (71.1-79.9) | 2.3 | 373 | 78.7 (74.3-82.4) | 2.1 |
|  | 3 | 628 | 72.5 (69.1-75.6) | 1.6 | 303 | 71.6 (66.6-75.9) | 2.4 | 325 | 73.4 (68.7-77.5) | 2.3 |
|  | 4 | 590 | 69.1 (65.7-72.3) | 1.7 | 288 | 68.6 (63.5-73.1) | 2.5 | 302 | 69.6 (64.7-74.0) | 2.4 |
|  | 5 | 555 | 67.3 (63.8-70.5) | 1.7 | 273 | 66.5 (61.3-71.1) | 2.5 | 282 | 68.0 (63.1-72.5) | 2.4 |
|  | 6 | 544 | 65.7 (62.2-69.0) | 1.7 | 264 | 65.7 (60.5-70.4) | 2.5 | 280 | 65.7 (60.7-70.2) | 2.4 |
|  | 7 | 520 | 64.9 (61.4-68.3) | 1.8 | 253 | 64.9 (59.7-69.7) | 2.5 | 267 | 64.9 (59.8-69.5) | 2.5 |
|  | 8 | 494 | 64.7 (61.1-68.1) | 1.8 | 244 | 64.7 (59.5-69.5) | 2.5 | 250 | 64.7 (59.6-69.3) | 2.5 |
|  | 9 | 512 | 64.2 (60.6-67.6) | 1.8 | 254 | 64.5 (59.3-69.3) | 2.5 | 258 | 63.9 (58.8-68.5) | 2.5 |
|  | 10 | 517 | 63.8 (60.1-67.1) | 1.8 | 268 | 64.4 (59.1-69.1) | 2.6 | 249 | 63.1 (58.0-67.8) | 2.5 |
|  | 11 | 513 | 63.7 (60.1-67.1) | 1.8 | 267 | 64.2 (58.9-69.0) | 2.6 | 246 | 63.2 (58.1-67.9) | 2.5 |
|  | 12 | 508 | 63.1 (59.5-66.5) | 1.8 | 261 | 63.2 (57.9-68.0) | 2.6 | 247 | 63.0 (57.8-67.7) | 2.5 |
|  | 13 | 492 | 63.1 (59.4-66.5) | 1.8 | 247 | 63.3 (58.0-68.1) | 2.6 | 245 | 62.8 (57.6-67.5) | 2.5 |
|  | 14 | 472 | 62.7 (59.1-66.2) | 1.8 | 238 | 62.9 (57.5-67.7) | 2.6 | 234 | 62.6 (57.4-67.3) | 2.5 |
|  | 15 | 437 | 62.7 (59.0-66.2) | 1.8 | 219 | 63.0 (57.7-67.9) | 2.6 | 218 | 62.4 (57.2-67.1) | 2.5 |
| Carcinoma of rectum | 1 | 439 | 91.7 (88.7-93.9) | 1.3 | 245 | 92.4 (88.3-95.1) | 1.7 | 194 | 90.9 (85.9-94.2) | 2.1 |
|  | 2 | 442 | 83.0 (79.2-86.3) | 1.8 | 244 | 83.8 (78.5-87.9) | 2.4 | 198 | 82.1 (75.9-86.8) | 2.8 |
|  | 3 | 392 | 76.3 (72.0-80.0) | 2.0 | 224 | 78.9 (73.2-83.5) | 2.6 | 168 | 73.0 (66.1-78.7) | 3.2 |
|  | 4 | 361 | 70.3 (65.8-74.4) | 2.2 | 207 | 72.7 (66.6-77.9) | 2.9 | 154 | 67.3 (60.2-73.5) | 3.4 |
|  | 5 | 350 | 65.9 (61.2-70.2) | 2.3 | 199 | 67.5 (61.2-73.0) | 3.0 | 151 | 63.8 (56.6-70.2) | 3.5 |
|  | 6 | 329 | 62.2 (57.5-66.6) | 2.3 | 182 | 62.7 (56.3-68.5) | 3.1 | 147 | 61.5 (54.2-67.9) | 3.5 |
|  | 7 | 301 | 60.1 (55.3-64.5) | 2.3 | 167 | 59.9 (53.5-65.8) | 3.2 | 134 | 60.1 (52.8-66.6) | 3.5 |
|  | 8 | 308 | 59.7 (54.9-64.1) | 2.4 | 168 | 59.2 (52.8-65.1) | 3.2 | 140 | 60.1 (52.8-66.6) | 3.5 |
|  | 9 | 317 | 58.7 (53.9-63.2) | 2.4 | 178 | 57.8 (51.3-63.7) | 3.2 | 139 | 59.7 (52.4-66.3) | 3.5 |
|  | 10 | 309 | 57.8 (53.0-62.2) | 2.4 | 161 | 56.7 (50.3-62.7) | 3.2 | 148 | 58.9 (51.6-65.5) | 3.5 |
|  | 11 | 299 | 57.6 (52.8-62.1) | 2.4 | 154 | 56.4 (49.9-62.4) | 3.2 | 145 | 58.9 (51.7-65.5) | 3.5 |
|  | 12 | 281 | 56.4 (51.6-60.9) | 2.4 | 143 | 55.7 (49.2-61.7) | 3.2 | 138 | 57.1 (49.8-63.8) | 3.6 |
|  | 13 | 258 | 55.1 (50.3-59.6) | 2.4 | 135 | 53.5 (47.0-59.7) | 3.2 | 123 | 56.7 (49.4-63.4) | 3.6 |
|  | 14 | 249 | 54.9 (50.1-59.5) | 2.4 | 124 | 53.7 (47.1-59.8) | 3.2 | 125 | 56.3 (49.0-63.0) | 3.6 |
|  | 15 | 240 | 54.8 (50.0-59.4) | 2.4 | 119 | 53.8 (47.2-59.9) | 3.2 | 121 | 55.9 (48.6-62.6) | 3.6 |
| Carcinoma of anus | 1 | 56 | 96.5 (86.4-99.1) | 2.5 | 30 | 93.3 (75.7-98.3) | 4.6 | 26 | 100.0 (100.0-100.0) | 0.0 |
|  | 2 | 57 | 91.2 (80.1-96.3) | 3.8 | 29 | 90.1 (72.0-96.8) | 5.5 | 28 | 92.6 (73.4-98.2) | 5.1 |
|  | 3 | 54 | 87.8 (76.0-94.1) | 4.4 | 29 | 86.9 (68.6-95.0) | 6.2 | 25 | 88.9 (69.2-96.4) | 6.1 |
|  | 4 | 55 | 86.2 (74.1-93.0) | 4.6 | 30 | 83.9 (65.4-93.1) | 6.7 | 25 | 89.0 (69.2-96.4) | 6.1 |
|  | 5 | 55 | 79.4 (66.4-87.8) | 5.4 | 29 | 74.3 (54.9-86.4) | 7.9 | 26 | 85.4 (65.2-94.4) | 6.9 |
|  | 6 | 51 | 77.7 (64.5-86.5) | 5.5 | 26 | 71.1 (51.6-84.0) | 8.2 | 25 | 85.4 (65.3-94.5) | 6.9 |
|  | 7 | 47 | 77.8 (64.6-86.6) | 5.6 | 26 | 71.2 (51.7-84.1) | 8.2 | 21 | 85.5 (65.4-94.6) | 6.9 |
|  | 8 | 43 | 75.8 (62.3-85.1) | 5.8 | 23 | 71.3 (51.7-84.2) | 8.2 | 20 | 80.4 (58.2-91.8) | 8.2 |
|  | 9 | 39 | 75.9 (62.4-85.2) | 5.8 | 22 | 71.4 (51.8-84.3) | 8.3 | 17 | 80.5 (58.2-91.8) | 8.2 |
|  | 10 | 40 | 76.0 (62.4-85.3) | 5.8 | 21 | 71.5 (51.9-84.4) | 8.3 | 19 | 80.6 (58.3-92.0) | 8.2 |
|  | 11 | 39 | 76.1 (62.5-85.5) | 5.8 | 19 | 71.6 (52.0-84.5) | 8.3 | 20 | 80.7 (58.4-92.1) | 8.2 |
|  | 12 | 38 | 76.2 (62.6-85.6) | 5.8 | 17 | 71.7 (52.1-84.7) | 8.3 | 21 | 80.8 (58.5-92.2) | 8.2 |
|  | 13 | 37 | 76.4 (62.7-85.8) | 5.8 | 18 | 71.9 (52.2-84.9) | 8.3 | 19 | 80.9 (58.6-92.3) | 8.3 |
|  | 14 | 36 | 76.5 (62.9-85.9) | 5.8 | 17 | 72.0 (52.3-85.0) | 8.3 | 19 | 81.0 (58.7-92.5) | 8.3 |
|  | 15 | 34 | 71.7 (57.1-82.3) | 6.4 | 16 | 67.4 (46.6-81.9) | 9.1 | 18 | 76.1 (52.8-89.5) | 9.2 |
| Carcinoma of liver and intrahepatic bile ducts (IBD) | 1 | 111 | 57.7 (47.8-66.3) | 4.7 | 62 | 52.2 (38.8-64.1) | 6.5 | 49 | 63.8 (49.0-75.4) | 6.8 |
|  | 2 | 67 | 47.5 (37.8-56.5) | 4.8 | 33 | 47.1 (33.9-59.2) | 6.5 | 34 | 47.9 (33.6-60.8) | 7.0 |
|  | 3 | 55 | 46.5 (36.9-55.6) | 4.8 | 30 | 45.3 (32.3-57.5) | 6.5 | 25 | 47.9 (33.7-60.8) | 7.0 |
|  | 4 | 49 | 41.1 (31.6-50.3) | 4.8 | 25 | 39.1 (26.4-51.6) | 6.6 | 24 | 43.3 (29.3-56.5) | 7.1 |
|  | 5 | 41 | 39.9 (30.5-49.2) | 4.8 | 21 | 39.2 (26.5-51.7) | 6.6 | 20 | 40.7 (26.9-54.2) | 7.1 |
|  | 6 | 37 | 38.9 (29.5-48.2) | 4.8 | 18 | 37.0 (24.5-49.6) | 6.5 | 19 | 40.8 (26.9-54.2) | 7.1 |
|  | 7 | 38 | 37.7 (28.4-47.1) | 4.8 | 19 | 37.1 (24.5-49.7) | 6.6 | 19 | 38.2 (24.5-51.8) | 7.1 |
|  | 8 | 32 | 37.8 (28.4-47.1) | 4.8 | 18 | 37.1 (24.5-49.7) | 6.6 | 14 | 38.2 (24.5-51.8) | 7.1 |
|  | 9 | 25 | 37.8 (28.4-47.1) | 4.8 | 14 | 37.2 (24.6-49.8) | 6.6 | 11 | 38.3 (24.6-51.9) | 7.1 |
|  | 10 | 25 | 37.8 (28.5-47.2) | 4.8 | 13 | 37.2 (24.6-49.8) | 6.6 | 12 | 38.3 (24.6-51.9) | 7.1 |
|  | 11 | 21 | 37.9 (28.5-47.2) | 4.8 | 12 | 37.2 (24.6-49.9) | 6.6 | 9 | 38.3 (24.6-51.9) | 7.1 |
|  | 12 | 17 | 37.9 (28.5-47.3) | 4.8 | 7 | 37.3 (24.7-50.0) | 6.6 | 10 | 38.4 (24.6-52.0) | 7.1 |
|  | 13 | 17 | 33.5 (23.6-43.7) | 5.2 | 6 | 31.4 (17.2-46.7) | 7.8 | 11 | 34.8 (21.1-49.0) | 7.3 |
|  | 14 | 15 | 33.6 (23.6-43.8) | 5.2 | 6 | 31.4 (17.2-46.8) | 7.8 | 9 | 34.9 (21.1-49.1) | 7.3 |
|  | 15 | 13 | 33.6 (23.7-43.8) | 5.2 | 6 | 31.5 (17.2-46.9) | 7.8 | 7 | 34.9 (21.1-49.1) | 7.3 |
| Carcinoma of gallbladder and other extrahepatic biliary | 1 | 75 | 71.4 (59.9-80.1) | 5.1 | 43 | 71.2 (55.7-82.1) | 6.7 | 32 | 71.6 (52.6-84.0) | 8.0 |
|  | 2 | 55 | 43.5 (31.9-54.5) | 5.9 | 31 | 43.7 (28.0-58.3) | 7.9 | 24 | 43.2 (26.1-59.3) | 8.7 |
|  | 3 | 32 | 33.2 (22.5-44.2) | 5.6 | 17 | 35.7 (21.2-50.5) | 7.7 | 15 | 30.1 (15.4-46.3) | 8.2 |
|  | 4 | 26 | 31.8 (21.3-42.7) | 5.6 | 14 | 33.0 (19.0-47.8) | 7.6 | 12 | 30.1 (15.4-46.3) | 8.2 |
|  | 5 | 25 | 30.4 (20.1-41.3) | 5.5 | 14 | 30.5 (17.0-45.2) | 7.4 | 11 | 30.1 (15.5-46.3) | 8.2 |
|  | 6 | 21 | 28.8 (18.8-39.7) | 5.4 | 13 | 27.9 (14.9-42.4) | 7.2 | 8 | 30.2 (15.5-46.3) | 8.2 |
|  | 7 | 19 | 28.9 (18.8-39.7) | 5.4 | 10 | 27.9 (14.9-42.5) | 7.2 | 9 | 30.2 (15.5-46.4) | 8.2 |
|  | 8 | 19 | 28.9 (18.8-39.8) | 5.4 | 9 | 27.9 (14.9-42.5) | 7.3 | 10 | 30.2 (15.5-46.4) | 8.2 |
|  | 9 | 21 | 28.9 (18.8-39.8) | 5.4 | 10 | 28.0 (15.0-42.6) | 7.3 | 11 | 30.2 (15.5-46.4) | 8.2 |
|  | 10 | 18 | 29.0 (18.8-39.8) | 5.4 | 8 | 28.0 (15.0-42.6) | 7.3 | 10 | 30.3 (15.5-46.5) | 8.2 |
|  | 11 | 19 | 27.3 (17.4-38.1) | 5.4 | 9 | 24.7 (12.3-39.4) | 7.1 | 10 | 30.3 (15.5-46.5) | 8.2 |
|  | 12 | 19 | 27.3 (17.4-38.2) | 5.4 | 9 | 24.7 (12.3-39.4) | 7.2 | 10 | 30.3 (15.5-46.6) | 8.2 |
|  | 13 | 20 | 27.3 (17.4-38.2) | 5.4 | 10 | 24.8 (12.3-39.5) | 7.2 | 10 | 30.4 (15.6-46.6) | 8.2 |
|  | 14 | 19 | 25.8 (16.1-36.6) | 5.3 | 9 | 21.9 (10.3-36.4) | 6.9 | 10 | 30.4 (15.6-46.7) | 8.2 |
|  | 15 | 15 | 25.8 (16.1-36.7) | 5.3 | 8 | 22.0 (10.3-36.5) | 6.9 | 7 | 30.5 (15.6-46.8) | 8.3 |
| Carcinoma of pancreas | 1 | 90 | 52.6 (42.2-62.0) | 5.1 | 44 | 53.7 (38.6-66.7) | 7.3 | 46 | 51.6 (36.9-64.4) | 7.1 |
|  | 2 | 51 | 39.5 (29.5-49.4) | 5.1 | 26 | 39.8 (25.7-53.6) | 7.3 | 25 | 39.4 (25.4-53.1) | 7.2 |
|  | 3 | 33 | 34.5 (24.8-44.4) | 5.1 | 15 | 29.4 (16.7-43.3) | 7.0 | 18 | 39.4 (25.4-53.1) | 7.2 |
|  | 4 | 30 | 32.1 (22.6-41.9) | 5.0 | 12 | 23.9 (12.2-37.7) | 6.7 | 18 | 39.4 (25.4-53.1) | 7.2 |
|  | 5 | 30 | 30.9 (21.6-40.7) | 4.9 | 9 | 23.9 (12.2-37.7) | 6.7 | 21 | 37.2 (23.5-50.9) | 7.2 |
|  | 6 | 27 | 30.9 (21.6-40.7) | 5.0 | 9 | 23.9 (12.2-37.7) | 6.7 | 18 | 37.2 (23.5-51.0) | 7.2 |
|  | 7 | 20 | 31.0 (21.6-40.8) | 5.0 | 6 | 23.9 (12.2-37.7) | 6.7 | 14 | 37.3 (23.6-51.0) | 7.2 |
|  | 8 | 17 | 29.2 (19.8-39.1) | 5.0 | 5 | 23.9 (12.3-37.8) | 6.7 | 12 | 34.2 (20.7-48.2) | 7.2 |
|  | 9 | 16 | 29.2 (19.9-39.1) | 5.0 | 6 | 24.0 (12.3-37.8) | 6.7 | 10 | 34.2 (20.7-48.2) | 7.2 |
|  | 10 | 16 | 29.2 (19.9-39.2) | 5.0 | 5 | 24.0 (12.3-37.9) | 6.7 | 11 | 34.2 (20.7-48.2) | 7.2 |
|  | 11 | 15 | 29.2 (19.9-39.2) | 5.0 | 5 | 24.0 (12.3-37.9) | 6.7 | 10 | 34.3 (20.7-48.3) | 7.2 |
|  | 12 | 15 | 29.3 (19.9-39.3) | 5.0 | 4 | 24.1 (12.3-38.0) | 6.7 | 11 | 34.3 (20.7-48.3) | 7.2 |
|  | 13 | 12 | 29.3 (19.9-39.3) | 5.0 | 2 | 24.1 (12.3-38.1) | 6.8 | 10 | 34.3 (20.7-48.4) | 7.2 |
|  | 14 | 10 | 29.4 (20.0-39.4) | 5.0 | 2 | 24.2 (12.4-38.2) | 6.8 | 8 | 34.4 (20.8-48.5) | 7.3 |
|  | 15 | 10 | 29.4 (20.0-39.5) | 5.0 | 2 | 24.2 (12.4-38.2) | 6.8 | 8 | 34.5 (20.8-48.6) | 7.3 |
| Other carcinoma of gastrointestinal tract | 1 | 5 | 18.8 (1.2-53.5) | 15.7 | 1 | 25.3 (0.0-82.5) | 34.8 | 4 | 16.6 (0.4-56.0) | 17.2 |
|  | 2 | 1 | 18.8 (1.2-53.5) | 15.7 | NA | NA | NA | 1 | 16.6 (0.4-56.0) | 17.2 |
|  | 3 | 1 | 18.9 (1.2-53.5) | 15.7 | NA | NA | NA | 1 | 16.6 (0.4-56.0) | 17.2 |
|  | 4 | 1 | 18.9 (1.2-53.5) | 15.7 | NA | NA | NA | 1 | 16.6 (0.4-56.0) | 17.2 |
|  | 5 | 1 | 18.9 (1.2-53.5) | 15.7 | NA | NA | NA | 1 | 16.6 (0.4-56.1) | 17.2 |
|  | 6 | 1 | 18.9 (1.2-53.5) | 15.8 | NA | NA | NA | 1 | 16.6 (0.4-56.1) | 17.2 |
|  | 7 | NA | NA | NA | NA | NA | NA | NA | NA | NA |
|  | 8 | NA | NA | NA | NA | NA | NA | NA | NA | NA |
|  | 9 | NA | NA | NA | NA | NA | NA | NA | NA | NA |
|  | 10 | NA | NA | NA | NA | NA | NA | NA | NA | NA |
|  | 11 | NA | NA | NA | NA | NA | NA | NA | NA | NA |
|  | 12 | NA | NA | NA | NA | NA | NA | NA | NA | NA |
|  | 13 | NA | NA | NA | NA | NA | NA | NA | NA | NA |
|  | 14 | NA | NA | NA | NA | NA | NA | NA | NA | NA |
|  | 15 | NA | NA | NA | NA | NA | NA | NA | NA | NA |
| Carcinoma of lung, bronchus, and trachea |  |  |  |  |  |  |  |  |  |  |
|  | 1 | 430 | 58.1 (53.4-62.6) | 2.4 | 233 | 55.7 (49.1-61.7) | 3.2 | 197 | 61.1 (53.9-67.5) | 3.5 |
|  | 2 | 268 | 42.5 (37.8-47.1) | 2.4 | 137 | 39.5 (33.2-45.8) | 3.2 | 131 | 45.9 (38.8-52.6) | 3.5 |
|  | 3 | 195 | 35.0 (30.5-39.5) | 2.3 | 94 | 33.0 (27.0-39.2) | 3.1 | 101 | 37.2 (30.5-43.9) | 3.4 |
|  | 4 | 163 | 30.9 (26.5-35.3) | 2.2 | 77 | 27.8 (22.0-33.8) | 3.0 | 86 | 34.3 (27.8-40.9) | 3.4 |
|  | 5 | 137 | 26.4 (22.3-30.7) | 2.2 | 62 | 24.3 (18.8-30.2) | 2.9 | 75 | 28.8 (22.7-35.2) | 3.2 |
|  | 6 | 112 | 23.7 (19.7-27.8) | 2.1 | 53 | 21.4 (16.2-27.1) | 2.8 | 59 | 26.2 (20.3-32.5) | 3.1 |
|  | 7 | 102 | 22.4 (18.5-26.5) | 2.1 | 50 | 20.9 (15.8-26.6) | 2.8 | 52 | 24.1 (18.4-30.3) | 3.1 |
|  | 8 | 91 | 21.6 (17.8-25.7) | 2.0 | 42 | 20.9 (15.8-26.6) | 2.8 | 49 | 22.5 (16.9-28.5) | 3.0 |
|  | 9 | 88 | 20.8 (17.0-24.9) | 2.0 | 44 | 19.9 (14.8-25.5) | 2.7 | 44 | 21.9 (16.4-28.0) | 3.0 |
|  | 10 | 81 | 20.6 (16.8-24.6) | 2.0 | 42 | 19.4 (14.4-25.0) | 2.7 | 39 | 22.0 (16.4-28.0) | 3.0 |
|  | 11 | 83 | 20.1 (16.3-24.1) | 2.0 | 41 | 18.4 (13.4-23.9) | 2.7 | 42 | 22.0 (16.5-28.1) | 3.0 |
|  | 12 | 73 | 19.2 (15.5-23.2) | 2.0 | 32 | 17.1 (12.3-22.6) | 2.6 | 41 | 21.4 (16.0-27.5) | 3.0 |
|  | 13 | 69 | 18.9 (15.2-22.9) | 2.0 | 28 | 17.1 (12.3-22.6) | 2.7 | 41 | 20.8 (15.4-26.9) | 2.9 |
|  | 14 | 66 | 18.9 (15.2-22.9) | 2.0 | 28 | 17.1 (12.3-22.7) | 2.7 | 38 | 20.9 (15.4-26.9) | 2.9 |
|  | 15 | 61 | 18.0 (14.3-22.0) | 2.0 | 26 | 16.5 (11.7-22.0) | 2.6 | 35 | 19.6 (14.3-25.6) | 2.9 |
| Carcinoma of skin (if collected)*** |  |  |  |  |  |  |  |  |  |  |
|  | 1 | 449 | 99.6 (98.3-99.9) | 0.3 | 199 | 99.1 (96.1-99.8) | 0.7 | 250 | 100.0 (100.0-100.0) | 0.0 |
|  | 2 | 498 | 98.8 (97.1-99.5) | 0.5 | 220 | 98.1 (94.8-99.4) | 1.0 | 278 | 99.3 (96.8-99.9) | 0.6 |
|  | 3 | 470 | 98.3 (96.5-99.2) | 0.6 | 213 | 97.7 (94.2-99.1) | 1.1 | 257 | 98.9 (96.3-99.7) | 0.7 |
|  | 4 | 454 | 97.9 (96.0-99.0) | 0.7 | 210 | 96.7 (92.9-98.6) | 1.3 | 244 | 98.9 (96.3-99.8) | 0.7 |
|  | 5 | 420 | 97.5 (95.4-98.7) | 0.8 | 188 | 96.8 (93.0-98.6) | 1.3 | 232 | 98.0 (95.0-99.3) | 1.0 |
|  | 6 | 396 | 97.6 (95.4-98.8) | 0.8 | 176 | 96.9 (93.0-98.7) | 1.3 | 220 | 98.1 (95.1-99.4) | 1.0 |
|  | 7 | 389 | 97.1 (94.8-98.5) | 0.9 | 179 | 97.0 (93.1-98.8) | 1.3 | 210 | 97.2 (93.7-98.8) | 1.2 |
|  | 8 | 368 | 96.3 (93.8-97.9) | 1.0 | 169 | 96.5 (92.4-98.6) | 1.5 | 199 | 96.2 (92.4-98.2) | 1.4 |
|  | 9 | 367 | 96.4 (93.9-98.0) | 1.0 | 171 | 96.6 (92.5-98.7) | 1.5 | 196 | 96.3 (92.5-98.3) | 1.4 |
|  | 10 | 384 | 96.0 (93.3-97.7) | 1.1 | 177 | 95.5 (91.0-98.0) | 1.7 | 207 | 96.4 (92.5-98.4) | 1.4 |
|  | 11 | 370 | 95.8 (93.1-97.6) | 1.1 | 172 | 95.6 (91.1-98.1) | 1.7 | 198 | 96.0 (92.0-98.2) | 1.5 |
|  | 12 | 362 | 95.4 (92.5-97.3) | 1.2 | 168 | 94.5 (89.7-97.4) | 1.9 | 194 | 96.1 (92.1-98.3) | 1.5 |
|  | 13 | 331 | 94.6 (91.5-96.7) | 1.3 | 154 | 93.4 (88.1-96.6) | 2.1 | 177 | 95.6 (91.4-98.0) | 1.6 |
|  | 14 | 316 | 93.8 (90.5-96.1) | 1.4 | 145 | 91.5 (85.6-95.2) | 2.4 | 171 | 95.8 (91.6-98.2) | 1.6 |
|  | 15 | 313 | 93.0 (89.5-95.5) | 1.5 | 133 | 89.5 (83.1-93.8) | 2.7 | 180 | 95.9 (91.7-98.4) | 1.6 |
| Carcinoma of breast |  |  |  |  |  |  |  |  |  |  |
|  | 1 | 7,090 | 98.9 (98.6-99.1) | 0.1 | 14 | 100.1 (100.1-100.1) | 0.0 | 7,076 | 98.9 (98.6-99.1) | 0.1 |
|  | 2 | 7,595 | 96.3 (95.9-96.7) | 0.2 | 14 | 100.1 (100.1-100.1) | 0.0 | 7,581 | 96.3 (95.8-96.7) | 0.2 |
|  | 3 | 7,254 | 94.2 (93.6-94.7) | 0.3 | 12 | 100.2 (100.2-100.2) | 0.0 | 7,242 | 94.2 (93.6-94.7) | 0.3 |
|  | 4 | 6,981 | 92.0 (91.3-92.6) | 0.3 | 11 | 100.3 (100.3-100.3) | 0.0 | 6,970 | 92.0 (91.3-92.6) | 0.3 |
|  | 5 | 6,670 | 90.1 (89.4-90.8) | 0.4 | 11 | 100.4 (100.4-100.4) | 0.0 | 6,659 | 90.1 (89.4-90.8) | 0.4 |
|  | 6 | 6,459 | 88.4 (87.6-89.1) | 0.4 | 9 | 100.5 (100.5-100.5) | 0.0 | 6,450 | 88.4 (87.6-89.1) | 0.4 |
|  | 7 | 6,280 | 87.1 (86.3-87.9) | 0.4 | 9 | 88.5 (40.5-98.8) | 11.3 | 6,271 | 87.1 (86.3-87.9) | 0.4 |
|  | 8 | 6,078 | 86.0 (85.1-86.8) | 0.4 | 8 | 88.6 (40.5-98.9) | 11.4 | 6,070 | 86.0 (85.1-86.8) | 0.4 |
|  | 9 | 5,935 | 85.0 (84.1-85.8) | 0.4 | 8 | 77.4 (35.0-94.4) | 14.5 | 5,927 | 85.0 (84.1-85.8) | 0.4 |
|  | 10 | 5,770 | 83.8 (82.9-84.7) | 0.5 | 9 | 77.5 (35.1-94.5) | 14.5 | 5,761 | 83.8 (82.9-84.7) | 0.5 |
|  | 11 | 5,700 | 82.8 (81.9-83.7) | 0.5 | 11 | 77.7 (35.1-94.7) | 14.5 | 5,689 | 82.8 (81.9-83.7) | 0.5 |
|  | 12 | 5,603 | 81.9 (80.9-82.8) | 0.5 | 11 | 77.8 (35.2-94.9) | 14.5 | 5,592 | 81.9 (80.9-82.8) | 0.5 |
|  | 13 | 5,421 | 81.2 (80.2-82.1) | 0.5 | 11 | 77.9 (35.3-95.0) | 14.6 | 5,410 | 81.2 (80.2-82.1) | 0.5 |
|  | 14 | 5,259 | 80.2 (79.2-81.2) | 0.5 | 10 | 78.1 (35.3-95.2) | 14.6 | 5,249 | 80.2 (79.2-81.2) | 0.5 |
|  | 15 | 5,049 | 79.1 (78.0-80.1) | 0.5 | 11 | 78.3 (35.4-95.4) | 14.6 | 5,038 | 79.0 (78.0-80.1) | 0.5 |
| Carcinoma of genital sites excluding ovary and testis |  |  |  |  |  |  |  |  |  |  |
| Carcinoma of uterine cervix | 1 | 2,462 | 97.9 (97.3-98.4) | 0.3 | NA | NA | NA | 2,462 | 97.9 (97.3-98.4) | 0.3 |
|  | 2 | 2,584 | 94.6 (93.6-95.4) | 0.5 | NA | NA | NA | 2,584 | 94.6 (93.6-95.4) | 0.5 |
|  | 3 | 2,349 | 92.6 (91.4-93.6) | 0.5 | NA | NA | NA | 2,349 | 92.6 (91.4-93.6) | 0.5 |
|  | 4 | 2,191 | 91.4 (90.2-92.5) | 0.6 | NA | NA | NA | 2,191 | 91.4 (90.2-92.5) | 0.6 |
|  | 5 | 2,073 | 90.6 (89.3-91.7) | 0.6 | NA | NA | NA | 2,073 | 90.6 (89.3-91.7) | 0.6 |
|  | 6 | 2,005 | 90.1 (88.8-91.3) | 0.6 | NA | NA | NA | 2,005 | 90.1 (88.8-91.3) | 0.6 |
|  | 7 | 1,926 | 89.5 (88.2-90.7) | 0.7 | NA | NA | NA | 1,926 | 89.5 (88.2-90.7) | 0.7 |
|  | 8 | 1,845 | 89.0 (87.5-90.2) | 0.7 | NA | NA | NA | 1,845 | 89.0 (87.5-90.2) | 0.7 |
|  | 9 | 1,806 | 88.8 (87.3-90.0) | 0.7 | NA | NA | NA | 1,806 | 88.8 (87.3-90.0) | 0.7 |
|  | 10 | 1,748 | 88.3 (86.9-89.6) | 0.7 | NA | NA | NA | 1,748 | 88.3 (86.9-89.6) | 0.7 |
|  | 11 | 1,705 | 87.9 (86.4-89.3) | 0.7 | NA | NA | NA | 1,705 | 87.9 (86.4-89.3) | 0.7 |
|  | 12 | 1,700 | 87.8 (86.3-89.1) | 0.7 | NA | NA | NA | 1,700 | 87.8 (86.3-89.1) | 0.7 |
|  | 13 | 1,689 | 87.5 (86.0-88.9) | 0.7 | NA | NA | NA | 1,689 | 87.5 (86.0-88.9) | 0.7 |
|  | 14 | 1,703 | 87.3 (85.7-88.7) | 0.8 | NA | NA | NA | 1,703 | 87.3 (85.7-88.7) | 0.8 |
|  | 15 | 1,717 | 87.0 (85.5-88.5) | 0.8 | NA | NA | NA | 1,717 | 87.0 (85.5-88.5) | 0.8 |
| Carcinoma of corpus uteri | 1 | 146 | 94.6 (89.4-97.3) | 1.9 | NA | NA | NA | 146 | 94.6 (89.4-97.3) | 1.9 |
|  | 2 | 147 | 92.4 (86.7-95.8) | 2.2 | NA | NA | NA | 147 | 92.4 (86.7-95.8) | 2.2 |
|  | 3 | 125 | 92.5 (86.7-95.8) | 2.2 | NA | NA | NA | 125 | 92.5 (86.7-95.8) | 2.2 |
|  | 4 | 107 | 92.5 (86.8-95.9) | 2.2 | NA | NA | NA | 107 | 92.5 (86.8-95.9) | 2.2 |
|  | 5 | 102 | 91.6 (85.5-95.3) | 2.4 | NA | NA | NA | 102 | 91.6 (85.5-95.3) | 2.4 |
|  | 6 | 96 | 89.5 (82.6-93.9) | 2.8 | NA | NA | NA | 96 | 89.5 (82.6-93.9) | 2.8 |
|  | 7 | 94 | 89.6 (82.6-93.9) | 2.8 | NA | NA | NA | 94 | 89.6 (82.6-93.9) | 2.8 |
|  | 8 | 93 | 89.7 (82.7-94.0) | 2.8 | NA | NA | NA | 93 | 89.7 (82.7-94.0) | 2.8 |
|  | 9 | 93 | 88.6 (81.3-93.3) | 3.0 | NA | NA | NA | 93 | 88.6 (81.3-93.3) | 3.0 |
|  | 10 | 83 | 88.7 (81.4-93.4) | 3.0 | NA | NA | NA | 83 | 88.7 (81.4-93.4) | 3.0 |
|  | 11 | 80 | 88.8 (81.5-93.5) | 3.0 | NA | NA | NA | 80 | 88.8 (81.5-93.5) | 3.0 |
|  | 12 | 82 | 86.5 (78.3-91.9) | 3.4 | NA | NA | NA | 82 | 86.5 (78.3-91.9) | 3.4 |
|  | 13 | 82 | 86.6 (78.5-92.0) | 3.4 | NA | NA | NA | 82 | 86.6 (78.5-92.0) | 3.4 |
|  | 14 | 91 | 86.8 (78.6-92.1) | 3.4 | NA | NA | NA | 91 | 86.8 (78.6-92.1) | 3.4 |
|  | 15 | 94 | 86.9 (78.7-92.3) | 3.4 | NA | NA | NA | 94 | 86.9 (78.7-92.3) | 3.4 |
| Carcinoma of vulva and vagina | 1 | 123 | 98.4 (93.7-99.6) | 1.1 | NA | NA | NA | 123 | 98.4 (93.7-99.6) | 1.1 |
|  | 2 | 136 | 94.4 (88.4-97.3) | 2.1 | NA | NA | NA | 136 | 94.4 (88.4-97.3) | 2.1 |
|  | 3 | 129 | 93.6 (87.5-96.8) | 2.2 | NA | NA | NA | 129 | 93.6 (87.5-96.8) | 2.2 |
|  | 4 | 127 | 92.8 (86.5-96.3) | 2.4 | NA | NA | NA | 127 | 92.8 (86.5-96.3) | 2.4 |
|  | 5 | 119 | 92.0 (85.5-95.7) | 2.5 | NA | NA | NA | 119 | 92.0 (85.5-95.7) | 2.5 |
|  | 6 | 116 | 91.2 (84.5-95.2) | 2.6 | NA | NA | NA | 116 | 91.2 (84.5-95.2) | 2.6 |
|  | 7 | 113 | 91.3 (84.6-95.3) | 2.6 | NA | NA | NA | 113 | 91.3 (84.6-95.3) | 2.6 |
|  | 8 | 110 | 91.4 (84.6-95.3) | 2.6 | NA | NA | NA | 110 | 91.4 (84.6-95.3) | 2.6 |
|  | 9 | 104 | 91.5 (84.7-95.4) | 2.6 | NA | NA | NA | 104 | 91.5 (84.7-95.4) | 2.6 |
|  | 10 | 97 | 90.5 (83.4-94.8) | 2.8 | NA | NA | NA | 97 | 90.5 (83.4-94.8) | 2.8 |
|  | 11 | 88 | 89.5 (82.1-94.1) | 3.0 | NA | NA | NA | 88 | 89.5 (82.1-94.1) | 3.0 |
|  | 12 | 90 | 89.6 (82.2-94.2) | 3.0 | NA | NA | NA | 90 | 89.6 (82.2-94.2) | 3.0 |
|  | 13 | 88 | 88.6 (80.8-93.5) | 3.2 | NA | NA | NA | 88 | 88.6 (80.8-93.5) | 3.2 |
|  | 14 | 81 | 88.8 (80.9-93.7) | 3.2 | NA | NA | NA | 81 | 88.8 (80.9-93.7) | 3.2 |
|  | 15 | 76 | 88.9 (81.1-93.9) | 3.2 | NA | NA | NA | 76 | 88.9 (81.1-93.9) | 3.2 |
| Carcinoma of penis | 1 | 14 | 92.9 (58.9-99.0) | 6.9 | 14 | 92.9 (58.9-99.0) | 6.9 | NA | NA | NA |
|  | 2 | 16 | 92.9 (59.0-99.1) | 6.9 | 16 | 92.9 (59.0-99.1) | 6.9 | NA | NA | NA |
|  | 3 | 17 | 93.0 (59.0-99.2) | 6.9 | 17 | 93.0 (59.0-99.2) | 6.9 | NA | NA | NA |
|  | 4 | 16 | 93.1 (59.1-99.3) | 6.9 | 16 | 93.1 (59.1-99.3) | 6.9 | NA | NA | NA |
|  | 5 | 20 | 93.2 (59.1-99.3) | 7.0 | 20 | 93.2 (59.1-99.3) | 7.0 | NA | NA | NA |
|  | 6 | 18 | 93.3 (59.2-99.4) | 7.0 | 18 | 93.3 (59.2-99.4) | 7.0 | NA | NA | NA |
|  | 7 | 21 | 93.4 (59.2-99.5) | 7.0 | 21 | 93.4 (59.2-99.5) | 7.0 | NA | NA | NA |
|  | 8 | 24 | 93.5 (59.3-99.7) | 7.0 | 24 | 93.5 (59.3-99.7) | 7.0 | NA | NA | NA |
|  | 9 | 25 | 93.6 (59.4-99.8) | 7.0 | 25 | 93.6 (59.4-99.8) | 7.0 | NA | NA | NA |
|  | 10 | 24 | 93.7 (59.5-99.9) | 7.0 | 24 | 93.7 (59.5-99.9) | 7.0 | NA | NA | NA |
|  | 11 | 24 | 93.9 (59.6-100.1) | 7.0 | 24 | 93.9 (59.6-100.1) | 7.0 | NA | NA | NA |
|  | 12 | 24 | 94.0 (59.7-100.3) | 7.0 | 24 | 94.0 (59.7-100.3) | 7.0 | NA | NA | NA |
|  | 13 | 21 | 94.2 (59.8-100.5) | 7.0 | 21 | 94.2 (59.8-100.5) | 7.0 | NA | NA | NA |
|  | 14 | 20 | 94.4 (59.9-100.7) | 7.0 | 20 | 94.4 (59.9-100.7) | 7.0 | NA | NA | NA |
|  | 15 | 19 | 94.7 (60.1-101.0) | 7.1 | 19 | 94.7 (60.1-101.0) | 7.1 | NA | NA | NA |
| Carcinoma of prostate | 1 | 8 | 100.1 (100.1-100.1) | 0.0 | 8 | 100.1 (100.1-100.1) | 0.0 | NA | NA | NA |
|  | 2 | 9 | 100.2 (100.2-100.2) | 0.0 | 9 | 100.2 (100.2-100.2) | 0.0 | NA | NA | NA |
|  | 3 | 10 | 100.3 (100.3-100.3) | 0.0 | 10 | 100.3 (100.3-100.3) | 0.0 | NA | NA | NA |
|  | 4 | 10 | 100.4 (100.4-100.4) | 0.0 | 10 | 100.4 (100.4-100.4) | 0.0 | NA | NA | NA |
|  | 5 | 10 | 100.5 (100.5-100.5) | 0.0 | 10 | 100.5 (100.5-100.5) | 0.0 | NA | NA | NA |
|  | 6 | 11 | 100.6 (100.6-100.6) | 0.0 | 11 | 100.6 (100.6-100.6) | 0.0 | NA | NA | NA |
|  | 7 | 12 | 100.7 (100.7-100.7) | 0.0 | 12 | 100.7 (100.7-100.7) | 0.0 | NA | NA | NA |
|  | 8 | 10 | 100.9 (100.9-100.9) | 0.0 | 10 | 100.9 (100.9-100.9) | 0.0 | NA | NA | NA |
|  | 9 | 10 | 101.0 (101.0-101.0) | 0.0 | 10 | 101.0 (101.0-101.0) | 0.0 | NA | NA | NA |
|  | 10 | 8 | 101.2 (101.2-101.2) | 0.0 | 8 | 101.2 (101.2-101.2) | 0.0 | NA | NA | NA |
|  | 11 | 8 | 101.4 (101.4-101.4) | 0.0 | 8 | 101.4 (101.4-101.4) | 0.0 | NA | NA | NA |
|  | 12 | 6 | 79.5 (17.8-98.2) | 19.5 | 6 | 79.5 (17.8-98.2) | 19.5 | NA | NA | NA |
|  | 13 | 4 | 79.7 (17.9-98.4) | 19.5 | 4 | 79.7 (17.9-98.4) | 19.5 | NA | NA | NA |
|  | 14 | 3 | 79.9 (17.9-98.6) | 19.6 | 3 | 79.9 (17.9-98.6) | 19.6 | NA | NA | NA |
|  | 15 | 2 | 80.1 (18.0-98.9) | 19.6 | 2 | 80.1 (18.0-98.9) | 19.6 | NA | NA | NA |
| Other genital | 1 | 11 | 100.0 (100.0-100.0) | 0.0 | 1 | 100.1 (100.1-100.1) | 0.0 | 10 | 100.0 (100.0-100.0) | 0.0 |
|  | 2 | 12 | 100.1 (100.1-100.1) | 0.0 | 2 | 100.2 (100.2-100.2) | 0.0 | 10 | 100.1 (100.1-100.1) | 0.0 |
|  | 3 | 11 | 100.2 (100.2-100.2) | 0.0 | 2 | 100.2 (100.2-100.2) | 0.0 | 9 | 100.1 (100.1-100.1) | 0.0 |
|  | 4 | 11 | 81.2 (43.3-95.1) | 12.1 | 2 | 100.3 (100.3-100.3) | 0.0 | 9 | 76.8 (34.6-93.8) | 14.4 |
|  | 5 | 8 | 81.3 (43.3-95.2) | 12.1 | 2 | 100.4 (100.4-100.4) | 0.0 | 6 | 76.9 (34.6-93.8) | 14.5 |
|  | 6 | 7 | 81.4 (43.3-95.2) | 12.1 | 1 | 100.5 (100.5-100.5) | 0.0 | 6 | 76.9 (34.6-93.9) | 14.5 |
|  | 7 | 5 | 81.4 (43.4-95.3) | 12.1 | 1 | 100.6 (100.6-100.6) | 0.0 | 4 | 77.0 (34.7-94.0) | 14.5 |
|  | 8 | 5 | 81.5 (43.4-95.4) | 12.1 | 1 | 100.7 (100.7-100.7) | 0.0 | 4 | 77.1 (34.7-94.1) | 14.5 |
|  | 9 | 4 | 81.6 (43.4-95.5) | 12.1 | 1 | 100.7 (100.7-100.7) | 0.0 | 3 | 77.1 (34.7-94.2) | 14.5 |
|  | 10 | 4 | 81.7 (43.5-95.6) | 12.1 | 1 | 100.9 (100.9-100.9) | 0.0 | 3 | 77.2 (34.8-94.3) | 14.5 |
|  | 11 | 3 | 81.8 (43.5-95.7) | 12.2 | 1 | 101.0 (101.0-101.0) | 0.0 | 2 | 77.3 (34.8-94.4) | 14.5 |
|  | 12 | 2 | 81.9 (43.6-95.9) | 12.2 | 1 | 101.1 (101.1-101.1) | 0.0 | 1 | 77.5 (34.9-94.6) | 14.6 |
|  | 13 | 1 | 82.1 (43.7-96.1) | 12.2 | NA | NA | NA | 1 | 77.6 (35.0-94.8) | 14.6 |
|  | 14 | 2 | 82.2 (43.8-96.3) | 12.2 | NA | NA | NA | 2 | 77.8 (35.0-95.0) | 14.6 |
|  | 15 | 4 | 82.4 (43.9-96.5) | 12.2 | NA | NA | NA | 4 | 78.0 (35.1-95.2) | 14.7 |
| Carcinoma of urinary tract |  |  |  |  |  |  |  |  |  |  |
| Carcinoma of kidney | 1 | 424 | 94.1 (91.4-96.0) | 1.2 | 245 | 93.4 (89.5-95.9) | 1.6 | 179 | 95.0 (90.6-97.4) | 1.6 |
|  | 2 | 429 | 92.3 (89.2-94.5) | 1.3 | 241 | 91.8 (87.5-94.7) | 1.8 | 188 | 92.9 (88.0-95.8) | 1.9 |
|  | 3 | 412 | 91.1 (87.9-93.5) | 1.4 | 233 | 91.0 (86.6-94.1) | 1.9 | 179 | 91.2 (86.0-94.6) | 2.1 |
|  | 4 | 400 | 89.4 (86.0-92.0) | 1.5 | 227 | 89.8 (85.1-93.1) | 2.0 | 173 | 88.9 (83.2-92.8) | 2.4 |
|  | 5 | 376 | 89.2 (85.8-91.9) | 1.5 | 216 | 89.4 (84.7-92.8) | 2.0 | 160 | 89.0 (83.3-92.8) | 2.4 |
|  | 6 | 372 | 89.0 (85.5-91.7) | 1.6 | 220 | 89.5 (84.8-92.9) | 2.0 | 152 | 88.4 (82.6-92.4) | 2.4 |
|  | 7 | 361 | 88.6 (85.0-91.3) | 1.6 | 219 | 88.7 (83.8-92.2) | 2.1 | 142 | 88.4 (82.6-92.4) | 2.5 |
|  | 8 | 346 | 88.1 (84.5-91.0) | 1.6 | 209 | 88.8 (83.9-92.3) | 2.1 | 137 | 87.1 (81.0-91.4) | 2.6 |
|  | 9 | 347 | 87.1 (83.3-90.1) | 1.7 | 211 | 87.1 (81.9-90.9) | 2.3 | 136 | 87.2 (81.1-91.5) | 2.6 |
|  | 10 | 330 | 86.3 (82.4-89.4) | 1.8 | 200 | 85.8 (80.4-89.8) | 2.4 | 130 | 87.3 (81.2-91.6) | 2.6 |
|  | 11 | 304 | 86.4 (82.6-89.6) | 1.8 | 183 | 85.9 (80.5-90.0) | 2.4 | 121 | 87.4 (81.3-91.7) | 2.6 |
|  | 12 | 289 | 85.6 (81.6-88.9) | 1.8 | 169 | 86.1 (80.7-90.1) | 2.4 | 120 | 85.0 (78.4-89.9) | 2.9 |
|  | 13 | 271 | 85.1 (81.0-88.4) | 1.9 | 165 | 85.7 (80.2-89.9) | 2.4 | 106 | 84.3 (77.4-89.3) | 3.0 |
|  | 14 | 264 | 83.8 (79.5-87.4) | 2.0 | 161 | 84.1 (78.3-88.6) | 2.6 | 103 | 83.5 (76.4-88.7) | 3.1 |
|  | 15 | 250 | 83.6 (79.3-87.2) | 2.0 | 152 | 83.7 (77.8-88.3) | 2.7 | 98 | 83.7 (76.6-88.9) | 3.1 |
| Carcinoma of bladder | 1 | 136 | 76.2 (68.1-82.5) | 3.7 | 77 | 82.8 (72.3-89.7) | 4.3 | 59 | 67.8 (54.3-78.0) | 6.1 |
|  | 2 | 109 | 67.6 (58.9-74.9) | 4.1 | 67 | 74.7 (63.2-83.1) | 5.0 | 42 | 58.4 (44.6-69.9) | 6.5 |
|  | 3 | 94 | 62.7 (53.8-70.4) | 4.2 | 61 | 70.5 (58.7-79.6) | 5.3 | 33 | 52.5 (38.7-64.6) | 6.7 |
|  | 4 | 82 | 59.5 (50.6-67.5) | 4.3 | 56 | 65.2 (53.2-74.9) | 5.6 | 26 | 52.6 (38.8-64.6) | 6.7 |
|  | 5 | 77 | 56.2 (47.1-64.3) | 4.4 | 52 | 62.4 (50.3-72.4) | 5.7 | 25 | 48.2 (34.4-60.7) | 6.8 |
|  | 6 | 73 | 54.5 (45.4-62.7) | 4.4 | 45 | 59.4 (47.1-69.7) | 5.8 | 28 | 48.2 (34.4-60.7) | 6.8 |
|  | 7 | 74 | 53.8 (44.7-62.0) | 4.5 | 43 | 57.9 (45.7-68.4) | 5.9 | 31 | 48.2 (34.4-60.8) | 6.8 |
|  | 8 | 78 | 53.0 (44.0-61.3) | 4.5 | 46 | 58.0 (45.7-68.5) | 5.9 | 32 | 46.7 (33.0-59.2) | 6.8 |
|  | 9 | 79 | 51.6 (42.6-60.0) | 4.5 | 46 | 55.2 (42.9-65.9) | 5.9 | 33 | 46.7 (33.1-59.3) | 6.8 |
|  | 10 | 82 | 51.7 (42.6-60.0) | 4.5 | 48 | 55.3 (43.0-66.0) | 5.9 | 34 | 46.8 (33.1-59.3) | 6.8 |
|  | 11 | 87 | 51.1 (42.1-59.4) | 4.5 | 53 | 54.2 (42.0-65.0) | 5.9 | 34 | 46.8 (33.1-59.4) | 6.8 |
|  | 12 | 82 | 50.5 (41.5-58.9) | 4.5 | 53 | 53.2 (41.1-64.0) | 5.9 | 29 | 46.9 (33.2-59.5) | 6.8 |
|  | 13 | 84 | 50.6 (41.6-59.0) | 4.5 | 53 | 53.3 (41.2-64.2) | 5.9 | 31 | 46.9 (33.2-59.6) | 6.8 |
|  | 14 | 82 | 50.7 (41.6-59.1) | 4.5 | 53 | 53.5 (41.3-64.3) | 5.9 | 29 | 47.0 (33.3-59.7) | 6.8 |
|  | 15 | 87 | 50.8 (41.7-59.2) | 4.5 | 56 | 53.6 (41.3-64.4) | 6.0 | 31 | 47.1 (33.3-59.8) | 6.9 |
| Other urinary | 1 | 13 | 92.4 (57.0-98.9) | 7.3 | 9 | 100.1 (100.1-100.1) | 0.0 | 4 | 75.9 (14.1-96.2) | 20.9 |
|  | 2 | 14 | 72.0 (41.7-88.5) | 11.9 | 10 | 90.1 (47.2-98.7) | 9.5 | 4 | 34.8 (3.6-71.5) | 21.5 |
|  | 3 | 12 | 66.0 (36.7-84.2) | 12.3 | 10 | 80.9 (42.6-95.0) | 12.2 | 2 | 34.8 (3.6-71.5) | 21.5 |
|  | 4 | 12 | 66.0 (36.7-84.3) | 12.3 | 9 | 81.0 (42.7-95.1) | 12.2 | 3 | 34.8 (3.6-71.6) | 21.5 |
|  | 5 | 12 | 66.1 (36.8-84.3) | 12.4 | 9 | 81.1 (42.7-95.2) | 12.3 | 3 | 34.9 (3.6-71.6) | 21.5 |
|  | 6 | 11 | 53.9 (27.1-74.7) | 12.7 | 8 | 70.5 (33.1-89.7) | 14.6 | 3 | 24.0 (2.2-58.9) | 17.3 |
|  | 7 | 9 | 54.0 (27.1-74.8) | 12.8 | 7 | 70.6 (33.1-89.8) | 14.6 | 2 | 24.0 (2.2-59.0) | 17.3 |
|  | 8 | 10 | 54.0 (27.2-74.9) | 12.8 | 8 | 70.7 (33.2-89.9) | 14.6 | 2 | 24.0 (2.2-59.0) | 17.3 |
|  | 9 | 10 | 54.1 (27.2-75.0) | 12.8 | 8 | 70.8 (33.2-90.1) | 14.6 | 2 | 24.1 (2.2-59.0) | 17.3 |
|  | 10 | 9 | 54.2 (27.2-75.1) | 12.8 | 7 | 70.9 (33.3-90.2) | 14.6 | 2 | 24.1 (2.2-59.1) | 17.4 |
|  | 11 | 5 | 54.2 (27.3-75.2) | 12.8 | 3 | 71.0 (33.3-90.3) | 14.7 | 2 | 24.1 (2.2-59.1) | 17.4 |
|  | 12 | 3 | 54.3 (27.3-75.3) | 12.8 | 3 | 71.1 (33.4-90.5) | 14.7 | NA | NA | NA |
|  | 13 | 3 | 54.5 (27.4-75.5) | 12.9 | 3 | 71.3 (33.5-90.7) | 14.7 | NA | NA | NA |
|  | 14 | 4 | 54.6 (27.4-75.6) | 12.9 | 4 | 71.4 (33.5-90.9) | 14.7 | NA | NA | NA |
|  | 15 | 4 | 54.7 (27.5-75.8) | 12.9 | 4 | 71.6 (33.6-91.1) | 14.8 | NA | NA | NA |
| Other invasive carcinomas |  |  |  |  |  |  |  |  |  |  |
|  | 1 | 194 | 52.0 (44.6-58.8) | 3.7 | 90 | 52.4 (41.4-62.2) | 5.4 | 104 | 51.6 (41.4-60.8) | 5.0 |
|  | 2 | 123 | 47.2 (40.0-54.1) | 3.6 | 61 | 45.2 (34.7-55.2) | 5.3 | 62 | 48.9 (38.9-58.2) | 5.0 |
|  | 3 | 107 | 43.7 (36.6-50.6) | 3.6 | 50 | 41.1 (30.9-51.0) | 5.2 | 57 | 46.1 (36.2-55.4) | 4.9 |
|  | 4 | 89 | 41.5 (34.5-48.4) | 3.6 | 41 | 38.8 (28.7-48.7) | 5.2 | 48 | 44.0 (34.2-53.4) | 4.9 |
|  | 5 | 83 | 39.3 (32.4-46.2) | 3.6 | 36 | 38.8 (28.7-48.7) | 5.2 | 47 | 39.7 (30.1-49.1) | 4.9 |
|  | 6 | 84 | 36.7 (29.9-43.5) | 3.5 | 39 | 37.7 (27.7-47.6) | 5.1 | 45 | 35.6 (26.3-45.0) | 4.8 |
|  | 7 | 78 | 35.2 (28.4-42.0) | 3.5 | 33 | 36.6 (26.7-46.5) | 5.1 | 45 | 33.8 (24.8-43.1) | 4.7 |
|  | 8 | 82 | 33.8 (27.2-40.6) | 3.4 | 39 | 34.5 (24.9-44.4) | 5.0 | 43 | 33.0 (24.1-42.3) | 4.7 |
|  | 9 | 82 | 33.9 (27.2-40.6) | 3.4 | 37 | 34.6 (24.9-44.4) | 5.0 | 45 | 33.1 (24.1-42.3) | 4.7 |
|  | 10 | 85 | 33.5 (26.8-40.2) | 3.4 | 39 | 33.6 (24.1-43.4) | 5.0 | 46 | 33.1 (24.1-42.3) | 4.7 |
|  | 11 | 86 | 33.1 (26.5-39.8) | 3.4 | 38 | 32.7 (23.3-42.5) | 4.9 | 48 | 33.1 (24.1-42.4) | 4.7 |
|  | 12 | 90 | 31.9 (25.4-38.5) | 3.4 | 40 | 31.8 (22.5-41.5) | 4.9 | 50 | 31.7 (23.0-40.9) | 4.6 |
|  | 13 | 85 | 31.5 (25.1-38.1) | 3.3 | 34 | 31.8 (22.5-41.6) | 4.9 | 51 | 31.1 (22.4-40.2) | 4.6 |
|  | 14 | 85 | 30.4 (24.1-36.9) | 3.3 | 36 | 31.9 (22.6-41.6) | 4.9 | 49 | 29.1 (20.8-38.0) | 4.4 |
|  | 15 | 87 | 29.6 (23.4-36.1) | 3.3 | 38 | 30.1 (21.0-39.7) | 4.8 | 49 | 29.2 (20.8-38.0) | 4.4 |
| Neuroendocrine Tumors (NET) |  |  |  |  |  |  |  |  |  |  |
|  | 1 | 780 | 98.6 (97.5-99.3) | 0.4 | 312 | 97.5 (95.9-98.8) | 0.9 | 468 | 99.4 (98.0-99.8) | 0.4 |
|  | 2 | 808 | 97.9 (96.5-98.7) | 0.5 | 319 | 96.2 (93.4-97.9) | 1.1 | 489 | 99.0 (97.5-99.6) | 0.5 |
|  | 3 | 749 | 97.3 (95.9-98.3) | 0.6 | 300 | 95.9 (93.9-97.7) | 1.1 | 449 | 98.3 (96.5-99.2) | 0.6 |
|  | 4 | 696 | 96.8 (95.2-97.9) | 0.7 | 277 | 96.0 (93.1-97.8) | 1.1 | 419 | 97.3 (95.2-98.5) | 0.8 |
|  | 5 | 657 | 96.7 (95.1-97.8) | 0.7 | 261 | 96.1 (93.1-97.8) | 1.2 | 396 | 97.1 (94.9-98.4) | 0.8 |
|  | 6 | 610 | 96.7 (95.1-97.9) | 0.7 | 233 | 96.1 (93.2-97.9) | 1.2 | 377 | 97.1 (94.9-98.4) | 0.8 |
|  | 7 | 565 | 96.8 (95.2-97.9) | 0.7 | 210 | 96.2 (93.3-98.0) | 1.2 | 355 | 97.2 (95.0-98.5) | 0.8 |
|  | 8 | 508 | 96.7 (95.9-97.8) | 0.7 | 186 | 95.7 (92.5-97.7) | 1.3 | 322 | 97.2 (95.1-98.5) | 0.8 |
|  | 9 | 462 | 96.3 (94.4-97.6) | 0.8 | 172 | 95.2 (91.6-97.4) | 1.4 | 290 | 96.9 (94.6-98.3) | 0.9 |
|  | 10 | 403 | 96.3 (94.5-97.6) | 0.8 | 150 | 95.3 (91.7-97.5) | 1.4 | 253 | 97.0 (94.6-98.4) | 0.9 |
|  | 11 | 356 | 96.4 (94.6-97.7) | 0.8 | 133 | 95.5 (91.8-97.6) | 1.4 | 223 | 97.1 (94.7-98.5) | 0.9 |
|  | 12 | 313 | 95.9 (93.7-97.4) | 0.9 | 116 | 93.8 (89.0-96.7) | 1.9 | 197 | 97.2 (94.8-98.6) | 0.9 |
|  | 13 | 311 | 96.9 (93.8-97.5) | 0.9 | 117 | 93.9 (89.1-96.8) | 1.9 | 194 | 97.3 (94.9-98.7) | 0.9 |
|  | 14 | 312 | 96.1 (93.9-97.7) | 0.9 | 115 | 94.1 (89.3-97.0) | 1.9 | 197 | 97.4 (95.0-98.8) | 0.9 |
|  | 15 | 318 | 95.2 (92.7-97.1) | 1.1 | 114 | 92.5 (86.9-96.0) | 2.3 | 204 | 97.0 (94.2-98.6) | 1.1 |
| Neuroendocrine carcinomas (NEC) |  |  |  |  |  |  |  |  |  |  |
|  | 1 | 27 | 62.7 (43.0-77.2) | 8.8 | 15 | 46.3 (22.8-67.0) | 11.9 | 12 | 84.5 (50.8-95.9) | 10.1 |
|  | 2 | 17 | 44.1 (25.8-61.1) | 9.3 | 7 | 39.8 (17.6-61.4) | 11.9 | 10 | 50.4 (21.3-73.8) | 14.4 |
|  | 3 | 12 | 44.2 (25.8-61.1) | 9.3 | 5 | 39.8 (17.6-61.4) | 11.9 | 7 | 50.4 (21.3-73.9) | 14.4 |
|  | 4 | 11 | 39.7 (21.8-57.2) | 9.4 | 6 | 31.7 (11.3-54.7) | 11.9 | 5 | 50.4 (21.3-73.9) | 14.4 |
|  | 5 | 10 | 35.9 (18.7-53.5) | 9.2 | 5 | 31.8 (11.3-54.7) | 11.9 | 5 | 40.9 (14.4-66.3) | 14.5 |
|  | 6 | 8 | 35.9 (18.7-53.5) | 9.3 | 5 | 31.8 (11.3-54.8) | 11.9 | 3 | 41.0 (14.4-66.4) | 14.5 |
|  | 7 | 8 | 31.0 (14.6-49.1) | 9.2 | 4 | 23.2 (5.9-47.0) | 11.4 | 4 | 41.0 (14.4-66.4) | 14.5 |
|  | 8 | 6 | 25.5 (10.3-44.1) | 9.1 | 3 | 15.9 (2.9-38.7) | 9.9 | 3 | 41.0 (14.4-66.5) | 14.5 |
|  | 9 | 5 | 25.6 (10.3-44.1) | 9.1 | 2 | 15.9 (2.9-38.8) | 9.9 | 3 | 41.1 (14.4-66.5) | 14.5 |
|  | 10 | 5 | 25.6 (10.3-44.2) | 9.1 | 2 | 15.9 (2.9-38.8) | 9.9 | 3 | 41.1 (14.4-66.6) | 14.5 |
|  | 11 | 4 | 25.6 (10.3-44.3) | 9.1 | 1 | 16.0 (2.9-38.9) | 9.9 | 3 | 41.2 (14.5-66.7) | 14.6 |
|  | 12 | 5 | 25.7 (10.4-44.3) | 9.1 | 1 | 16.0 (2.9-39.0) | 9.9 | 4 | 41.2 (14.5-66.8) | 14.6 |
|  | 13 | 6 | 25.7 (10.4-44.4) | 9.1 | 2 | 16.1 (2.9-39.1) | 9.9 | 4 | 41.3 (14.5-66.9) | 14.6 |
|  | 14 | 5 | 25.8 (10.4-44.5) | 9.2 | 2 | 16.1 (2.9-39.2) | 10.0 | 3 | 41.3 (14.5-67.0) | 14.6 |
|  | 15 | 6 | 25.8 (10.4-44.6) | 9.2 | 2 | 16.2 (2.9-39.3) | 10.0 | 4 | 41.4 (14.5-67.1) | 14.7 |
| Miscellaneous specified neoplasms |  |  |  |  |  |  |  |  |  |  |
|  | 1 | 116 | 86.9 (79.2-91.9) | 3.2 | 49 | 83.3 (69.3-91.3) | 5.4 | 67 | 89.5 (79.1-94.8) | 3.8 |
|  | 2 | 105 | 79.8 (70.9-86.3) | 3.9 | 43 | 69.5 (53.9-80.7) | 6.8 | 62 | 87.7 (76.8-93.7) | 4.1 |
|  | 3 | 70 | 72.9 (62.6-80.8) | 4.6 | 28 | 62.7 (45.7-75.7) | 7.7 | 42 | 80.6 (67.1-89.0) | 5.5 |
|  | 4 | 50 | 66.7 (55.5-75.7) | 5.2 | 18 | 48.5 (31.0-64.0) | 8.6 | 32 | 80.7 (67.2-89.1) | 5.5 |
|  | 5 | 43 | 65.1 (53.7-74.4) | 5.3 | 14 | 48.5 (31.0-64.0) | 8.6 | 29 | 77.9 (63.5-87.2) | 5.9 |
|  | 6 | 41 | 63.4 (51.8-73.0) | 5.4 | 12 | 44.1 (26.5-60.4) | 8.9 | 29 | 77.9 (63.5-87.2) | 6.0 |
|  | 7 | 39 | 63.5 (51.8-73.1) | 5.4 | 10 | 44.1 (26.5-60.5) | 8.9 | 29 | 78.0 (63.6-87.3) | 6.0 |
|  | 8 | 42 | 63.5 (51.9-73.1) | 5.4 | 12 | 44.2 (26.5-60.5) | 8.9 | 30 | 78.0 (63.6-87.3) | 6.0 |
|  | 9 | 43 | 63.6 (51.9-73.2) | 5.4 | 12 | 44.2 (26.5-60.6) | 9.0 | 31 | 78.1 (63.6-87.4) | 6.0 |
|  | 10 | 40 | 61.9 (50.0-71.8) | 5.6 | 11 | 40.2 (22.9-57.0) | 9.0 | 29 | 78.1 (63.7-87.5) | 6.0 |
|  | 11 | 39 | 61.9 (50.1-71.8) | 5.6 | 12 | 40.2 (22.9-57.1) | 9.0 | 27 | 78.2 (63.7-87.5) | 6.0 |
|  | 12 | 35 | 62.0 (50.1-71.9) | 5.6 | 13 | 40.3 (22.9-57.2) | 9.0 | 22 | 78.3 (63.8-87.6) | 6.0 |
|  | 13 | 35 | 62.1 (50.2-72.0) | 5.6 | 14 | 40.3 (23.0-57.2) | 9.1 | 21 | 78.4 (63.9-87.7) | 6.0 |
|  | 14 | 37 | 60.2 (48.1-70.5) | 5.8 | 15 | 40.4 (23.0-57.3) | 9.1 | 22 | 74.4 (58.0-85.4) | 6.9 |
|  | 15 | 36 | 58.6 (46.3-69.1) | 5.8 | 16 | 40.5 (23.0-57.4) | 9.1 | 20 | 70.8 (53.4-82.9) | 7.5 |
| Unspecified malignant neoplasms except CNS |  |  |  |  |  |  |  |  |  |  |
|  | 1 | 60 | 50.7 (36.9-63.0) | 6.8 | 43 | 53.3 (36.8-67.3) | 7.9 | 17 | 43.9 (19.2-66.2) | 12.8 |
|  | 2 | 36 | 47.5 (34.0-59.9) | 6.7 | 28 | 49.1 (33.1-63.2) | 7.8 | 8 | 43.9 (19.2-66.3) | 12.8 |
|  | 3 | 32 | 45.8 (32.4-58.2) | 6.7 | 26 | 46.9 (31.3-61.1) | 7.8 | 6 | 43.9 (19.3-66.3) | 12.8 |
|  | 4 | 29 | 44.1 (30.9-56.5) | 6.7 | 23 | 44.7 (29.3-59.0) | 7.7 | 6 | 43.9 (19.3-66.4) | 12.8 |
|  | 5 | 22 | 44.1 (30.9-56.6) | 6.7 | 17 | 44.7 (29.3-59.0) | 7.7 | 5 | 44.0 (19.3-66.4) | 12.8 |
|  | 6 | 21 | 42.0 (28.8-54.6) | 6.7 | 18 | 44.7 (29.3-59.0) | 7.8 | 3 | 30.1 (7.3-57.8) | 14.4 |
|  | 7 | 20 | 42.0 (28.8-54.6) | 6.7 | 17 | 44.8 (29.4-59.1) | 7.8 | 3 | 30.2 (7.3-57.8) | 14.4 |
|  | 8 | 22 | 39.9 (26.9-52.6) | 6.7 | 19 | 42.2 (27.0-56.6) | 7.7 | 3 | 30.2 (7.3-57.9) | 14.4 |
|  | 9 | 21 | 39.9 (26.9-52.7) | 6.7 | 18 | 42.2 (27.0-56.7) | 7.8 | 3 | 30.2 (7.3-58.0) | 14.5 |
|  | 10 | 22 | 40.0 (27.0-52.7) | 6.7 | 18 | 42.3 (27.0-56.7) | 7.8 | 4 | 30.2 (7.3-58.0) | 14.5 |
|  | 11 | 22 | 40.0 (27.0-52.8) | 6.7 | 18 | 42.3 (27.1-56.8) | 7.8 | 4 | 30.3 (7.3-58.1) | 14.5 |
|  | 12 | 21 | 40.1 (27.0-52.9) | 6.7 | 15 | 42.4 (27.1-56.9) | 7.8 | 6 | 30.3 (7.3-58.2) | 14.5 |
|  | 13 | 21 | 40.1 (27.1-52.9) | 6.7 | 14 | 42.4 (27.1-57.0) | 7.8 | 7 | 30.4 (7.3-58.2) | 14.5 |
|  | 14 | 21 | 40.2 (27.1-53.0) | 6.7 | 15 | 42.5 (27.2-57.0) | 7.8 | 6 | 30.4 (7.4-58.3) | 14.5 |
|  | 15 | 22 | 40.2 (27.1-53.1) | 6.7 | 16 | 42.5 (27.2-57.1) | 7.8 | 6 | 30.4 (7.4-58.4) | 14.6 |

Abbreviation: RS=Relative Survival, CI=Confidence Intervals, SE=Standard Error, CNS=Central Nervous System, NA=Not Applicable.

* Relative survival estimates were obtained by using the hybrid approach developed by Brenner and Ratchet (2004). Survival follow-up was truncated at 31 December 2022. The selected period window was 2012-2022. Start period window was defined as the date of diagnosis for AYAs diagnosed between 1 January 2012 and 31 December 2021. For AYAs diagnosed before 2012, the start of the time at risk was 1 January 2013.

** Cancer types were categorized into diagnostic groups according to the revised morphology based AYA-specific classification scheme developed by Barr et al. (2021).

*** The Netherlands Cancer Registry does not collect data about basal-cell skin and lip carcinomas.

|  |  | **Years of follow-up** | | | | | | | | | | |  |  |
| --- | --- | --- | --- | --- | --- | --- | --- | --- | --- | --- | --- | --- | --- | --- |
|  |  | 2012 | 2013 | 2014 | 2015 | 2016 | 2017 | 2018 | 2019 | 2020 | 2021 | 2022 |  |  |
| **Years of diagnosis** | 1989 | 23 | 24 | 25 | 26 | 27 | 28 | 29 | 30 | 31 | 32 | 33 | 1989 | **Years of diagnosis** |
|  | 1990 | 22 | 23 | 24 | 25 | 26 | 27 | 28 | 29 | 30 | 31 | 32 | 1990 |  |
|  | 1991 | 21 | 22 | 23 | 24 | 25 | 26 | 27 | 28 | 29 | 30 | 31 | 1991 |  |
|  | 1992 | 20 | 21 | 22 | 23 | 24 | 25 | 26 | 27 | 28 | 29 | 30 | 1992 |  |
|  | 1993 | 19 | 20 | 21 | 22 | 23 | 24 | 25 | 26 | 27 | 28 | 29 | 1993 |  |
|  | 1994 | 18 | 19 | 20 | 21 | 22 | 23 | 24 | 25 | 26 | 27 | 28 | 1994 |  |
|  | 1995 | 17 | 18 | 19 | 20 | 21 | 22 | 23 | 24 | 25 | 26 | 27 | 1995 |  |
|  | 1996 | 16 | 17 | 18 | 19 | 20 | 21 | 22 | 23 | 24 | 25 | 26 | 1996 |  |
|  | 1997 | 15 | 16 | 17 | 18 | 19 | 20 | 21 | 22 | 23 | 24 | 25 | 1997 |  |
|  | 1998 | 14 | 15 | 16 | 17 | 18 | 19 | 20 | 21 | 22 | 23 | 24 | 1998 |  |
|  | 1999 | 13 | 14 | 15 | 16 | 17 | 18 | 19 | 20 | 21 | 22 | 23 | 1999 |  |
|  | 2000 | 12 | 13 | 14 | 15 | 16 | 17 | 18 | 19 | 20 | 21 | 22 | 2000 |  |
|  | 2001 | 11 | 12 | 13 | 14 | 15 | 16 | 17 | 18 | 19 | 20 | 21 | 2001 |  |
|  | 2002 | 10 | 11 | 12 | 13 | 14 | 15 | 16 | 17 | 18 | 19 | 20 | 2002 |  |
|  | 2003 | 9 | 10 | 11 | 12 | 13 | 14 | 15 | 16 | 17 | 18 | 19 | 2003 |  |
|  | 2004 | 8 | 9 | 10 | 11 | 12 | 13 | 14 | 15 | 16 | 17 | 18 | 2004 |  |
|  | 2005 | 7 | 8 | 9 | 10 | 11 | 12 | 13 | 14 | 15 | 16 | 17 | 2005 |  |
|  | 2006 | 6 | 7 | 8 | 9 | 10 | 11 | 12 | 13 | 14 | 15 | 16 | 2006 |  |
|  | 2007 | 5 | 6 | 7 | 8 | 9 | 10 | 11 | 12 | 13 | 14 | 15 | 2007 |  |
|  | 2008 | 4 | 5 | 6 | 7 | 8 | 9 | 10 | 11 | 12 | 13 | 14 | 2008 |  |
|  | 2009 | 3 | 4 | 5 | 6 | 7 | 8 | 9 | 10 | 11 | 12 | 13 | 2009 |  |
|  | 2010 | 2 | 3 | 4 | 5 | 6 | 7 | 8 | 9 | 10 | 11 | 12 | 2010 |  |
|  | 2011 | 1 | 2 | 3 | 4 | 5 | 6 | 7 | 8 | 9 | 10 | 11 | 2011 |  |
|  | 2012 | 0 | 1 | 2 | 3 | 4 | 5 | 6 | 7 | 8 | 9 | 10 | 2012 |  |
|  | 2013 |  | 0 | 1 | 2 | 3 | 4 | 5 | 6 | 7 | 8 | 9 | 2013 |  |
|  | 2014 |  |  | 0 | 1 | 2 | 3 | 4 | 5 | 6 | 7 | 8 | 2014 |  |
|  | 2015 |  |  |  | 0 | 1 | 2 | 3 | 4 | 5 | 6 | 7 | 2015 |  |
|  | 2016 |  |  |  |  | 0 | 1 | 2 | 3 | 4 | 5 | 6 | 2016 |  |
|  | 2017 |  |  |  |  |  | 0 | 1 | 2 | 3 | 4 | 5 | 2017 |  |
|  | 2018 |  |  |  |  |  |  | 0 | 1 | 2 | 3 | 4 | 2018 |  |
|  | 2019 |  |  |  |  |  |  |  | 0 | 1 | 2 | 3 | 2019 |  |
|  | 2020 |  |  |  |  |  |  |  |  | 0 | 1 | 2 | 2020 |  |
|  | 2021 |  |  |  |  |  |  |  |  |  | 0 | 1 | 2021 |  |

# Supplementary Figure 1. Structure of the follow-up data using the hybrid approach. The numbers within the cells represent the minimum years of follow-up (column) since diagnosis (row). For the calculation of five-year relative survival at diagnosis, as well as each additional year survived up to 10 years post-diagnosis (i.e., conditional five-year relative survival), the data in the grey-shaded areas were used.

# Supplementary Table 3. Five-year conditional relative survival with 95% confidence intervals up to 10 years post-diagnosis of male adolescents and young adults (AYAs, aged 18-39 years) diagnosed with an invasive solid malignancy in the Netherlands between 1998-2021. CRS estimates were only generated for cancer types with a number at risk of more than n=100 at the time of diagnosis.

|  | **CRS (95% CI)** | | | | | | | | | | | **CRS PE > 95%  from year** |
| --- | --- | --- | --- | --- | --- | --- | --- | --- | --- | --- | --- | --- |
| **Survival years** | **0*** | **1** | **2** | **3** | **4** | **5** | **6** | **7** | **8** | **9** | **10** |  |
| **Total** | 86.8 (86.2-87.4) | 91.2 (90.7-91.8) | 93.9 (93.4-94.4) | 95.2 (94.7-95.6) | 96.2 (95.8-96.6) | 96.7 (96.3-97.0) | 96.9 (96.6-97.3) | 97.0 (96.7-97.4) | 97.4 (97.0-97.7) | 97.6 (97.3-98.0) | 97.8 (97.4-98.2) | 3 |
| **Age at diagnosis (Years)** |  |  |  |  |  |  |  |  |  |  |  |  |
| 18-20 | 89.0 (86.1-91.3) | 91.6 (89.3-93.9) | 94.3 (92.4-96.3) | 95.9 (94.2-97.6) | 96.7 (95.2-98.3) | 96.9 (95.3-98.4) | 97.2 (95.7-98.7) | 98.0 (96.7-99.3) | 98.2 (96.9-99.4) | 98.9 (97.9-100.0) | 99.1 (98.1-100.1) | 3 |
| 21-24 | 90.5 (88.8-92.0) | 93.3 (91.9-94.7) | 95.8 (94.6-97.0) | 97.0 (96.0-98.1) | 98.0 (97.1-98.9) | 97.9 (97.0-98.9) | 98.0 (97.1-99.0) | 97.6 (96.6-98.7) | 98.0 (97.1-99.0) | 97.7 (96.7-98.8) | 98.1 (97.1-99.1) | 2 |
| 25-29 | 90.2 (89.0-91.3) | 93.1 (92.0-94.1) | 94.8 (93.9-95.7) | 96.0 (95.2-96.9) | 96.8 (96.0-97.6) | 97.1 (96.3-97.9) | 97.4 (96.6-98.2) | 97.8 (97.1-98.5) | 97.9 (97.1-98.6) | 98.3 (97.7-99.0) | 98.4 (97.7-99.1) | 3 |
| 30-34 | 87.6 (86.4-88.7) | 92.2 (91.2-93.2) | 94.5 (93.6-95.4) | 95.6 (94.8-96.4) | 96.7 (96.0-97.4) | 97.2 (96.5-97.9) | 96.8 (96.1-97.6) | 96.7 (96.0-97.5) | 97.1 (96.4-97.9) | 97.5 (96.8-98.2) | 97.4 (96.7-98.2) | 3 |
| 35-39 | 82.5 (81.3-83.7) | 88.5 (87.4-89.5) | 92.1 (91.2-93.0) | 93.6 (92.7-94.5) | 94.6 (93.8-95.4) | 95.5 (94.7-96.3) | 96.4 (95.7-97.1) | 96.5 (95.8-97.3) | 97.0 (96.3-97.7) | 97.2 (96.5-97.9) | 97.5 (96.8-98.2) | 5 |
| **Diagnostic group**** |  |  |  |  |  |  |  |  |  |  |  |  |
| **CNS and other intracranial and intraspinal neoplasms** | 63.3 (59.9-66.6) | 66.8 (63.3-70.3) | 72.3 (68.7-75.8) | 73.0 (69.3-76.7) | 73.7 (69.9-77.5) | 73.7 (69.7-77.7) | 72.3 (68.0-76.5) | 71.2 (66.7-75.7) | 73.4 (68.7-78.0) | 75.3 (70.5-80.1) | 78.5 (73.7-83.3) | NA*** |
| **Sarcomas** |  |  |  |  |  |  |  |  |  |  |  |  |
| Soft tissue sarcomas | 72.7 (67.1-77.5) | 80.8 (76.0-85.6) | 87.6 (83.4-91.7) | 91.0 (87.4-94.7) | 93.5 (90.3-96.7) | 96.4 (93.9-98.9) | 96.1 (93.6-98.7) | 97.0 (94.7-99.3) | 97.1 (94.9-99.4) | 98.4 (96.6-100.2) | 98.2 (96.4-100.1) | 5 |
| Bone sarcomas | 80.1 (74.6-84.5) | 82.0 (77.2-86.8) | 87.5 (83.3-91.7) | 91.9 (88.3-95.4) | 95.2 (92.4-98.1) | 95.6 (92.9-98.4) | 96.8 (94.3-99.3) | 95.6 (92.6-98.6) | 96.2 (93.2-99.1) | 95.4 (92.0-98.7) | 95.9 (92.6-99.1) | 4 |
| Other sarcomas | 66.0 (51.9-76.9) | 85.6 (74.7-96.4) | 90.1 (80.7-99.6) | 92.6 (84.2-101.0) | 97.7 (92.5-102.9) | 95.6 (89.1-102.1) | 95.6 (89.2-102.1) | 95.7 (89.2-102.2) | 95.7 (89.2-102.2) | 95.8 (89.3-102.3) | 97.2 (90.7-103.8) | 4 |
| **Blood and lymphatic vessel tumors** | 81.6 (72.6-88.0) | 94.1 (89.2-98.9) | 97.3 (93.8-100.7) | 98.3 (95.4-101.2) | 98.4 (95.5-101.2) | 99.4 (97.4-101.5) | 99.7 (97.9-101.4) | 99.7 (98.0-101.5) | 98.9 (96.4-101.4) | 97.2 (93.7-100.6) | 97.3 (93.8-100.7) | 2 |
| **Gonadal and related tumors** |  |  |  |  |  |  |  |  |  |  |  |  |
| Testis, Germ cell and trophoblastic | 98.8 (98.4-99.1) | 99.2 (98.9-99.5) | 99.6 (99.3-99.8) | 99.9 (99.7-100.1) | 100.0 (99.8-100.2) | 99.9 (99.7-100.1) | 99.8 (99.5-100.0) | 99.9 (99.7-100.1) | 99.9 (99.7-100.2) | 99.9 (99.6-100.1) | 99.8 (99.5-100.1) | 0 |
| (Other) Germ cell, non-germ cell and trophoblastic tumors | 87.3 (79.7-92.3) | 94.3 (89.6-99.0) | 97.9 (94.6-101.2) | 97.9 (94.7-101.2) | 97.9 (94.5-101.3) | 99.2 (96.8-101.5) | 99.2 (96.9-101.5) | 98.0 (94.7-101.3) | 98.0 (94.7-101.4) | 99.3 (96.8-101.7) | 98.1 (94.8-101.4) | 2 |
| **Melanoma, malignant** | 93.4 (92.2-94.4) | 94.0 (92.9-95.1) | 94.9 (93.9-96.0) | 95.6 (94.6-96.5) | 96.4 (95.6-97.3) | 97.2 (96.4-98.0) | 97.5 (96.7-98.3) | 97.9 (97.2-98.7) | 98.2 (97.5-98.9) | 98.5 (97.8-99.2) | 98.8 (98.1-99.4) | 3 |
| **Carcinomas** |  |  |  |  |  |  |  |  |  |  |  |  |
| Thyroid carcinoma | 98.2 (96.0-99.3) | 98.8 (97.4-100.1) | 99.4 (98.3-100.5) | 98.7 (97.3-100.2) | 99.4 (98.3-100.6) | 99.4 (98.3-100.6) | 99.5 (98.3-100.7) | 99.5 (98.4-100.7) | 99.9 (98.8-100.9) | 99.5 (98.2-100.9) | 99.5 (98.1-100.9) | 0 |
| Carcinoma of head and neck | 85.0 (80.4-88.7) | 90.4 (86.8-93.9) | 93.5 (90.5-96.5) | 95.3 (92.6-97.9) | 96.4 (94.1-98.8) | 95.8 (93.3-98.3) | 95.9 (93.3-98.4) | 94.9 (92.1-97.6) | 95.3 (92.6-98.0) | 95.7 (93.1-98.3) | 94.3 (91.3-97.2) | 3 |
| Carcinoma of gastrointestinal tract |  |  |  |  |  |  |  |  |  |  |  |  |
| Carcinoma of stomach | 36.5 (28.5-44.5) | 57.8 (46.9-68.7) | 74.7 (63.5-85.9) | 79.9 (69.1-90.8) | 81.3 (70.5-92.0) | 86.3 (76.5-96.1) | 89.9 (80.9-98.8) | 88.9 (79.2-98.7) | 91.0 (81.8-100.2) | 90.6 (81.0-100.2) | 88.1 (77.6-98.7) | NA*** |
| Carcinoma of colon | 66.5 (61.3-71.1) | 76.7 (72.0-81.5) | 85.7 (81.4-89.9) | 90.5 (86.8-94.1) | 94.1 (91.0-97.1) | 96.8 (94.4-99.2) | 97.7 (95.5-99.8) | 97.3 (95.0-99.6) | 97.8 (95.7-100.0) | 97.4 (95.0-99.8) | 97.9 (95.7-100.1) | 5 |
| Carcinoma of rectum | 67.5 (61.2-73.0) | 67.9 (61.8-74.0) | 71.5 (65.3-77.7) | 75.1 (68.9-81.2) | 79.5 (73.6-85.5) | 84.0 (78.4-89.6) | 89.9 (85.1-94.8) | 92.9 (88.6-97.2) | 90.4 (85.4-95.4) | 92.8 (88.3-97.4) | 94.8 (90.7-98.9) | NA*** |
| Carcinoma of liver and intrahepatic bile ducts (IBD) | 39.2 (26.5-51.7) | 70.9 (53.6-88.3) | 78.8 (61.9-95.6) | 81.9 (65.4-98.3) | 94.9 (84.4-105.4) | 95.0 (84.4-105.5) | 100.5 (100.5-100.5) | 100.6 (100.6-100.6) | 84.5 (55.6-113.5) | 84.6 (55.6-113.6) | 84.7 (55.7-113.7) | 6**** |
| Carcinoma of lung, bronchus, and trachea | 24.3 (18.8-30.2) | 38.4 (29.6-47.2) | 52.9 (42.0-63.8) | 63.4 (51.8-74.9) | 71.5 (59.5-83.5) | 79.8 (68.3-91.3) | 85.9 (75.0-96.8) | 81.7 (68.9-94.5) | 81.7 (68.9-94.6) | 86.3 (74.4-98.2) | 85.1 (72.3-98.0) | NA*** |
| Carcinoma of skin (if collected)***** | 96.8 (93.0-98.6) | 97.8 (95.5-100.0) | 98.8 (97.0-100.6) | 98.8 (96.8-100.7) | 99.9 (98.6-101.1) | 98.7 (96.5-100.8) | 98.7 (96.6-100.9) | 97.5 (94.7-100.3) | 96.8 (93.7-100.0) | 94.7 (90.8-98.6) | 93.7 (89.3-98.1) | 0 |
| Carcinoma of breast | 100.4 (100.4-100.4) | 100.4 (100.4-100.4) | 88.4 (66.2-110.6) | 88.4 (66.2-110.6) | 77.2 (48.9-105.5) | 77.3 (48.9-105.6) | 77.3 (49.0-105.6) | 87.9 (64.5-111.4) | 88.0 (64.5-111.5) | 100.9 (-)****** | 101 (-)****** | 0**** |
| Carcinoma of urinary tract |  |  |  |  |  |  |  |  |  |  |  |  |
| Carcinoma of kidney | 89.4 (84.7-92.8) | 95.8 (93.0-98.6) | 96.6 (93.9-99.2) | 97.5 (95.2-99.9) | 97.0 (94.4-99.6) | 95.9 (92.9-98.9) | 96.0 (93.0-99.0) | 97.1 (94.4-99.8) | 96.5 (93.6-99.5) | 96.6 (93.4-99.8) | 97.6 (94.6-100.6) | 1 |
| Carcinoma of bladder | 62.4 (50.3-72.4) | 71.7 (60.1-83.3) | 77.6 (66.2-89.0) | 82.2 (71.4-93.1) | 84.7 (73.9-95.5) | 88.6 (78.7-98.5) | 91.3 (82.6-100.1) | 91.9 (83.5-100.2) | 91.9 (83.6-100.3) | 96.8 (91.3-102.3) | 96.9 (91.4-102.4) | 9 |
| Other invasive carcinomas | 38.8 (28.7-48.7) | 72.0 (59.3-84.6) | 80.8 (68.6-93.0) | 84.1 (72.1-96.0) | 89.2 (78.7-99.6) | 86.7 (75.4-98.0) | 86.8 (75.6-98.0) | 87.0 (75.9-98.1) | 92.2 (83.1-101.4) | 92.3 (83.1-101.5) | 89.5 (79.1-100.0) | NA*** |
| Neuroendocrine Tumors (NET) | 96.1 (93.1-97.8) | 98.6 (97.1-100.1) | 100.0 (99.3-100.7) | 99.8 (98.6-101.0) | 99.2 (97.4-100.9) | 99.2 (97.5-101.0) | 99.3 (97.5-101.0) | 97.4 (94.3-100.6) | 98.1 (95.2-101.0) | 98.8 (96.2-101.4) | 97.0 (93.3-100.7) | 0 |
| Miscellaneous specified neoplasms | 48.5 (31.0-64.0) | 52.9 (33.0-72.8) | 63.5 (41.5-85.5) | 70.5 (48.3-92.7) | 91.2 (74.0-108.5) | 82.8 (60.7-105.0) | 91.3 (74.0-108.5) | 91.3 (74.0-108.6) | 91.4 (74.1-108.6) | 91.4 (74.1-108.7) | 100.7 (-)****** | 10 |

Abbreviation: CRS=Conditional Relative Survival, CI=Confidence Intervals, PE=Point-estimate, CNS=Central Nervous System, NA=Not Applicable.

* Estimates for the CRS for 0 years survived correspond with the five-year relative survival at the time of diagnosis.

** Cancer types were categorized into diagnostic groups according to the revised morphology based AYA-specific classification scheme developed by Barr et al. (2021). Cancer types were color coded into three groups based on the five-year CRS estimates: CRS>95% from diagnosis or within five years post-diagnosis (green), CRS>95% between five and ten years post-diagnosis (orange), and CRS<95% in ten years post-diagnosis (red).

*** NA was indicated whenever the CRS point-estimate did not surpass the 95% minimal excess mortality threshold in ten years post-diagnosis.

**** Passes the threshold in just one or a few non-consecutive years.

***** The Netherlands Cancer Registry does not collect data about basal-cell skin and lip carcinomas.

*******95% CI could not be calculated due to standard error calculation resulting in negative root numbers.

# Supplementary Table 4. Five-year conditional relative survival with 95% confidence intervals up to 10 years post-diagnosis of female adolescents and young adults (AYAs, aged 18-39 years) diagnosed with an invasive solid malignancy in the Netherlands between 1998-2021. CRS estimates were only generated for cancer types with a number at risk of more than n=100 at the time of diagnosis.

|  | **CRS (95% CI)** | | | | | | | | | | | **CRS PE > 95%  from year** |
| --- | --- | --- | --- | --- | --- | --- | --- | --- | --- | --- | --- | --- |
| **Survival years** | **0*** | **1** | **2** | **3** | **4** | **5** | **6** | **7** | **8** | **9** | **10** |  |
| **Total** | 88.5 (88.0-88.9) | 90.5 (90.1-90.9) | 92.5 (92.1-92.9) | 93.6 (93.3-94.0) | 94.6 (94.2-95.0) | 95.3 (94.9-95.6) | 95.8 (95.5-96.2) | 96.1 (95.8-96.5) | 96.4 (96.1-96.8) | 96.6 (96.3-96.9) | 96.7 (96.4-97.1) | 5 |
| **Age at diagnosis (Years)** |  |  |  |  |  |  |  |  |  |  |  |  |
| 18-20 | 90.0 (86.8-92.4) | 93.6 (91.3-95.9) | 94.8 (92.7-97.0) | 95.7 (93.8-97.7) | 96.2 (94.3-98.1) | 98.0 (96.6-99.4) | 97.9 (96.4-99.4) | 98.4 (97.0-99.8) | 97.7 (96.0-99.4) | 98.0 (96.4-99.6) | 98.3 (96.8-99.8) | 3 |
| 21-24 | 91.1 (89.1-92.7) | 92.9 (91.2-94.5) | 94.6 (93.1-96.0) | 95.5 (94.2-96.9) | 96.4 (95.2-97.7) | 96.7 (95.5-97.9) | 96.4 (95.1-97.7) | 97.1 (95.9-98.3) | 97.5 (96.3-98.6) | 97.7 (96.6-98.8) | 98.7 (97.8-99.6) | 3 |
| 25-29 | 89.1 (87.8-90.2) | 91.3 (90.3-92.4) | 93.6 (92.6-94.6) | 94.9 (94.0-95.8) | 95.5 (94.6-96.3) | 96.1 (95.3-97.0) | 97.0 (96.3-97.8) | 97.1 (96.3-97.9) | 97.1 (96.3-97.9) | 97.4 (96.6-98.2) | 97.6 (96.8-98.4) | 4 |
| 30-34 | 89.3 (88.4-90.1) | 90.8 (90.0-91.6) | 92.7 (92.0-93.5) | 94.0 (93.3-94.7) | 94.9 (94.3-95.6) | 95.5 (94.8-96.1) | 96.1 (95.5-96.7) | 96.4 (95.8-97.0) | 96.9 (96.3-97.5) | 97.2 (96.6-97.7) | 97.4 (96.8-97.9) | 5 |
| 35-39 | 87.4 (86.7-88.1) | 89.6 (88.9-90.3) | 91.6 (91.0-92.2) | 92.7 (92.2-93.3) | 93.8 (93.3-94.4) | 94.6 (94.1-95.1) | 95.2 (94.7-95.7) | 95.5 (95.0-96.0) | 95.9 (95.4-96.4) | 95.9 (95.4-96.4) | 95.9 (95.4-96.4) | 6 |
| **Diagnostic group**** |  |  |  |  |  |  |  |  |  |  |  |  |
| **CNS and other intracranial and intraspinal neoplasms** | 69.2 (65.1-73.0) | 70.7 (66.6-74.8) | 73.6 (69.4-77.8) | 72.5 (68.1-76.8) | 75.1 (70.7-79.5) | 74.8 (70.3-79.4) | 75.1 (70.4-79.8) | 76.0 (71.2-80.8) | 79.7 (74.9-84.4) | 79.1 (74.1-84.0) | 80.6 (75.6-85.6) | NA*** |
| **Sarcomas** |  |  |  |  |  |  |  |  |  |  |  |  |
| Soft tissue sarcomas | 84.3 (79.9-87.9) | 88.1 (84.5-91.7) | 93.9 (91.2-96.7) | 96.6 (94.5-98.7) | 95.8 (93.6-98.1) | 95.6 (93.2-97.9) | 96.8 (94.8-98.9) | 96.6 (94.5-98.7) | 96.3 (94.1-98.5) | 98.0 (96.2-99.7) | 98.1 (96.3-99.8) | 3 |
| Bone sarcomas | 87.0 (81.4-91.1) | 90.8 (86.7-95.0) | 93.1 (89.5-96.7) | 96.2 (93.5-99.0) | 96.4 (93.7-99.0) | 97.9 (95.8-100.0) | 98.4 (96.4-100.3) | 98.3 (96.4-100.3) | 98.4 (96.4-100.4) | 99.4 (97.9-100.9) | 99.4 (98.0-100.9) | 3 |
| Other sarcomas | 64.4 (51.4-74.7) | 82.4 (71.8-92.9) | 89.5 (80.7-98.4) | 93.7 (86.5-100.9) | 91.7 (83.6-99.7) | 94.0 (87.0-100.9) | 94.0 (87.0-101.0) | 94.0 (87.0-101.0) | 96.3 (90.7-101.9) | 96.4 (91.0-101.9) | 98.5 (94.5-102.4) | 8 |
| **Blood and lymphatic vessel tumors** | 68.9 (52.2-80.8) | 84.0 (71.0-97.1) | 96.0 (87.8-104.2) | 100.3 (100.2-100.3) | 96.4 (89.1-103.8) | 92.9 (82.9-102.8) | 92.9 (83.0-102.8) | 93.0 (83.0-102.9) | 89.5 (77.9-101.2) | 93.2 (83.4-103.0) | 96.9 (89.7-104.0) | 2**** |
| **Gonadal and related tumors** |  |  |  |  |  |  |  |  |  |  |  |  |
| Ovary, Germ cell and trophoblastic | 97.0 (91.9-99.0) | 98.6 (96.4-100.8) | 99.3 (97.7-100.9) | 100.2 (100.2-100.2) | 100.2 (-)****** | 100.2 (-)****** | 100.2 (-)****** | 100.3 (100.3-100.3) | 99.2 (97.1-101.3) | 99.2 (97.1-101.4) | 98.1 (95.0-101.2) | 0 |
| Ovary, Non-germ cell | 66.1 (60.8-70.8) | 70.9 (65.8-76.0) | 76.0 (70.9-81.1) | 82.0 (77.2-86.9) | 85.7 (81.0-90.4) | 90.9 (86.8-94.9) | 93.7 (90.1-97.2) | 94.6 (91.3-98.0) | 93.9 (90.4-97.4) | 96.0 (93.0-98.9) | 97.6 (95.4-99.9) | 9 |
| (Other) Germ cell, non-germ cell and trophoblastic tumors | 94.0 (84.5-97.8) | 98.6 (95.6-101.7) | 97.3 (93.3-101.3) | 97.3 (93.3-101.3) | 98.9 (96.2-101.6) | 98.9 (96.3-101.6) | 99.0 (96.3-101.6) | 98.8 (95.7-101.9) | 98.8 (95.8-101.9) | 98.9 (95.8-102.0) | 99.0 (95.9-102.0) | 1 |
| **Melanoma, malignant** | 97.8 (97.3-98.3) | 97.8 (97.3-98.3) | 98.0 (97.5-98.5) | 97.8 (97.3-98.3) | 97.9 (97.4-98.4) | 98.1 (97.7-98.6) | 98.2 (97.7-98.7) | 98.5 (98.1-99.0) | 98.7 (98.2-99.1) | 98.9 (98.5-99.3) | 99.0 (98.6-99.4) | 0 |
| **Carcinomas** |  |  |  |  |  |  |  |  |  |  |  |  |
| Thyroid carcinoma | 99.8 (99.2-100.0) | 99.9 (99.5-100.2) | 99.9 (99.5-100.2) | 100.0 (99.7-100.3) | 99.8 (99.4-100.2) | 99.8 (99.4-100.3) | 99.9 (99.5-100.4) | 99.7 (99.2-100.3) | 99.9 (99.4-100.4) | 99.9 (99.4-100.4) | 100.0 (99.4-100.5) | 0 |
| Carcinoma of head and neck | 89.0 (84.2-92.4) | 91.1 (87.4-94.9) | 93.9 (90.7-97.1) | 95.7 (93.0-98.5) | 97.2 (94.8-99.5) | 97.2 (94.9-99.5) | 96.7 (94.2-99.2) | 97.2 (94.8-99.6) | 96.7 (94.2-99.3) | 97.2 (94.8-99.6) | 97.7 (95.4-100.0) | 3 |
| Carcinoma of gastrointestinal tract |  |  |  |  |  |  |  |  |  |  |  |  |
| Carcinoma of stomach | 30.2 (22.3-38.5) | 52.5 (40.6-64.5) | 65.5 (52.3-78.8) | 78.7 (66.2-91.2) | 82.8 (71.0-94.5) | 89.4 (79.3-99.5) | 89.4 (79.3-99.5) | 97.6 (92.2-103.0) | 100.4 (-)****** | 100.5 (100.5-100.5) | 100.5 (100.5-100.5) | 7 |
| Carcinoma of colon | 68.0 (63.1-72.5) | 75.1 (70.4-79.8) | 82.5 (78.1-86.9) | 88.1 (84.2-92.0) | 91.7 (88.3-95.2) | 92.7 (89.4-96.1) | 96.2 (93.6-98.8) | 97.1 (94.7-99.4) | 97.1 (94.7-99.5) | 98.0 (95.9-100.1) | 98.9 (97.1-100.7) | 6 |
| Carcinoma of rectum | 63.8 (56.6-70.2) | 67.6 (60.7-74.6) | 73.2 (66.3-80.1) | 82.3 (76.1-88.6) | 88.7 (83.3-94.1) | 92.2 (87.6-96.9) | 95.9 (92.4-99.5) | 95.1 (91.2-99.0) | 94.4 (90.2-98.6) | 94.4 (90.1-98.6) | 95.0 (90.8-99.1) | 6**** |
| Carcinoma of liver and intrahepatic bile ducts (IBD) | 40.7 (26.9-54.2) | 63.9 (46.5-81.2) | 79.9 (61.9-97.8) | 79.9 (61.9-97.8) | 88.5 (73.0-103.9) | 94.0 (82.0-106.0) | 94.0 (82.0-106.0) | 100.4 (100.4-100.4) | 91.1 (73.8-108.4) | 91.1 (73.8-108.5) | 91.2 (73.8-108.5) | 7**** |
| Carcinoma of lung, bronchus, and trachea | 28.8 (22.7-35.2) | 43.0 (34.1-51.8) | 52.5 (42.1-62.8) | 60.3 (49.0-71.7) | 64.0 (52.3-75.6) | 76.2 (64.8-87.7) | 83.8 (73.3-94.3) | 89.1 (79.6-98.5) | 92.8 (84.3-101.3) | 95.2 (87.8-102.6) | 89.5 (79.0-99.9) | 9**** |
| Carcinoma of skin (if collected)***** | 98.0 (95.0-99.3) | 98.1 (96.1-100) | 97.9 (95.8-100) | 97.3 (94.9-99.7) | 97.3 (94.9-99.7) | 98.3 (96.2-100.4) | 97.8 (95.5-100.1) | 98.9 (97.1-100.7) | 99.4 (97.8-101.0) | 99.5 (97.9-101.1) | 99.5 (97.9-101.2) | 0 |
| Carcinoma of breast | 90.1 (89.4-90.8) | 89.3 (88.6-90.1) | 90.4 (89.7-91.2) | 91.3 (90.6-92.0) | 92.4 (91.7-93.1) | 93.0 (92.4-93.7) | 93.7 (93.1-94.4) | 94.0 (93.3-94.6) | 94.4 (93.7-95.0) | 94.3 (93.7-95.0) | 94.3 (93.6-95.0) | NA*** |
| Carcinoma of genital sites excluding ovary and testis |  |  |  |  |  |  |  |  |  |  |  |  |
| Carcinoma of uterine cervix | 90.6 (89.3-91.7) | 92.1 (90.9-93.2) | 94.6 (93.6-95.6) | 96.1 (95.2-97.0) | 97.1 (96.3-97.9) | 97.5 (96.7-98.3) | 97.5 (96.7-98.3) | 98.1 (97.3-98.8) | 98.4 (97.6-99.1) | 98.3 (97.6-99.1) | 98.5 (97.8-99.2) | 3 |
| Carcinoma of corpus uteri | 91.6 (85.5-95.3) | 94.7 (90.2-99.1) | 96.9 (93.1-100.7) | 96.9 (93.2-100.7) | 95.8 (91.4-100.2) | 96.9 (92.9-100.8) | 99.2 (96.8-101.7) | 96.5 (92.1-101.0) | 96.6 (92.2-101.0) | 97.9 (94.1-101.7) | 98.0 (94.2-101.8) | 2 |
| Carcinoma of vulva and vagina | 92.0 (85.5-95.7) | 92.7 (88.0-97.5) | 96.8 (93.3-100.2) | 97.6 (94.6-100.7) | 98.5 (96.0-101.1) | 98.3 (95.5-101.2) | 98.1 (94.9-101.3) | 98.2 (95.0-101.4) | 97.0 (92.9-101.0) | 97.0 (93.0-101.1) | 98.3 (94.8-101.7) | 2 |
| Carcinoma of urinary tract |  |  |  |  |  |  |  |  |  |  |  |  |
| Carcinoma of kidney | 89.0 (83.3-92.8) | 93.0 (89.0-97.0) | 95.2 (91.8-98.7) | 95.5 (92.0-99.0) | 98.1 (95.5-100.6) | 98.1 (95.5-100.7) | 98.9 (96.7-101.0) | 96.2 (92.4-99.9) | 96.8 (93.1-100.4) | 95.8 (91.7-99.9) | 95.9 (91.7-100.0) | 2 |
| Carcinoma of bladder | 48.2 (34.4-60.7) | 71.2 (55.9-86.5) | 82.6 (68.5-96.7) | 88.9 (76.6-101.1) | 88.9 (76.6-101.2) | 97.1 (90.6-103.5) | 97.1 (90.7-103.5) | 97.2 (90.7-103.6) | 100.6 (100.6-100.6) | 100.6 (100.6-100.6) | 100.7 (100.7-100.7) | 5 |
| Other invasive carcinomas | 39.7 (30.1-49.1) | 69.0 (56.3-81.8) | 69.1 (56.1-82.2) | 71.7 (58.6-84.8) | 75.1 (62.3-88.0) | 83.4 (71.9-94.9) | 93.0 (84.9-101.1) | 93.8 (86.5-101.2) | 94.1 (87.1-101.2) | 88.1 (78.7-97.5) | 88.2 (78.8-97.6) | NA*** |
| Neuroendocrine Tumors (NET) | 97.1 (94.9-98.4) | 97.7 (96.2-99.2) | 98.2 (96.8-99.6) | 98.9 (97.8-100.1) | 99.6 (98.7-100.5) | 99.9 (99.1-100.7) | 99.9 (99.2-100.7) | 100.0 (99.2-100.8) | 100.0 (99.3-100.8) | 100.5 (-)****** | 100.0 (98.9-101.1) | 0 |
| Miscellaneous specified neoplasms | 77.9 (63.5-87.2) | 87.1 (76.2-98.0) | 88.9 (78.4-99.4) | 96.8 (90.1-103.5) | 96.8 (90.1-103.5) | 100.3 (100.3-100.3) | 100.4 (-)****** | 100.4 (100.4-100.4) | 100.5 (-)****** | 95.3 (85.4-105.2) | 90.6 (77.6-103.7) | 3**** |

Abbreviation: CRS=Conditional Relative Survival, CI=Confidence Intervals, PE=Point-estimate, CNS=Central Nervous System, NA=Not Applicable.

* Estimates for the CRS for 0 years survived correspond with the five-year relative survival at the time of diagnosis.

** Cancer types were categorized into diagnostic groups according to the revised morphology based AYA-specific classification scheme developed by Barr et al. (2021). Cancer types were color coded into three groups based on the five-year CRS estimates: CRS>95% from diagnosis or within five years post-diagnosis (green), CRS>95% between five and ten years post-diagnosis (orange), and CRS<95% in ten years post-diagnosis (red).

*** NA was indicated whenever the CRS point-estimate did not surpass the 95% minimal excess mortality threshold in ten years post-diagnosis.

**** Passes the threshold in just one or a few non-consecutive years.

***** The Netherlands Cancer Registry does not collect data about basal-cell skin and lip carcinomas.

*******95% CI could not be calculated due to standard error calculation resulting in negative root numbers.
